# Permethrin-treated baby wraps for the prevention of malaria in children: Protocol for a double-blind, randomized placebo-controlled controlled trial in western Uganda

**DOI:** 10.1371/journal.pone.0284322

**Published:** 2023-04-27

**Authors:** Ross M. Boyce, Caitlin Cassidy, Ronnie Ndizeye, Emma Baguma, Dana Giandomenico, Bonnie E. Shook-Sa, Moses Ntaro, Raquel Reyes, Edgar M. Mulogo

**Affiliations:** 1 Institute for Global Health and Infectious Diseases, University of North Carolina at Chapel Hill, Chapel Hill, North Carolina, United States of America; 2 Department of Epidemiology, Gillings School of Global Public Health, University of North Carolina at Chapel Hill, Chapel Hill, North Carolina, United States of America; 3 Carolina Population Center, University of North Carolina at Chapel Hill, Chapel Hill, North Carolina, United States of America; 4 Department of Biostatistics, Gillings School of Global Public Health, University of North Carolina at Chapel Hill, Chapel Hill, North Carolina, United States of America; 5 Faculty of Medicine, Department of Community Health, Mbarara University of Science & Technology, Mbarara, Uganda; 6 Division of Hospital Medicine, UNC School of Medicine, University of North Carolina at Chapel Hill, Chapel Hill, NC, United States of America; University Hospital Heidelberg, GERMANY

## Abstract

This article details the study protocol for a double-blind, randomized placebo-controlled trial to determine the effectiveness of permethrin-treated baby wraps to prevent *Plasmodium falciparum* malaria infection in children 6–24 months of age. Participating mother-infant dyads will be randomized to receive either a permethrin-treated or a sham-treated wrap, known locally as a “*lesu*.” After a baseline home visit, during which time all participants will receive new long-lasting insecticidal nets, participants will attend scheduled clinic visits every two weeks for a period of 24 weeks. In the event of an acute febrile illness or other symptoms that may be consistent with malaria (e.g., poor feeding, headache, malaise), participants will be instructed to present to their respective study clinic for evaluation. The primary outcome of interest is the incidence of laboratory-confirmed, symptomatic malaria in participating children. Secondary outcomes of interest include: (1) change in children’s hemoglobin levels; (2) change in children’s growth parameters; (3) prevalence of asymptomatic parasitemia in children; (4) hospitalization for malaria in children; (5) change in the mother’s hemoglobin level; and (6) clinical malaria in the mother. Analyses will be conducted using a modified intent-to-treat approach, with woman-infant dyads who attend one or more clinic visits analyzed according to the arm to which they were randomly assigned. This is the first use of an insecticide-treated baby wrap for prevention of malaria in children. The study began recruitment in June 2022 and is ongoing. ClinicalTrials.gov Identifier: NCT05391230, Registered 25 May 2022.

## Introduction

Over the past two decades, the burden of *Plasmodium falciparum* malaria has substantially declined with mortality in endemic areas such as sub-Saharan Africa (SSA) decreasing by more than 35% [1]. The widespread deployment of vector control measures that target indoor-feeding and -resting *Anopheles* mosquitoes, such as long-lasting insecticidal nets (LLIN) and indoor residual spraying (IRS), largely account for these gains [2]. However, these strategies are generally insufficient to interrupt malaria transmission fully [3,4]. The degree of control that can be attained with LLINs or IRS is limited by a combination of factors including barriers to achieving and sustaining universal LLIN coverage [5–7], the resource-intensive nature of IRS programs [8,9], and the emergence of resistance to commonly-employed insecticides [10,11]. In addition, these household-based interventions can drive selection pressure [12]. For example, LLINs and IRS will favor mosquito behaviors that avoid these interventions, either by feeding on peri-domestic animals, outdoors, or in the early evening when residents are outside the home [13–16].

As evidence of these challenges, reports from the World Health Organization (WHO) suggest that progress against malaria has stalled and may even be slipping backwards in high-burden countries, particularly those in SSA [17]. Even at the peak of progress, however, malaria still accounted for approximately 400,000 deaths per year, with the vast majority occurring among children less than five years of age living in rural areas of SSA [17]. Uganda has one of the highest burdens of malaria, representing 5.4% of cases and 3.5% of deaths globally [17,18]. Despite progress, malaria still accounts for approximately 20% of outpatient visits and inpatient admissions [19]. Both malaria incidence and mortality were higher in 2020 than in 2015 in Uganda, resulting in the country being off track to achieve the Global Technical Strategy for Malaria milestones by 2030 [20]. Reasons for this include widespread rise in the prevalence of mosquitoes that are resistant to the first-line insecticides used in LLIN and IRS programs [11,21]. Furthermore, there is emerging evidence that primary malaria vectors, *A*. *gambiae* and *A*. *funestus* are increasingly exhibiting feeding behaviors that may not bring them into contact with existing interventions, while other vectors such as *A*. *arabiensis* are playing a larger role in transmission [22,23]. Thus, further innovations in malaria control are urgently needed [24–27].

In pursuit of this goal, we sought to leverage the traditional practice of mothers carrying young children on their backs utilizing wraps made from locally purchased cloth. The wrap, called a *lesu* in Uganda, also serves as a blanket or swaddle for children when they are put down or put to bed. Thus, mother and child spend much of the day in contact with the cloth. We hypothesized that when treated with an insecticide or repellent, the *lesu* might provide an additional layer of protection against malaria. Similar approaches have been widely utilized to treat LLINs and military uniforms for more than a decade [28–31], while treated blankets and tents have been shown to be highly effective in preventing malaria in refugee camps [32,33]. This approach has many potential advantages including: (i) targeting the most vulnerable (e.g., young children), (ii) integrating with existing cultural norms, and (iii) complementing current prevention strategies by offering protection against outdoor- and/or day-time biting *Anopheles* mosquitoes.

Here, we describe the protocol for a double-blind, randomized placebo-controlled trial for permethrin-treated *lesus* to prevent *P*. *falciparum* malaria in children 6–24 months of age in western Uganda. This trial builds on the work of a pilot feasibility study conducted in 2019, the results of which indicated that permethrin-treated baby wraps were well-tolerated and broadly acceptable, while adverse events were infrequent and mild [34]. These findings support the need for larger trials to evaluate the efficacy of the permethrin-treated *lesus*. The study will determine the effectiveness of permethrin-treated lesus to prevent P. falciparum malaria infection among infants and young children and their mothers. This protocol follows the guidelines set forth in the SPIRIT 2013 Statement defining standard protocol items for clinical trials [35].

## Materials and methods

### Overview

**Fig 1:**
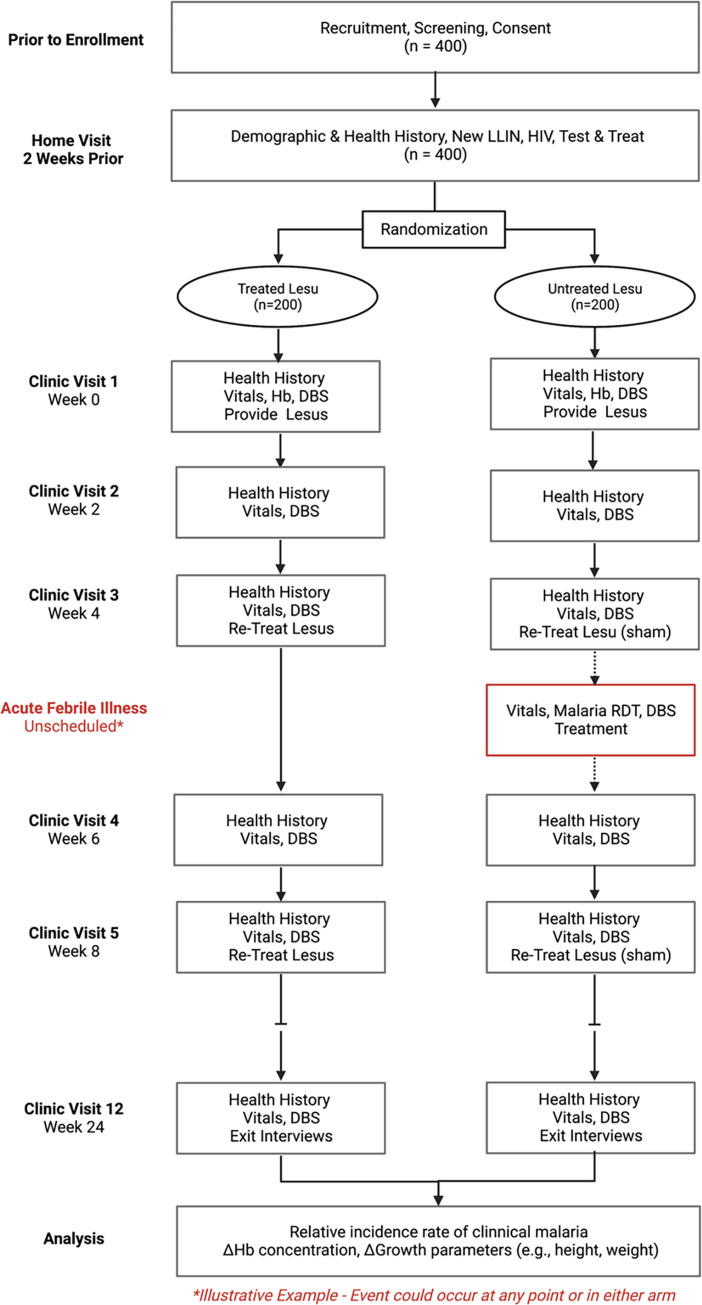
Overview of study schema with example of acute visit in the control group.

**Fig 2:**
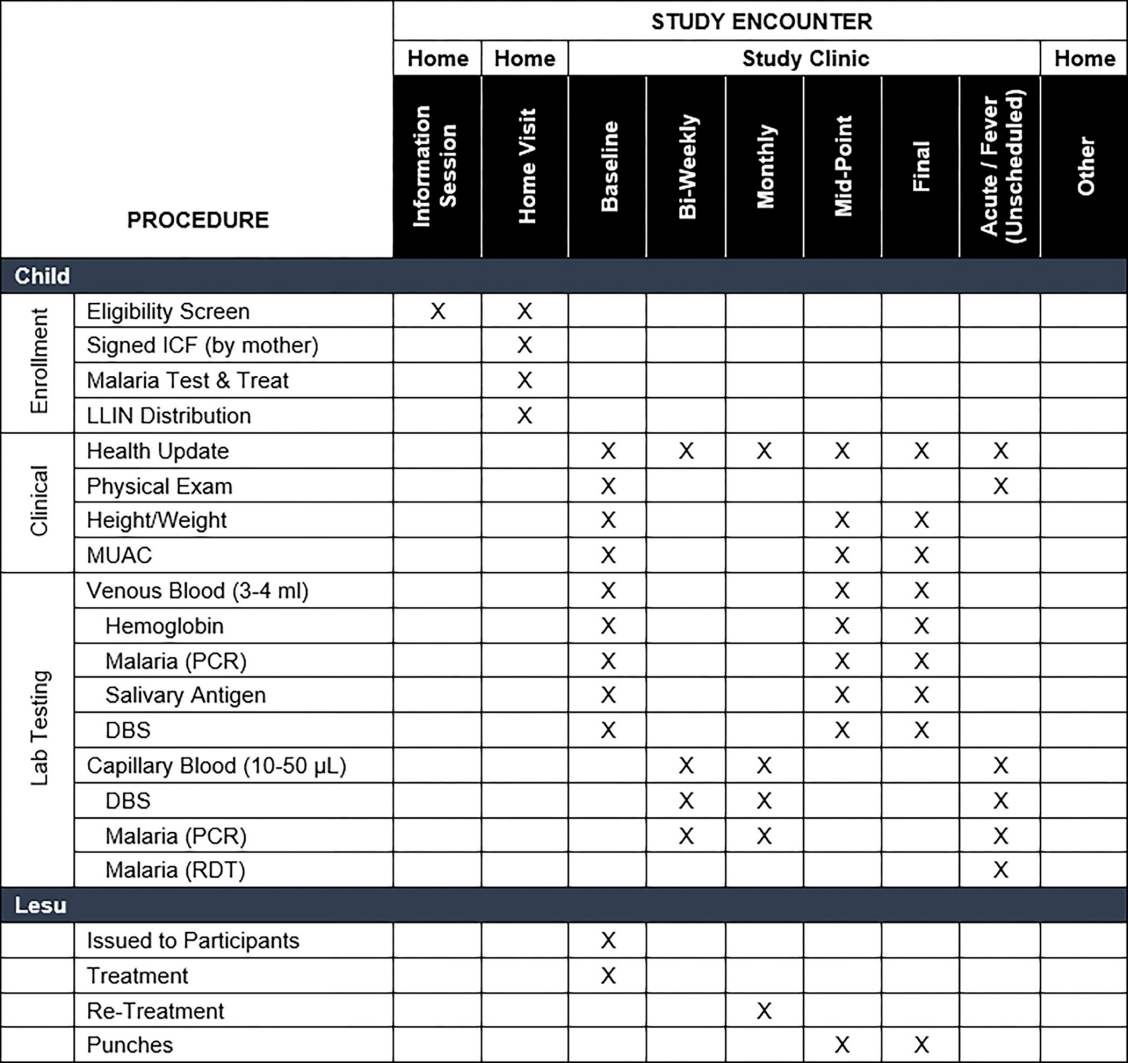
Modified SPIRIT checklist for participating children.

**Fig 3:**
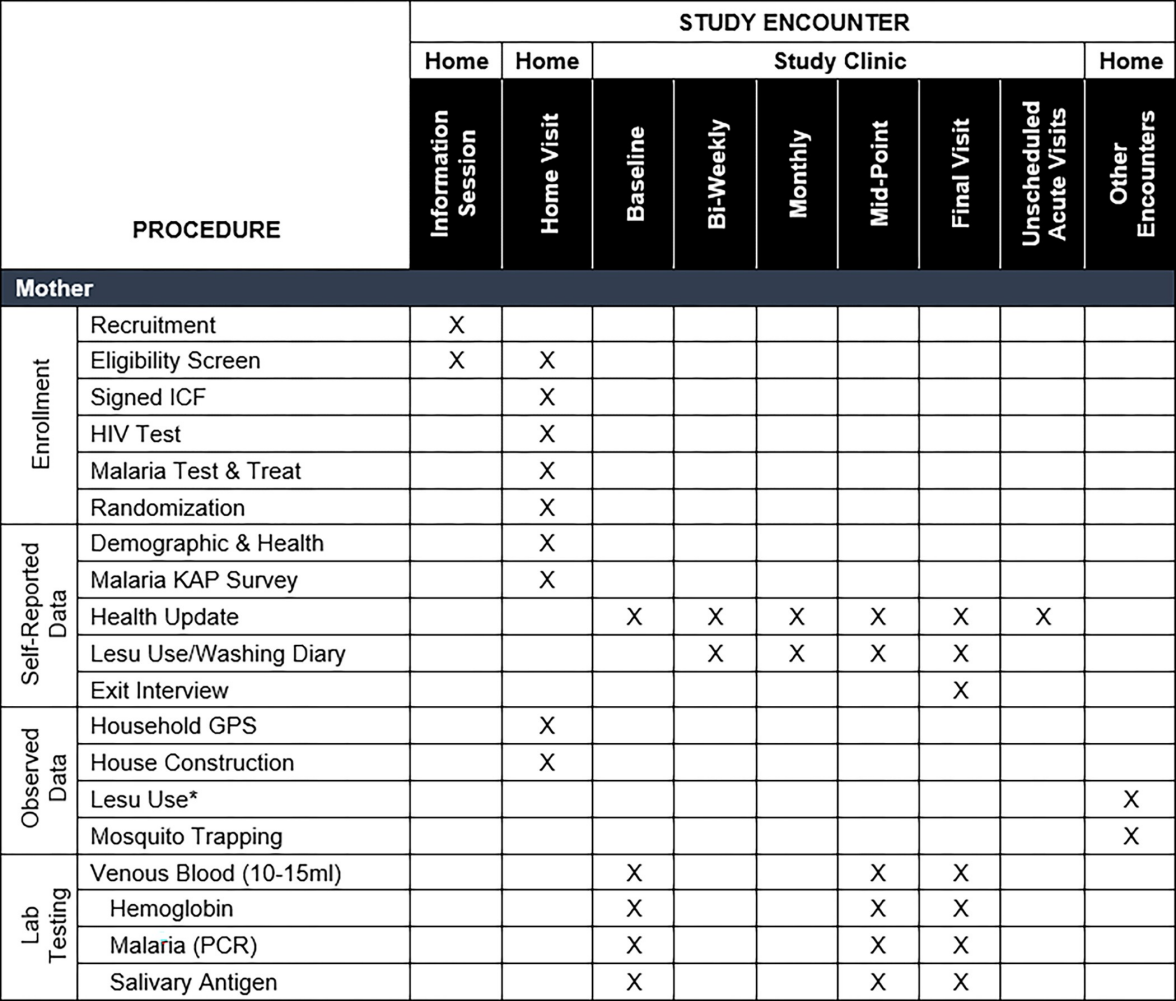
Modified SPIRIT checklist for participating mothers.

The study is a double-blind, randomized controlled trial of permethrin-treated *lesus* to prevent *P*. *falciparum* malaria in children 6–18 months of age at study enrollment conducted at two sites in rural western Uganda. Participants will be randomized to one of two arms and blinded to treatment assignment: permethrin-treated (intervention) or untreated (control) *lesus*. Participating mother-infant pairs will receive a new LLIN and two permethrin-treated or untreated *lesus* at the initial visit. The total sample size will be 400 mother-infant pairs with 200 pairs in each arm. We will follow participants longitudinally for six months. Participants will be instructed and incentivized to present to one of the two study clinics when a fever develops, where they will be evaluated, tested, and treated, if positive, for malaria. Participants will also attend scheduled clinic visits every two weeks for routine surveillance of adverse effects and to test for asymptomatic infection. Re-treatment and sham re-treatment of *lesus* will occur each month. Our primary outcome is rate of clinical malaria in children, defined as the presence of typical symptoms (e.g., fever, lethargy) and a positive malaria rapid diagnostic test (RDT) in each arm during the study period. The study schema is summarized in **Fig 1**, while the schedules of activities for participating children and mothers are summarized in **Figs 2** and **3**, respectively.

### Study aims

The overarching goal of this proposed project is to expand the malaria “toolbox” with a scalable, low-tech, low-cost intervention that leverages existing cultural practices and established insecticides to reduce the burden of *P*. *falciparum* malaria among young children in SSA. Our scientific objective is to demonstrate the protective effect of permethrin-treated *lesus* against *P*. *falciparum* malaria in infants and young children. Specifically, we propose to determine the effectiveness of permethrin-treated versus untreated *lesus* to prevent *P*. *falciparum* malaria infection among infants and young children and their mothers.

### Setting

The study will take place in the Bugoye and Maliba sub-counties and the town of Mubuku located in the Kasese District of western Uganda. Each of the sub-counties has a population of approximately 50,000 residents, with one-fifth of the population being children less than five years of age [36]. Subsistence farming (e.g. cassava, beans, maize) represents the primary economic activity, although a high proportion of households also keep livestock, including chickens (88%), goats (74%), and cattle (14%) in the peri-domestic space [37]. The vast majority of residents do not have access to water or electricity in the house, and one-third of households live ≥5 km to the nearest public health facility [36]. This population is generally representative of rural populations throughout malaria-endemic East Africa.

The climate in Bugoye permits year-round malaria transmission marked by semi-annual transmission peaks typically following the end of the rainy seasons in May and December [38]. The most recent malaria indicator surveys undertaken in the mid-western region (2014–15) and Tooro sub-national region (2018–19) which include the study area, reported *P*. *falciparum* parasitemia rates (PfPR) of 17.4% and 7.3%, respectively [39,40]. However, in a recent cross-sectional survey of more than 2,100 households in the Bugoye Sub-County, we found that the PfPR among children 2 to 8 years of age was upwards of 30% in many of the low-elevation villages [41]. We will recruit and enroll participants from these high transmission villages located along the river basins. The duration of study participation for each mother-child pair is six months, which is intended to incorporate potential differences in malaria transmission between dry and wet seasons.

### Eligibility criteria

Study inclusion criteria are the following:

- Willingness to provide informed consent
- Adult female age (≥18 years) with a child between 6 and 18 months of age
- Current residence in Bugoye, Maliba, or Mubuku

Study exclusion criteria are the following:

- Mother known to be HIV positive and taking cotrimoxazole
- Child who is known to have sickle cell disease or HIV,
- Known history of allergies to materials in insecticide-treated nets,
- Treatment with another investigational drug or intervention

### Ethical considerations & registration

Study procedures (current protocol version 1.0, date 17 December 2021) were approved by the WCG Institutional Review Board (1327369), the Mbarara University of Science and Technology (MUST) Research Ethics Committee (2021–297), and the Uganda National Council of Science and Technology. Participating individuals will be asked to provide written informed consent before undertaking any study-related activities. The study was prospectively registered at ClinicalTrials.gov (NCT05391230) in May 2022. Any amendment(s) to the protocol will require review and approval by the IRB before the changes are implemented in the study. Subject confidentiality is strictly held in trust by the investigators, study staff, and the sponsor(s) and their agents. Subject information will be maintained in a secure REDCap database.

### Recruitment

Prior to enrollment, we will hold a series of sensitization meetings with village leaders, community health workers, and clinical staff to describe the study’s aims and methods. Community health workers, each of whom is responsible for 20–30 households, will be asked to distribute study information to potentially eligible women in their respective coverage areas and will inform them of upcoming information sessions. Information sessions will be conducted at the study clinics or other community meeting areas during which time study staff fluent in the local language will review the study’s aims, methods, risks, and benefits. If attendees are interested in participating, they will be moved to a private area to ask additional questions. If study staff determine that a woman meets eligibility criteria, an initial household visit will be scheduled.

### Consent and enrollment

On the scheduled visit date, study staff will travel to the household, review the consent form, answer any questions the individual has, and ask the individual to provide written consent for participation. Participants will also be asked to provide consent for or decline long-term storage of specimens for future use. Participants may withdraw consent at any time throughout the course of the study. A negative rapid HIV test will be required for enrollment. If the result is positive, the mother will not be eligible to participate, but will be immediately linked to care. Once consented, study staff will complete baseline data collection as described below.

### Randomization procedures and blinding

Prior to the first clinic visit, participants will be randomized to either the permethrin-treated *lesu* or sham-treated *lesu* arms in a 1:1 ratio, stratified by age of the child (age 6–11 months and 12–18 months) and by study site (BHC and MHC) using a permuted block design with block sizes of 2, 4, and 6. The randomization scheme was developed prior to the beginning of study enrollment by a statistician. Randomization will be performed by an unblinded member of the study staff using the Randomization Module available in REDCap [42]. The unblinded members of the study staff will be responsible for treatment allocation as well as treatment or sham treatment of *lesus*. Study participants and clinical providers will remain blinded to allocation of the intervention until database lock. Unblinding of participants will only be authorized by the PI for safety concerns. If approved, the unblinded members of the study team will review the allocation and notify the relevant authority.

### Intervention

The intervention is a permethrin-treated *lesu*. Permethrin is a synthetic insecticide that acts on nerve cell membranes of the mosquito to disrupt the sodium channel current that regulates the polarization. This results in delayed repolarization, paralysis, and ultimately death of the mosquito. Permethrin also has a modest repellent effect, which may prevent biting even when mosquitoes are resistant to the killing effect [43]. This repellent property may be primary mechanism of effect of the intervention, especially given the widespread distribution of pyrethroid resistance among malaria vectors mosquitoes in Uganda [21,23].

Permethrin has been widely used to treat LLINs [44] and military uniforms [28–30] and remains the only repellant currently registered to treat fabric in the United States [45]. Permethrin treated clothing has a well-established safety record. Studies conducted among military personnel wearing permethrin-treated clothing demonstrated that exposure from chronic daily wear correlates with duration of exposure and is higher than the background exposure among the general population, but calculated daily exposures were still lower than exposure from topical pharmaceutical application [29,30]. In data taken from studies of the topical permethrin formulation used to treat pregnant women with scabies or lice, no increase in the risk of congenital abnormalities was noted among children exposed *in utero* [46,47]. The FDA classifies permethrin cream as Class B during pregnancy and the WHO considers permethrin compatible with breastfeeding [48].

Intervention *lesus* will be soaked in permethrin according to the manufacturer’s instructions. In brief, the *lesus* of participants in the intervention group will be treated and subsequently retreated each month with 0.5% permethrin (Sawyer Products, Safety Harbor, FL)—the same concentration approved by the U.S. Environmental Protection Agency (EPA) and used in military and civilian applications. While the proposed frequency of retreatment is greater than that used with early studies of insecticide-treated nets, our concern is that washing, especially if *lesus* were frequently soiled and washed using traditional methods, might cause premature washout of the permethrin. This has been reported in previous studies of permethrin-treated school uniforms when used to prevent dengue infection [49]. The increased frequency in retreatment is designed to minimize any reductions in efficacy due to washout. Because of the increased frequency in retreatment, we will test for potential adverse effects associated with persistent contact at optimal concentrations. Control *lesus* will undergo a sham treatment with water instead of permethrin. *Lesus* will be purchased from a local vendor and will all be of the same material, size, and design. There are no restrictions on concomitant use of alternative methods of vector control (e.g., LLIN, residual spraying) by participants during the course of the study.

### Questionnaire and surveys

#### Household visit

Immediately following enrollment, a study staff member will collect demographic and household characteristics from the participant via a questionnaire. A histidine rich protein-2 (HRP2) based malaria RDT will be performed on the mother and child, and if positive, the participant will receive weight-based treatment with artemther-lumefantrine in accordance with local guidelines for the treatment of malaria [50]. Before the end of the visit, a new LLIN will be provided to the mother with guidance that it should be used to protect the child. Upon conclusion of the survey, the study staff member will schedule the participants’ first clinic visit and provide the participant with a study ID card. The study staff will also record the household location using a handheld GPS or smartphone.

#### Lesu use

Participants will be asked to keep daily logs documenting the frequency of mother and child sleeping under the LLIN, *lesu* use throughout the day, and *lesu* washing frequency. The logs will be collected by study staff members at bimonthly clinic visits and will be used to assess compliance with the intervention. In addition, study staff will perform unscheduled, direct observation of a random sample of participants (25%) to document how and when *lesus* are used throughout the day with particular emphasis on the morning and evening periods. The study staff will also determine how participants wash the *lesus* and confirm that LLINs are being used. The study staff will visit each randomly sampled participant once at any point during the study. Participants will be selected using a systematic random sample generated by a statistician.

### Study visits

#### Baseline clinic visit

Participants will be scheduled for follow-up visits at the closest health center. During the baseline visit, study staff will administer a questionnaire reviewing changes in the participant’s medical history and interval care seeking since the initial household visit. The child’s vital signs, including height, weight, MUAC, and axillary temperature, will be collected and recorded. Venous blood will be collected from the mother and child to test for hemoglobin (Hb) concentration and to create dried blood spots (DBS) for future studies. At the end of the visit, study staff will issue two new permethrin-treated or untreated *lesus* to the mother according to randomization.

#### Follow-up visits

Participants will complete follow-up visits every two weeks (i.e, from Week 2 until Week 22). At each visit, a questionnaire will be administered to review medical changes since the last visit and adverse events will be recorded. The child’s vital signs will be collected and recorded, and capillary blood will be drawn from the child via finger prick or heel stick to create a DBS for later qPCR testing. *Lesus* will be retreated every 4 weeks (i.e., at every other bimonthly visit), either with permethrin or with a sham treatment, by an unblinded study staff member according to randomization. At the halfway point of the study (week 12), punches will be obtained from the *lesus* and stored for future testing of permethrin concentration, and venous blood draws will be obtained from the mother and child. Blood will also be tested for Hb concentration.

#### Final study visit

At the final study visit (week 24), study staff will administer a questionnaire to review medical changes since the last visit and any adverse events will be recorded. The child’s vital signs, including height, weight, MUAC, and axillary temperature, will be collected and recorded. Venous blood will be collected from the mother and child to test for Hb concentration and to create DBS for future studies. Punches will be obtained from the *lesus* and stored until transport to measure permethrin content. Lastly, study staff will conduct a semi-structured exit interview with the participant and provide the study completion incentive.

#### Unscheduled visits

In the event of any acute febrile illness or other symptoms that may be consistent with malaria (e.g., poor feeding, headache, malaise), participants will be instructed to present to their respective study clinic for evaluation. Upon registration, participants will be able present their study ID card to health center staff, who will notify study staff of the visit. Participants will undergo routine evaluation, testing, and treatment in accordance with local protocols. At the conclusion of the visit, a member of clinic staff will interview the patient to document symptoms and will review clinical and laboratory registers to abstract relevant information.

### Laboratory testing

#### HIV screening

Testing for HIV will be performed at the initial household visit with a rapid diagnostic test (SD Bioline HIV-1/2 3.0 Abbott Laboratories, USA) as individuals living with HIV and HIV-exposed, uninfected infants should receive cotrimoxazole preventive therapy [51], which in addition to its antibacterial benefits, also has well-established anti-malarial properties [52,53]. Approximately 20 μL of whole blood will be collected via finger-prick and placed onto the RDT according to the manufacturer’s instructions.

#### Malaria

Testing for malaria will be performed at the initial household visit with an RDT (SD Bioline Malaria Ag P.f, Abbott Laboratories, USA or similar pending availability). Approximately 100 μL of whole blood will be collected via finger-prick or heel stick and placed onto the RDT according to the manufacturer’s instructions. This test detects histidine-rich protein II antigen specific to *P*. *falciparum* malaria. Similar assays are currently employed for routine diagnosis in Uganda

#### Hemoglobin

Hb levels that will be collected during clinic visits will be measured at the study clinics using the HemoCue® Hb 201+ analyzer (Brea, California) [54,55]. Approximately 10 μL of whole blood will be placed onto the microcuvette according to the manufacturer’s instructions.

#### Asymptomatic parasitemia

Detection of asymptomatic parasitemia was conducted using DBS. DBS were stored in a mylar bag with desiccant until transport to Epicentre Research Laboratory in Mbarara. *Plasmodium* species DNA will be extracted from DBS using a previously described protocols [56]. The concentration of extracted *P*. *falciparum* DNA in individual samples will be determined using qPCR for *P*. *falciparum* lactate dehydrogenase [57].

#### Permethrin concentration

To evaluate permethrin concentrations, collected fabric punches will be placed in individual mylar bags and stored in an opaque container at 20°C until transport to East Carolina University. Swatches will then be transferred to individual 60 mL amber glass vials containing 40 mL acetone and soaked for 6 hours to elute permethrin. A portion of the extract (1 pL) will be analyzed directly by capillary GC with flame ionization detector using an Agilent GC 6850 in accordance with previously published protocols [58].

### Study monitoring and participant withdrawal

In addition to the PI’s responsibility for oversight, study oversight will be under the direction of the Data Safety and Monitoring Board (DSMB) at the North Carolina Translational & Clinical Sciences Institute (TraCS). The DSMB is independent of the study and will be available in real time to review and recommend appropriate action regarding adverse events and other safety issues will review enrollment reports every 6 months and full data reports annually. Any moderate or severe adverse events related to study participation should be reported promptly and an ad hoc review be conducted, if deemed necessary. Reviewed data will be separated by study arm and provided by a statistician with access to the unblinded data. The occurrence of any severe adverse event or at least five study-related moderate adverse events will prompt a temporary suspension of enrollment while an ad hoc safety review is convened.

A subject’s participation in the study may be discontinued by the PI if: (1) any clinical adverse event, laboratory abnormality, or other medical condition or situation occurs such that continued participation in the study would not be in the best interest of the subject, (2) the subject meets an exclusion criterion (either newly developed or not previously recognized) that precludes further study participation, or (3) the subject is lost to follow-up.

### Data management and statistical analysis

#### Outcomes

Our primary outcome of interest is incidence of clinical malaria in children, defined as the presence of typical symptoms (e.g., fever, lethargy) and a positive malaria RDT during observation. Secondary outcomes of interest include: (1) change in children’s Hb levels; (2) change in children’s growth; (3) asymptomatic parasitemia in children; (4) hospitalization for malaria in children; (5) change in the mother’s Hb level; and (6) clinical malaria in the mother. Exploratory outcomes include frequency of *lesu* use and washing as well as residual permethrin levels on the *lesus*. Safety measures to be evaluated include: adverse reactions to treated *lesus* and SAEs (**Table 1**).

**Table 1:** Summary of key outcomes and collected co-variates of interest.

Study Measure	Method of Collection	Collected in Pilot
1. Primary Outcome		
1.A. Incidence of clinical malaria in child	(1) Acute febrile illness AND positive RDT at schedule or unscheduled study clinic visit - or - (2) Written documentation of fever AND positive RDT from non-study clinic visit	No
2. Secondary Outcomes		
2.A. Change in child’s Hb level	Hemoglobin measured at baseline (Week 0), Week 12, and Week 24 visits using Hemocue device.	Yes
2.B. Change in child’s growth	MUAC measured at baseline (Week 0), Week 12, and Week 24 visits [59].	Yes
2.C. Asymptomatic parasitemia in children	Presence of malaria parasites on bi-weekly dried blood spot (DBS) as determined by qPCR.	Yes
2.D. Hospitalization for malaria	Self-reported history of hospitalization on bi-weekly questionnaire AND confirmation by study staff	No
2.E. Clinical malaria in mother	Same as 1.A.	No
2.F. Change in mother’s Hb level	Same as 2.A.	Yes
3. Safety Measures		
3.A. Adverse reaction to treated-lesu	Self-reported history on bi-weekly questionnaire and confirmation by clinical staff.	Yes
3.B. Severe adverse event (SAE)	Care-seeking, hospitalization, or death attributable to the intervention.	Yes
4. Covariates of Interest		
3.A. Socioeconomic status	Questions from baseline survey on ownership of mobile phone, vehicle, etc. [60]	Yes
3.B. Maternal education	Self-reported history on baseline questionnaire	Yes
3.C. Frequency of lesu use and washing	Self-reported history on bi-weekly questionnaire	Yes
3.D. Residual permethrin levels	Punches of lesu cloth taken at 2-, 4-, 6-month visits tested by gas chromatography [58]	No

#### Sample size considerations

The sample size for the study was selected to provide sufficient power to evaluate the primary study hypothesis. Assuming a baseline risk of approximately 3.0 cases per 100 person-weeks as estimated from our pilot study, the trial will have a power of at least 0.80 to detect a 30% relative reduction in the incidence rate of malaria between intervention and control groups over the proposed study period of 24 weeks with a type I error rate of 0.05. Calculations were conducted using nQuery software and are based on previously described methods [61]. No interim analyses are proposed, so no adjustments were made to these calculations to account for interim testing. We plan to recruit and enroll 400 mother-infant pairs. Additional participants will be enrolled in the study to replace those who drop out before 12 weeks. Those who drop out after 12 weeks will not be replaced. All participants who attend any clinic visits post randomization will be included in the modified intention-to-treat (ITT) analysis.

#### Data management

All data collected at study visits will be entered into portable tablet devices equipped with wireless internet and uploaded each day to a secure REDCap project. Data quality checks have been built into REDCap questionnaires (e.g., range of plausible values). Each week, new entries will be reviewed by a research assistant and any errors or incomplete entries will be forwarded to the field team for correction.

#### Planned statistical analyses

Analyses will be conducted using a modified intent-to-treat (ITT) approach, with woman-infant pairs analyzed according to the arm to which they were randomly assigned regardless of their subsequent use or non-use of the *lesus* and dropout after at least one clinic visit. An α = 0.05 significance level will be used throughout, with corresponding 95% confidence intervals (CIs). No adjustment for multiple testing will be made for the primary outcome. For secondary outcomes (excluding safety endpoints), we will control the false discovery rate using methods proposed by Benjamini and Hochberg [62].

Given the high level of subject participation and retention in the pilot study [34], we expect most mother-infant pairs to complete all study activities. Missing data due to dropout or missed clinic visits are anticipated to be uncommon (≤5% missing), so a complete case analysis is planned. If >10% of participants are missing data in either arm, a sensitivity analysis will be conducted using multiple imputation to account for data missing at random (missing conditional upon measured baseline covariates and study outcomes). If data are suspected to be missing not at random, this will be described with the study results as a limitation.

In the primary analysis, the rates of clinical *P*. *falciparum* malaria in children 6–24 months of age will be compared between treatment arms by estimating the incidence rate ratio and a corresponding 95% CI (with the control arm as the referent group) of malaria using a Poisson regression model with robust variance estimation. The Poisson model will be adjusted to control for stratification variables from randomization (study site and age group) and will include an offset account for varying person-time-at-risk across subjects. In exploratory analyses, we will further evaluate our primary outcome by fitting additional Poisson regression models to estimate associations between the incidence of malaria and measured covariates, including the frequency of *lesu* use and washing and residual permethrin concentration among participants in the intervention arm. If *lesu* use is lower than anticipated, exploratory analyses may also be conducted using a per-protocol approach, with woman-infant pairs analyzed according to *lesu* use as documented in the daily logs. In per-protocol analyses, Marginal structural models will be used to estimate an “as-treated” effect of permethrin-treated *lesus* on the primary endpoint.

Sensitivity analyses will be conducted using time-to-event approaches. Cox proportional hazards models will be used to estimate time to first malaria infection. Time to recurrent malaria infections will be analyzed using the Andersen-Gill extension of the Cox model with clustering to adjust for repeated measures and with robust variance estimation. As in the primary analyses, time-to-event models will control for the stratification variables from randomization.

Secondary outcomes, including asymptomatic parasitemia in children and clinical malaria in mothers, will be analyzed using the same methods as the primary analysis of the primary endpoint. Other secondary outcomes, including change in child’s Hb level and growth as well as change in mother’s Hb level, will be evaluated by comparing median changes, with corresponding 95% CIs, between study groups at 12 and 24 weeks using the Wilcoxon Rank Sum test. Lastly, for the secondary outcome of hospitalization for malaria, the number of hospitalizations for malaria in each arm will be compared using Fisher’s exact test. The proportion of hospitalizations and the difference in proportions between randomization arms will be calculated and corresponding CIs will be constructed using Clopper-Pearson exact binomial CIs.

Descriptive analyses will be used to summarize adverse reactions to treated-*lesu* and SAEs, by treatment arm. For each arm, a 95% 1-sided Clopper-Pearson exact binomial upper confidence limit for the probability of an SAE will be calculated. If sample sizes allow, the confidence limits will also be calculated by the children’s age group.

#### Data availability

Deidentified individual data that supports the results will be shared beginning 9 to 36 months following publication provided the investigator who proposes to use the data has approval from an Institutional Review Board (IRB), Independent Ethics Committee (IEC), or Research Ethics Board (REB), as applicable, and executes a data use/sharing agreement with UNC.

#### Dissemination

Trial results will be communicated through various conference presentations and peer-reviewed manuscripts in open-access journals. In addition, at the conclusion of the trial, a formal dissemination event will be held with participants and key community stakeholders.

## Results

Study recruitment began in June 2022 and is ongoing.

## Discussion

Progress against malaria has stalled and may even be slipping backwards in high-burden countries [17] and further innovations in malaria control are urgently needed [24–27,63]. In response, we are exploring a novel that leverages the traditional practice of mothers carrying young children on their backs utilizing wraps made from locally-purchased cloth. This randomized clinical trial is the first study to test the effectiveness of insecticide-treated baby wraps to prevent malaria infection in infants and young children. This approach has several advantages, including: (i) targeting young children, the group most susceptible to severe malaria and death from *P*. *falciparum* infection, (ii) integrating with existing cultural practices, and (iii) complementing current prevention strategies by offering protection against *Anopheles* mosquitoes, that are increasingly biting outdoors and during the day and thus may not be well-targeted by other interventions.

There are several strengths to our approach including the rigorous study design, which incorporates the use of randomization and blinding via sham-treatment, which allows us to better determine the true effect of a permethrin-treated *lesu*, as opposed to an untreated one. Furthermore, our sample size employed relatively conservative estimates of infection and therefore, we anticipate that the study will be well-powered to estimate our primary outcome. Lastly, most of the methods employed herein were previously tested and validated in our pilot study [34], which should reduce errors in data collection.

The study also has some limitations, foremost of which is the lack of entomological measures that would allow us to better understand the direct effect of the permethrin-treated wraps on mosquito landing and biting. Additionally, while we are collecting self-reported information on daily *lesu* use and frequency of washing, we cannot directly measure these important variables. We will attempt to directly observe a sample of participants, but the results may still suffer from potential desirability bias in the reports of use.

There are a number of additional investigations that may arise from this work, including both sub-studies and future studies. For example, while we are not collecting entomological measures, it may be possible to compare baseline and end visit samples using *Anopheles* mosquito salivary antigen (e.g., gSG6-P1) as a surrogate measure of landing and biting between arms [64]. In addition, we are exploring opportunities to collect adult mosquitoes in the peridomestic space of participants to assess the prevalence of molecular markers of pyrethroid resistance. If our hypothesis is correct and permethrin treated *lesus* reduce the incidence of malaria among children, further studies of treatment frequency and methods, including bonding directly to the cloth or home-treatment options, will be appropriate. The basic concept and study design can also be employed to test other insecticides and repellents.

## Conclusions

This article describes the protocol for a double-blind, randomized controlled trial that will determine the efficacy of permethrin-treated *lesus* for reducing malaria infections in children in western Uganda. Results of the study will inform future investigations and potentially lead to a new in tool in the “toolbox” to reduce morbidity and mortality from *P*. *falciparum* malaria.

## Supporting information

S1 Checklist SPIRIT 2013 Checklist: Recommended items to address in a clinical trial protocol and related documents*. (DOC)

S1 File (PDF)

S2 File (DOCX)
