# Supplementary material for: Permethrin-treated baby wraps for the prevention of malaria in children: Protocol for a double-blind, randomized placebo-controlled controlled trial in western Uganda
Source: PLoS One. 2023 Apr 27;18(4):e0284322. doi: 10.1371/journal.pone.0284322 (PMC10138219; doi:10.1371/journal.pone.0284322)
Supplement: S1 File — (PDF) [file pone.0284322.s002.pdf]

Data Dictionary Codebook

12/20/2022 2:51pm

#	Variable / Field Name	Field Label<br>*Field Note*	Field Attributes (Field Type, Validation, Choices, Calculations, etc.)
**Instrument: 1. Screening Form (screening_form)**			
[illegible]	study_id	REDCap ID should match ID card provided to participant (Ex BUG-001)<br>*Ensure matches ID card*	text
2	screen_date	Section Header: *1. ADMINISTRATIVE INFORMATION*<br>1.A. Date of Screening	text (date_dmy, Min: 2022-01-01, Max: 2024-12-31), Required, Identifier
3	screen_name	1.B. Name of Screener	dropdown, Required, Identifier<br>0 Ronnie Ndizeye<br>1 RA #1<br>2 RA #2<br>3 Other
4	screen_name_other<br>Show the field ONLY if:<br>[screen_name] = '3'	If other, please list name here:	text, Identifier
5	screen_subcounty	Section Header: *2. ELIGIBILITY SCREEN*<br>2.A. Does the mother live in Bugoye, Maliba, or Mubuku Sub-County?	yesno, Required<br>1 Yes<br>0 No
6	screen_distance	2.B. Does the mother live within approximately 30 minutes (walking distance) of either Bugoye or Mukathi Health Centres?	yesno, Required<br>1 Yes<br>0 No
7	screen_hiv	2.C. Is the mother known to be HIV positive?	yesno, Required<br>1 Yes<br>0 No
8	screen_age	2.D. Does the mother have a child between the ages of 6 - 18 months?	yesno, Required<br>1 Yes<br>0 No
9	screen_scd	2.E. Is the child known to have sickle cell disease or HIV?	yesno, Required<br>1 Yes<br>0 No
10	screen_allergy	2.F. Does the mother or child have any history of allergies to material in insecticide-treated nets?	yesno, Required<br>1 Yes<br>0 No
11	screen_research	2.G. Is the mother or child currently participating in any other research studies?	yesno, Required<br>1 Yes<br>0 No
12	screen_research_yes<br>Show the field ONLY if:<br>[screen_research] = '1'	If participating in another research study, what is the nature of the study?	notes
13	screen_elig	Section Header: *3. ELIGIBILITY DETERMINATION*	descriptive

	Show the field ONLY if:<br>[screen_subcounty] = '1' and [screen_distance] = '1' and [screen_hiv] = '0' and [screen_age] = '1' and [screen_scd] = '0' and [screen_allergy] = '0'	POTENTIALLY ELIGIBLE Gather contact information to schedule household visit	
14	screen_ineligible<br>Show the field ONLY if:<br>[screen_subcounty] = '0' or [screen_distance] = '0' or [screen_hiv] = '1' or [screen_age] = '0' or [screen_scd] = '1' or [screen_allergy] = '1'	NOT ELIGIBLE Thank individual for interest	descriptive
15	screen_final	3.A. Screening Outcome	radio<br>0 Not eligible<br>1 Potentially eligible
16	screening_form_complete	Section Header: *Form Status*<br>Complete?	dropdown<br>0 Incomplete<br>1 Unverified<br>2 Complete
**Instrument: 2. Registration Form (registration_form)**			
17	regis_intro	SCRIPTThank you for answering those questions. It appears you may be eligible to participate in the study. As a next step, we would need to collect a little information about you to schedule the household visit. You will not be officially enrolled in the study until this visit is completed. We will contact you by phone to arrange the best time.	descriptive
18	reg_first	Given Name / First Name	text, Required, Identifier
19	reg_last	Surname / Last Name	text, Required, Identifier
20	regis_dob	Date of Birth	text (date_dmy, Min: 1970-01-01, Max: 2004-01-01), Required, Identifier
21	regis_phone_yn	Do you have a mobile phone?	yesno, Required<br>1 Yes<br>0 No
22	regis_phonenum<br>Show the field ONLY if:<br>[regis_phone_yn] = '1'	What is the best phone number to reach you?<br>*No spaces*	text (integer), Identifier
23	regis_phone_alt<br>Show the field ONLY if:<br>[regis_phone_yn] = '0'	If you do not own a phone, do you have access to a phone (family member, neighbor) where we could reach you?	yesno<br>1 Yes<br>0 No
24	regis_phone_altnum<br>Show the field ONLY if:<br>[regis_phone_alt] = '1'	What is the phone number where we can reach you?<br>*No spaces*	text
25	regis_other<br>Show the field ONLY if:<br>[regis_phone_alt] = '0'	If you do not have access to a phone, how would you prefer that we contact you to schedule the visit?	notes
26	registration_form_complete	Section Header: *Form Status*<br>Complete?	dropdown<br>0 Incomplete<br>1 Unverified

			2 Complete
**Instrument: 3. Consent Documentation (consent_documentation)**			
27	consent_date_review	Date consent form reviewed with individual	text (date_dmy, Min: 2022-01-01, Max: 2024-12-31), Required, Identifier
28	consent_yn	Did the individual agree to participate in the study?	yesno, Required<br>1 Yes<br>0 No
29	consent_decline<br>Show the field ONLY if:<br>[consent_yn] = '0'	If the individual did not agree to participate, what was the reason given, if any?	notes
30	consent_language	Section Header: *Consent Checklist*<br>Was consent obtained in a language the participant understood?	radio (Matrix), Required<br>1 Yes<br>0 No
31	consent_risks	Were study risks list reviewed with the participant?	radio (Matrix), Required<br>1 Yes<br>0 No
32	consent_response	Were responsibilities of the participant reviewed?	radio (Matrix), Required<br>1 Yes<br>0 No
33	consent_questions	Did the study team member explain and answer participant's questions?	radio (Matrix), Required<br>1 Yes<br>0 No
34	consent_screening	Was the Main ICF signed & witnessed prior to HIV testing?	radio (Matrix), Required<br>1 Yes<br>0 No
35	consent_text	Does the participant agree to be contacted by text message?	radio (Matrix), Required<br>1 Yes<br>0 No
36	consent_copy	Did the participant request a copy of the signed Main ICF?	radio (Matrix), Required<br>1 Yes<br>0 No
37	consent_version	Verify version of consent form that was used	dropdown<br>0 Version 1.0 (Current as of 22 SEP 21)<br>1 Version 2.0 (TBD)<br>2 Version 3.0 (TBD)
38	consent_hiv_res	What was the result of the mother's HIV test	radio, Required<br>0 Negative<br>1 Positive
39	consent_icf_upload<br>Show the field ONLY if:<br>[consent_yn] = '1'	Upload scanned INFORMED CONSENT form.	file, Required, Identifier
40	consent_storage_yn	Did the participant consent for long-term storage of specimens	yesno, Required<br>1 Yes<br>0 No

41	consent_storage_upload<br>Show the field ONLY if:<br>[consent_storage_yn] = '1'	Upload scanned CONSENT FOR STORAGE form	file, Required, Identifier
42	consent_documentation_complete	Section Header: *Form Status*<br>Complete?	dropdown<br>0 Incomplete<br>1 Unverified<br>2 Complete
**Instrument: 4. Household Visit - Demographics (household_visit_demographics)**			
43	hh_unique	Section Header: *Section 4.A.Administrative Information*<br>4.A.1. Unique IdentifierFirst Letter Given Name - First Letter Surname - First Two Letters of Village - Year of Birth Example: Susan Muhindo from Izinga born in 1984 = SMIZ84	text, Required, Identifier
44	hh_date	4.A.2. Date of Visit	text (date_dmy, Min: 2022-01-01, Max: 2024-12-31), Required
45	birth_date	Section Header: *Section 4.B. Mother Demographic & Health Information*<br>4.B.1. What is your date of birth?	text (date_dmy), Required, Identifier
46	age_years	4.B.2. How old were you at your last birthday?<br>*age in completed years*	text (number, Min: 18, Max: 65), Identifier
47	school_yn	4.B.3. Have you ever attended school?	yesno<br>1 Yes<br>0 No
48	school_highestlevel<br>Show the field ONLY if:<br>[school_yn] = '1'	What is the highest level of school you attended?	radio<br>1 Primary<br>2 'O' Level<br>3 'A' Level<br>4 University/Tertiary
49	hh1_read_card	4.B.4. Now I would like you to read this sentence to me.SHOW CARD TO RESPONDENT.IF RESPONDENT CANNOT READ WHOLE SENTENCE, PROBE:Can you read any part of the sentence to me?	radio<br>1 Cannot read at all<br>2 Able to read only parts of sentence<br>3 Able to read whole sentence<br>4 No card with required language<br>5 Blind/Visually impaired
50	hh1_religion	4.B.5. What is your preferred religion?	radio<br>1 Catholic<br>2 Anglican/Protestant<br>3 Seventh Day Adventist<br>4 Pentecostal<br>5 Other Christian<br>6 Moslem<br>7 Bahai<br>8 Traditional<br>9 Hindu<br>10 None<br>99 Other
51	hh1_religion_other	If other religion, please specify	notes

	Show the field ONLY if: [hh1_religion] = '99'		
52	hh1_ethnic	4.B.6. What is your ethnic group?	radio<br>1 Baganda<br>2 Banyankore<br>3 Iteso<br>4 Lugbara/Madi<br>5 Basoga<br>6 Langi<br>7 Bakiga<br>8 Karimojong<br>9 Acholi<br>10 Bagisu/Sabiny<br>11 Alur/Jopadhola<br>12 Banyoro<br>13 Batoro<br>99 Other
53	hh1_ethnicity_other<br>Show the field ONLY if: [hh1_ethnic] = '99'	If other ethnic group is selected, please specify:	notes
54	hh1_meds	4.B.7. Do you take any medications every day?	yesno, Required<br>1 Yes<br>0 No
55	hh1_meds_spec<br>Show the field ONLY if: [hh1_meds] = '1'	If you do take any medications, please list each one here:Separate each with a comma	notes
56	hh1_hospitalization	4.B.8. Other than for the birth of your children, have you ever had to stay overnight in a hospital or health centre?	yesno<br>1 Yes<br>0 No
57	hh1_hospitalization_why<br>Show the field ONLY if: [hh1_hospitalization] = '1'	What was the reason you had to stay in the hospital or health centre?If multiple events, please describe the most recent reason.	radio<br>1 Malaria<br>2 Fever, not malaria<br>3 Diarrhea<br>4 Breathing, cough, or pneumonia<br>5 Injury<br>99 Other
58	hh1_hospitalization_other<br>Show the field ONLY if: [hh1_hospitalization_why] = '99'	If another reason for hospitalization, please specify:	notes
59	hh1_martial	Section Header: *Section 4.C. Family & Reproduction*<br>4.C.1. What is your marital status?	radio<br>0 Married<br>1 Never Married<br>2 Divorced

			3 Widowed<br>99 Other
60	hh1_martial_other<br><br>Show the field ONLY if:<br>[hh1_martial] = '99'	If other martial status selected, please specify	notes
61	hh1_husband_age<br><br>Show the field ONLY if:<br>[hh1_martial] = '0'	If married, how old is your husband?<br>*Years*	text (integer, Min: 18, Max: 99)
62	hh1_births_intro	Now I would like to ask about your birth history and children	descriptive
63	hh1_births	4.C.2. How many times have you given birth?	text (integer, Min: 1, Max: 20)
64	hh1_birth_laterdied_yn	4.C.3. Have you ever given birth to a boy or girl who was born alive but later died?	yesno<br>1 Yes<br>0 No
65	hh1_sons_laterdied<br><br>Show the field ONLY if:<br>[hh1_birth_laterdied_yn] = '1'	How many boys have died?IF NONE, RECORD '00'.	text (integer, Min: 0)
66	hh1_daughters_laterdied<br><br>Show the field ONLY if:<br>[hh1_birth_laterdied_yn] = '1'	And how many girls have died?IF NONE, RECORD '00'.	text (integer, Min: 0)
67	hh1_children_alive	4.C.4. How many living children do you have?	text (integer, Min: 1, Max: 20), Required
68	hh1_children_oldest	4.C.5. How old is your oldest child?IF ONLY ONE CHILD (THE PARTICIPANT) ENTER "1"<br>*Years*	text (integer, Min: 0, Max: 35)
69	hh1_children_deliveryhc	4.C.6. How many of your children were born at a health facility?	text (integer, Min: 0, Max: 20)
70	hh1_children_deliveryhome	4.C.7. How many of your children were born at home?	text (integer, Min: 0, Max: 20)
71	hh1_preg_intro	Now I would like to ask you about your most recent pregnancy.	descriptive
72	hh1_antenatal_yn	4.C.8. When you were pregnant, did you see anyone for antenatal care?	yesno<br>1 Yes<br>0 No
73	hh1_anc_provider<br><br>Show the field ONLY if:<br>[hh1_antenatal_yn] = '1'	Whom did you see?<br>*Select all that apply*	checkbox<br>1 hh1_anc_provider___1 Doctor<br>2 hh1_anc_provider___2 Nurse/Midwife<br>3 hh1_anc_provider___3 Medical Assistant/Clinical Officer<br>4 hh1_anc_provider___4 Nursing Aide<br>5 hh1_anc_provider___5 Traditional Birth Attendant<br>99 hh1_anc_provider___99 Other
74	hh1_anc_provider_other<br><br>Show the field ONLY if:<br>[hh1_anc_provider(99)] = '1'	If another type of provider, please specify:	notes
75	hh1_anc_location<br><br>Show the field ONLY if:<br>[hh1_antenatal_yn] = '1'	Where did you receive most of your antenatal care?	radio<br>0 Public Health Clinic (Level 2 or Level 3)<br>1 Government Hospital

			2 Private Facility<br>3 In home (midwife or birth assistant)<br>99 Other
76	hh1_anc_location_other<br><br>Show the field ONLY if:<br>[hh1_anc_location] = '99'	If most antenatal care was received in another location, please specify	notes
77	hh1_antenatal_whynone<br><br>Show the field ONLY if:<br>[hh1_antenatal_yn] = '0'	What was the main reason why you did not see anyone for antenatal care?<br>*Select all that apply*	checkbox<br>1 hh1_antenatal_whynone___1 Clinic too far<br>2 hh1_antenatal_whynone___2 Had no money<br>3 hh1_antenatal_whynone___3 Had no time<br>4 hh1_antenatal_whynone___4 Not aware had to attend<br>5 hh1_antenatal_whynone___5 Did not want to attend<br>99 hh1_antenatal_whynone___99 Other<br>98 hh1_antenatal_whynone___98 Don't know
78	hh1_anc_whynone_other<br><br>Show the field ONLY if:<br>[hh1_antenatal_whynone(99)] = '1'	If another reason, please specify:	notes
79	hh1_iptp_yn	4.C.9. During this most recent pregnancy, did you take any drugs to keep you from getting malaria?	radio<br>1 Yes<br>0 No<br>98 Don't know
80	hh1_iptp_whynone<br><br>Show the field ONLY if:<br>[hh1_iptp_yn] = '0'	What was the main reason why you did not take any drugs to keep you from getting malaria during this pregnancy?	radio<br>1 Clinic too far<br>2 Had no money<br>3 Side effects<br>4 Not aware had to take any<br>5 Did not want to take<br>99 Other<br>98 Don't know
81	hh1_iptp_whynone_other<br><br>Show the field ONLY if:<br>[hh1_iptp_whynone] = '99'	If another reason, please specify	notes
82	hh1_iptp_drug	4.C.10. What drugs did you take?RECORD ALL MENTIONED<br>*Select all that apply*	checkbox<br>1 hh1_iptp_drug___1 SP/Fansidar<br>2 hh1_iptp_drug___2 Chloroquine<br>99 hh1_iptp_drug___99 Other<br>98 hh1_iptp_drug___98 Don't know
83	hh1_iptp_spfreq<br><br>Show the field ONLY if:<br>[hh1_iptp_drug(1)] = '1'	How many times did you take SP/Fansidar during this pregnancy?	text (integer, Min: 0)
84	hh1_iptp_spfreq_whyonce	Why did you take (SP/Fansidar) only one time during this pregnancy?	radio<br>1 Clinic too far

	Show the field ONLY if:<br>[hh1_iptp_spfreq] <2		2 Had no money<br>3 Side effects<br>4 Not aware had to take more<br>5 Did not want to take<br>99 Other<br>98 Don't know
85	h1_iptp_spfreq_whyonce_other<br>Show the field ONLY if:<br>[hh1_iptp_spfreq_whyonce] = '99'	If other reason, please specify	notes
86	hh1_sp_where<br>Show the field ONLY if:<br>[hh1_iptp_drug(1)] = '1'	Did you get the (SP/Fansidar) during any antenatal care visit, during another visit to a health facility or from another source?	radio<br>1 Antenatal visit<br>2 Another facility visit<br>3 Other source
87	hh1_ocp_yn	4.C.11. Are you currently taking any form of birth control?	yesno<br>1 Yes<br>0 No
88	hh1_ocp_spec<br>Show the field ONLY if:<br>[hh1_ocp_yn] = '1'	If you are using some form of birth control, what method?	radio<br>0 Oral Contraceptive Pills (OCP)<br>1 Injectable Contraception<br>2 Intrauterine Device (IUD)<br>3 Implantable Device<br>99 Other
89	hh1_child_name	Section Header: *Section 4.D. Demographic Information - Participating Child*<br>4.D.1. What is the child's name?	text, Required, Identifier
90	hh1_child_sex	4.D.2. What is the child's sex	radio, Required<br>0 Female<br>1 Male
91	hh1_child_dob	4.D.3. What is the child's date of birth?	text (date_dmy, Min: 2020-01-01, Max: 2023-12-31), Required, Identifier
92	hh1_child_birth	4.D.4. Where was the child born?	radio<br>0 Public health centre<br>1 Public hospital<br>2 Private facility (Health Centre or Hospital)<br>3 Home<br>99 Other location
93	hh1_child_birth_other<br>Show the field ONLY if:<br>[hh1_child_birth] = '99'	If the child was born at another location, please specify	notes
94	hh1_child_birth_home<br>Show the field ONLY if:<br>[hh1_child_birth] = '3'	If the child was born at home, who else was present?	checkbox<br>0 hh1_child_birth_home___0 Family members<br>1 hh1_child_birth_home___1 Traditional birth attendant<br>2 hh1_child_birth_home___2 Midwife

			3 hh1_child_birth_home___3 Nurse<br>4 hh1_child_birth_home___4 Clinical Officer<br>5 hh1_child_birth_home___5 Doctor<br>98 hh1_child_birth_home___98 Don't remember<br><br>Field Annotation: @NONEOFTHEABOVE=98
95	hh1_child_deliv_type	4.D.5. How was the child born?	radio<br>0 Vaginal delivery<br>1 Assisted delivery (e.g., suction, forceps)<br>2 Caesarean section
96	hh1_child_premature	4.D.6. Was the child born premature (before 37 weeks)?	radio<br>0 No<br>1 Yes<br>98 Don't know
97	hh1_child_hosp	4.D.7. Other than for delivery, has (NAME OF CHILD) had to stay overnight in a hospital or health centre?	yesno<br>1 Yes<br>0 No
98	hh1_child_hosp_why<br><br>Show the field ONLY if:<br>[hh1_child_hosp] = '1'	What was the reason you had to stay in the hospital or health centre?If multiple events, please describe the most recent reason.	radio<br>1 Malaria<br>2 Fever, not malaria<br>3 Diarrhea<br>4 Breathing, cough, or pneumonia<br>5 Injury<br>99 Other
99	hh1_child_hosp_other<br><br>Show the field ONLY if:<br>[hh1_child_hosp_why] = '99'	If another reason for hospitalization, please specify:	notes
100	household_visit_demographics_complete	Section Header: *Form Status*<br>Complete?	dropdown<br>0 Incomplete<br>1 Unverified<br>2 Complete
**Instrument: 5. Household Visit - HH Characteristics (household_visit_hh_characteristics)**			
101	hh2_subcounty	Section Header: *Section 5.A. Household Location*<br>5.A.1. What is the Sub-County of Residence?	radio, Required, Identifier<br>0 Bugoye<br>1 Maliba<br>99 Other
102	hh2_village_bug<br><br>Show the field ONLY if:<br>[hh2_subcounty] = '0'	If a resident of Bugoye sub-county, what is your village of residence?	dropdown, Required, Identifier<br>1 Bugoye<br>2 Bunyangoni<br>3 Ibanda II<br>4 Ihani<br>5 Kanyaminigo<br>6 Katooke<br>7 Kasanzi

			8 Kikokera<br>9 Kirongo<br>10 Muramba I<br>11 Ndugutu East<br>12 Ndugutu West<br>13 Rwakingi IA<br>14 Rwakingi IB
103	hh2_village_malib<br>Show the field ONLY if:<br>[hh2_subcounty] = '1'	If a resident of Maliba sub-county, what is your village of residence?	dropdown, Required, Identifier<br>1 Bulyandeke<br>2 Bwizibwera<br>3 Izinga<br>4 Kaghando<br>16 Kakindo<br>5 Katebe II<br>6 Kibota<br>7 Kihyo<br>8 Kiruli 1A<br>9 Kiruli 1B<br>10 Kyandali<br>11 Kyanjima<br>12 Maliba I<br>13 Maliba II<br>14 Nyabisusi<br>15 Nyarukungu
104	hh2_subcounty_other<br>Show the field ONLY if:<br>[hh2_subcounty] = '99'	If other sub-county selected, please list name of sub-county and village and explain why participant being enrolled.	notes, Identifier
105	hh2_lat1	5.A.2. Household latitude (4 digits after decimal)<br>*Example 0.3060*	text (number, Min: 0.2, Max: 0.4), Required, Identifier
106	hh2_lat2	5.A.3. Confirm household latitude	text (number, Min: 0.2, Max: 0.4), Required, Identifier
107	hh2_lat_check<br>Show the field ONLY if:<br>[hh2_lat1]<>[hh2_lat2] and [hh2_lat1] <> "" and [hh2_lat2] < > ""	LATITUDE DOES NOT MATCH	descriptive
108	hh2_long1	5.A.4. Household longitude (4 digits after decimal)<br>*Example = 30.0768*	text (number, Min: 29, Max: 32), Required, Identifier
109	hh2_long2	5.A.5. Confirm household longitude	text (number, Min: 29, Max: 32), Required, Identifier
110	hh2_long_check<br>Show the field ONLY if:<br>[hh2_long1] <> [hh2_long2] and [hh2_long1]<>"" and [hh2_long2]<>""	LONGITUDE DOES NOT MATCH	descriptive
111	hh2_hhmembers	Section Header: *Section 5.B. Household Residents*<br>5.B.1. How many people - excluding you and your child - stay in this house at lest three nights per week?	text (integer, Min: 0, Max: 15), Required
112	hh2_hhmember_order	Please start from the oldest person in the household Be sure	descriptive

	Show the field ONLY if: [hh2_hhmembers] >= 1	not to include information about the participating mother and child	
113	hh2_hhmem1_age<br>Show the field ONLY if: [hh2_hhmembers] >= 1	What is Household Member #1's age?<br>*Years*	text (integer, Min: 1, Max: 99)
114	hh2_hhmem1_sex<br>Show the field ONLY if: [hh2_hhmembers] >= 1	What is Household Member #1's sex?	radio<br>0 Female<br>1 Male
115	hh2_hhmem1_relate<br>Show the field ONLY if: [hh2_hhmembers] >= 1	What is Household Member #1's relationship to you?	radio<br>0 Spouse<br>1 Parent<br>2 Child<br>3 Other family member (aunt, uncle, etc.)<br>4 Non-family member
116	hh2_hhmem2_age<br>Show the field ONLY if: [hh2_hhmembers] >= 2	What is Household Member #2's age?<br>*Years*	text (integer, Min: 1, Max: 99)
117	hh2_hhmem2_sex<br>Show the field ONLY if: [hh2_hhmembers] >= 2	What is Household Member #2's sex?	radio<br>0 Female<br>1 Male
118	hh2_hhmem2_relate<br>Show the field ONLY if: [hh2_hhmembers] >= 2	What is Household Member #2's relationship to you?	radio<br>0 Spouse<br>1 Parent<br>2 Child<br>3 Other family member (aunt, uncle, etc.)<br>4 Non-family member
119	hh2_hhmem3_age<br>Show the field ONLY if: [hh2_hhmembers] >= 3	What is Household Member #3's age?<br>*Years*	text (integer, Min: 1, Max: 99)
120	hh2_hhmem3_sex<br>Show the field ONLY if: [hh2_hhmembers] >= 3	What is Household Member #3's sex?	radio<br>0 Female<br>1 Male
121	hh2_hhmem3_relate<br>Show the field ONLY if: [hh2_hhmembers] >= 3	What is Household Member #3's relationship to you?	radio<br>0 Spouse<br>1 Parent<br>2 Child<br>3 Other family member (aunt, uncle, etc.)<br>4 Non-family member
122	hh2_hhmem4_age<br>Show the field ONLY if: [hh2_hhmembers] >= 4	What is Household Member #4's age?<br>*Years*	text (integer, Min: 1, Max: 99)
123	hh2_hhmem4_sex<br>Show the field ONLY if: [hh2_hhmembers] >= 4	What is Household Member #4's sex?	radio<br>0 Female<br>1 Male

124	hh2_hhmem4_relate<br>Show the field ONLY if:<br>[hh2_hhmembers] >= 4	What is Household Member #4's relationship to you?	radio<br>0 Spouse<br>1 Parent<br>2 Child<br>3 Other family member (aunt, uncle, etc.)<br>4 Non-family member
125	hh2_hhmem5_age<br>Show the field ONLY if:<br>[hh2_hhmembers] >= 5	What is Household Member #5's age?<br>*Years*	text (integer, Min: 1, Max: 99)
126	hh2_hhmem5_sex<br>Show the field ONLY if:<br>[hh2_hhmembers] >= 5	What is Household Member #5's sex?	radio<br>0 Female<br>1 Male
127	hh2_hhmem5_relate<br>Show the field ONLY if:<br>[hh2_hhmembers] >= 5	What is Household Member #5's relationship to you?	radio<br>0 Spouse<br>1 Parent<br>2 Child<br>3 Other family member (aunt, uncle, etc.)<br>4 Non-family member
128	hh2_hhmem6_age<br>Show the field ONLY if:<br>[hh2_hhmembers] >= 6	What is Household Member #6's age?<br>*Years*	text (integer, Min: 1, Max: 99)
129	hh2_hhmem6_sex<br>Show the field ONLY if:<br>[hh2_hhmembers] >= 6	What is Household Member #6's sex?	radio<br>0 Female<br>1 Male
130	hh2_hhmem6_relate<br>Show the field ONLY if:<br>[hh2_hhmembers] >= 6	What is Household Member #6's relationship to you?	radio<br>0 Spouse<br>1 Parent<br>2 Child<br>3 Other family member (aunt, uncle, etc.)<br>4 Non-family member
131	hh2_hhmem7_age<br>Show the field ONLY if:<br>[hh2_hhmembers] >= 7	What is Household Member #7's age?<br>*Years*	text (integer, Min: 1, Max: 99)
132	hh2_hhmem7_sex<br>Show the field ONLY if:<br>[hh2_hhmembers] >= 7	What is Household Member #7's sex?	radio<br>0 Female<br>1 Male
133	hh2_hhmem7_relate<br>Show the field ONLY if:<br>[hh2_hhmembers] >= 7	What is Household Member #7's relationship to you?	radio<br>0 Spouse<br>1 Parent<br>2 Child<br>3 Other family member (aunt, uncle, etc.)<br>4 Non-family member
134	hh2_hhmem8_age<br>Show the field ONLY if:<br>[hh2_hhmembers] >= 8	What is Household Member #8's age?<br>*Years*	text (integer, Min: 1, Max: 99)

135	hh2_hhmem8_sex<br><br>Show the field ONLY if:<br>[hh2_hhmembers] >= 8	What is Household Member #8's sex?	radio<br>0 Female<br>1 Male
136	hh2_hhmem8_relate<br><br>Show the field ONLY if:<br>[hh2_hhmembers] >= 8	What is Household Member #8's relationship to you?	radio<br>0 Spouse<br>1 Parent<br>2 Child<br>3 Other family member (aunt, uncle, etc.)<br>4 Non-family member
137	hh2_hhmem9_age<br><br>Show the field ONLY if:<br>[hh2_hhmembers] >= 9	What is Household Member #9's age?<br>*Years*	text (integer, Min: 1, Max: 99)
138	hh2_hhmem9_sex<br><br>Show the field ONLY if:<br>[hh2_hhmembers] >= 9	What is Household Member #9's sex?	radio<br>0 Female<br>1 Male
139	hh2_hhmem9_relate<br><br>Show the field ONLY if:<br>[hh2_hhmembers] >= 9	What is Household Member #9's relationship to you?	radio<br>0 Spouse<br>1 Parent<br>2 Child<br>3 Other family member (aunt, uncle, etc.)<br>4 Non-family member
140	hh2_hhmem10_age<br><br>Show the field ONLY if:<br>[hh2_hhmembers] >= 10	What is Household Member #10's age?<br>*Years*	text (integer, Min: 1, Max: 99)
141	hh2_hhmem10_sex<br><br>Show the field ONLY if:<br>[hh2_hhmembers] >= 10	What is Household Member #10's sex?	radio<br>0 Female<br>1 Male
142	hh2_hhmem10_relate<br><br>Show the field ONLY if:<br>[hh2_hhmembers] >= 10	What is Household Member #10's relationship to you?	radio<br>0 Spouse<br>1 Parent<br>2 Child<br>3 Other family member (aunt, uncle, etc.)<br>4 Non-family member
143	hh2_hhmem_tenplus<br><br>Show the field ONLY if:<br>[hh2_hhmembers] > 10	If there are more than 10 additional household members, please list their age, sex, and relationship to the participant in this section	notes
144	hh2_floor_material	Section Header: *5.C. Household Construction*<br>5.C.1. Observe main material of the floor of the dwelling.<br><br>Record observation.	checkbox<br>1 hh2_floor_material___1 Earth/sand<br>2 hh2_floor_material___2 Dung<br>3 hh2_floor_material___3 Wood planks<br>4 hh2_floor_material___4 Palm/Bamboo<br>5 hh2_floor_material___5 Parquet or polished wood<br>6 hh2_floor_material___6 Concrete

			7 hh2_floor_material___7 Ceramic tiles<br>8 hh2_floor_material___8 Cement screed<br>9 hh2_floor_material___9 Carpet<br>10 hh2_floor_material___10 Stones<br>11 hh2_floor_material___11 Bricks<br>12 hh2_floor_material___12 Other
145	hh2_roof_material	5.C.2. Observe main material of the roof of the dwelling.<br><br>Record observation.	checkbox<br>1 hh2_roof_material___1 No roof<br>2 hh2_roof_material___2 Thatch/palm leaf<br>3 hh2_roof_material___3 Mud<br>4 hh2_roof_material___4 Rustic mat<br>5 hh2_roof_material___5 Tins<br>6 hh2_roof_material___6 Wood planks<br>7 hh2_roof_material___7 Cardboard<br>8 hh2_roof_material___8 Tarpaulin<br>9 hh2_roof_material___9 Iron sheets<br>10 hh2_roof_material___10 Wood<br>11 hh2_roof_material___11 Asbestos<br>12 hh2_roof_material___12 Tiles<br>13 hh2_roof_material___13 Concrete<br>14 hh2_roof_material___14 Roofing Shingles<br>15 hh2_roof_material___15 Other
146	hh2_exteriorwalls_material	5.C.3. Observe main material of the exterior walls of the dwelling. Record observation.	checkbox<br>1 hh2_exteriorwalls_material___1 No walls<br>2 hh2_exteriorwalls_material___2 Thatched/straw<br>3 hh2_exteriorwalls_material___3 Dirt<br>4 hh2_exteriorwalls_material___4 Poles with mud<br>5 hh2_exteriorwalls_material___5 Stone with mud<br>6 hh2_exteriorwalls_material___6 Unburnt bricks with mud<br>7 hh2_exteriorwalls_material___7 Plywood<br>8 hh2_exteriorwalls_material___8 Cardboard<br>9 hh2_exteriorwalls_material___9 Reused wood<br>10 hh2_exteriorwalls_material___10 Unburnt bricks with plaster<br>11 hh2_exteriorwalls_material___11 Burnt bricks with mud<br>12 hh2_exteriorwalls_material___12 Cement<br>13 hh2_exteriorwalls_material___13 Stone with lime/cement<br>14 hh2_exteriorwalls_material___14 Burnt bricks with cement<br>15 hh2_exteriorwalls_material___15 Cement blocks<br>16 hh2_exteriorwalls_material___16 Unburnt bricks with cement

			17 hh2_exteriorwalls_material___17 Wood planks/shingles<br>18 hh2_exteriorwalls_material___18 Other
147	hh2_water_drinking_source	5.C.4. What is the main source of drinking water for members of your household?	radio<br>1 Piped into dwelling<br>2 Piped to yard/Plot<br>3 Piped to neighbor<br>4 Public tap/Standpipe<br>5 Tube well or borehole<br>6 Protected well<br>7 Unprotected well<br>8 Protected spring<br>9 Unprotected spring<br>10 Rainwater<br>11 Tanker truck<br>12 Bicycle with jerrycans<br>13 Surface water (River/Dam/Lake/Pond/Stream/Canal/Irrigation channel)<br>14 Bottled water<br>15 Sachet water<br>16 Other
148	hh2_water_other_source	5.C.5. What is the main source of water used by your household for other purposes such as cooking and handwashing?	radio<br>1 Piped into dwelling<br>2 Piped to yard/Plot<br>3 Piped to neighbor<br>4 Public tap/Standpipe<br>5 Tube well or borehole<br>6 Protected well<br>7 Unprotected well<br>8 Protected spring<br>9 Unprotected spring<br>10 Rainwater<br>11 Tanker truck<br>12 Bicycle with jerrycans<br>13 Surface water (River/Dam/Lake/Pond/Stream/Canal/Irrigation channel)<br>14 Other
149	hh2_water_source_where	5.C.6. Where is that water source located?	radio<br>1 In own dwelling<br>2 In own yard/plot<br>3 Elsewhere
150	hh2_water_time	5.C.7. How long does it take to go there, get water, and come back?	radio

			1 Minutes selected<br>99 Don't know
151	hh2_water_time_min<br>Show the field ONLY if:<br>[hh2_water_time] = '1'	Minutes:	text (integer, Min: 1, Max: 180)
152	hh2_water_trt_yn	5.C.8. Do you do anything to the water to make it safer to drink?	radio<br>1 Yes<br>0 No<br>99 Don't know
153	hh2_water_trt<br>Show the field ONLY if:<br>[hh2_water_trt_yn]='1'	What do you usually do to make the water safer to drink?	checkbox<br>1 hh2_water_trt___1 Boil<br>2 hh2_water_trt___2 Add bleach/chlorine<br>3 hh2_water_trt___3 Strain through a cloth<br>4 hh2_water_trt___4 Use water filter (Ceramic/Sand/Composite/Etc)<br>5 hh2_water_trt___5 Solar disinfection<br>6 hh2_water_trt___6 Let it stand and settle<br>7 hh2_water_trt___7 Other<br>8 hh2_water_trt___8 Don't know
154	hh2_water_trt_other<br>Show the field ONLY if:<br>[hh2_water_trt(7)] = '1'	If other treatment selected, please specify.	notes
155	hh2_toilet_type	5.C.9. What kind of toilet facility do members of your household usually use?	radio<br>1 Flush to piped sewer system<br>2 Flush to septic tank<br>3 Flush to pit latrine<br>4 Flush to somewhere else<br>5 Flush (don't know where)<br>6 Ventilated improved pit latrine<br>7 Pit latrine with slab<br>8 Pit latrine without slab/Open pit<br>9 Composting toilet/Ecosan<br>10 Bucket toilet<br>11 Hanging toilet/Hanging latrine<br>12 No facility/Bush/Field<br>13 Other
156	hh2_toilet_type_other<br>Show the field ONLY if:<br>[hh2_toilet_type] = '13'	If other toilet type selected, please specify.	notes
157	hh2_toilet_share_yn	5.C.10. Do you share this toilet facility with other households?	yesno<br>1 Yes<br>0 No
158	hh2_toilet_share_number<br>Show the field ONLY if:	How many households share this toilet?	text (integer, Min: 1, Max: 100)

	[hh2_toilet_share_number] = ' 1'		
159	hh2_toilet_where	5.C.11. Where is this toilet facility located?	radio<br>1 In own dwelling<br>2 In own yard/plot<br>3 Elsewhere
160	hh2_fuel_type	5.C.12. What type of fuel does your household mainly use for cooking?	radio<br>1 Electricity<br>2 LPG/Cylinder Gas<br>3 Biogas<br>4 Kerosene<br>5 Charcoal<br>6 Wood<br>7 Straws/Shrubs/Grass<br>8 Agricultural crop<br>9 Animal dung<br>10 No food cooked in household<br>11 Other
161	hh2_cooking_where	5.C.13. Is the cooking usually done in the house, in a separate building, or outdoors?	radio<br>1 In the house<br>2 In a separate building<br>3 Outdoors
162	hh2_kitchen_separate_yn	5.C.14. Do you have a separate room which is used as a kitchen?	yesno<br>1 Yes<br>0 No
163	hh2_animals_yn	Section Header: *5.D. Ownership of Property*<br>5.D.1. Does this household own any livestock, herds, other farm animals, or poultry?	yesno<br>1 Yes<br>0 No
164	hh2_cattle_number<br>Show the field ONLY if:<br>[hh2_animals_yn] = '1'	How many of the following animals does this household own? a) Local cattle?	text (integer)
165	hh2_horse_number<br>Show the field ONLY if:<br>[hh2_animals_yn] ='1'	b) Horses, donkeys, or mules?	text (integer)
166	hh2_goat_number<br>Show the field ONLY if:<br>[hh2_animals_yn] ='1'	c) Goats?	text (integer)
167	hh2_sheep_number<br>Show the field ONLY if:<br>[hh2_animals_yn] ='1'	d) Sheep?	text (integer)
168	hh2_chicken_number<br>Show the field ONLY if:<br>[hh2_animals_yn] ='1'	e) Chickens or other poultry (e.g., ducks, geese, fowl)?	text (integer)
169	hh2_pig_number<br>Show the field ONLY if:<br>[hh2_animals_yn] ='1'	f) Pigs?	text (integer)

170	hh2_animals_inside_yn<br>Show the field ONLY if:<br>[hh2_animals_yn] ='1'	Are there any animals that sleep in the house where people sleep?	yesno<br>1 Yes<br>0 No
171	hh2_land_agriculture_yn	5.D.2. Does any member of this household own any agricultural land?	yesno<br>1 Yes<br>0 No
172	hh2_land_agriculture_acres<br>Show the field ONLY if:<br>[hh2_land_agriculture_acres] = '1'	Approximately, how many acres:	text (integer)
173	electricity_yn	Section Header: *5.D.3. Does your household have:*<br>Electricity?	radio (Matrix)<br>1 Yes<br>2 No
174	radio_yn	A radio?	radio (Matrix)<br>1 Yes<br>2 No
175	television_yn	A television?	radio (Matrix)<br>1 Yes<br>2 No
176	nonmobile_yn	A non-mobile telephone?	radio (Matrix)<br>1 Yes<br>2 No
177	computer_yn	A computer?	radio (Matrix)<br>1 Yes<br>2 No
178	fridge_yn	A refrigerator?	radio (Matrix)<br>1 Yes<br>2 No
179	musicplayer_yn	A cassette/CD/DVD player?	radio (Matrix)<br>1 Yes<br>2 No
180	table_yn	A table?	radio (Matrix)<br>1 Yes<br>2 No
181	chair_yn	A chair?	radio (Matrix)<br>1 Yes<br>2 No
182	sofa_yn	A sofa set?	radio (Matrix)<br>1 Yes<br>2 No
183	bed_yn	A bed?	radio (Matrix)<br>1 Yes

			2 No
184	cupboard_yn	A cupboard?	radio (Matrix)<br>1 Yes<br>2 No
185	clock_yn	A clock?	radio (Matrix)<br>1 Yes<br>2 No
186	watch_yn	Section Header: *5.D.4. Does any member of this household own:*<br>A watch?	radio (Matrix)<br>1 Yes<br>2 No
187	mobilephone_yn	A mobile phone?	radio (Matrix)<br>1 Yes<br>2 No
188	bike_yn	A bicycle?	radio (Matrix)<br>1 Yes<br>2 No
189	motorcycle_yn	A motorcycle/scooter?	radio (Matrix)<br>1 Yes<br>2 No
190	cart_yn	An animal-drawn cart?	radio (Matrix)<br>1 Yes<br>2 No
191	car_yn	A car/truck?	radio (Matrix)<br>1 Yes<br>2 No
192	motorboat_yn	A boat with a motor?	radio (Matrix)<br>1 Yes<br>2 No
193	boat_yn	A boat without a motor?	radio (Matrix)<br>1 Yes<br>2 No
194	hh2_bank_yn	5.D.5. Does any member of this household have a bank account, mobile money account, or account with an agent?	yesno<br>1 Yes<br>0 No
195	household_visit_hh_characteristics_complete	Section Header: *Form Status*<br>Complete?	dropdown<br>0 Incomplete<br>1 Unverified<br>2 Complete
**Instrument: 6. Household Visit - Malaria And Care Seeking (household_visit_malaria_and_care_seeking)**			
196	healthcare_where	Section Header: *6.A. General Care Seeking*<br>6.A.1 Where do you and your family mainly go for health care?	radio<br>1 Government hospital<br>2 Government health center<br>3 Family planning clinic

			4 Mobile clinic<br>5 Other public sector<br>6 Private hospital/clinic<br>7 Private doctor<br>8 Mobile private clinic<br>9 Pharmacy/drug shop<br>10 Other private medical center<br>11 Other<br>12 Don't know
197	healthcare_where_other<br><br>Show the field ONLY if:<br>[healthcare_where] = '11'	If another facility, please write the name of the place.	notes
198	healthcare_travel	6.A.2. How do you usually get there?	radio<br>0 Walk<br>1 Motorcycle<br>2 Car or Taxi<br>3 Bus<br>99 Other
199	healthcare_travel_time	6.A.3. Approximately how long does it take you to get to clinic?<br>*Minutes*	text (integer)
200	healthcare_pay_yn	6.A.4. Do you pay any money for the services offered?	radio<br>1 Yes (official fees)<br>2 Yes (token of thanks)<br>3 No<br>4 Don't know
201	healthcare_pay_how<br><br>Show the field ONLY if:<br>[healthcare_pay_yn] = '1' or [healthcare_pay_yn] = '2'	How do you make the payment?	radio<br>1 Directly out of pocket<br>2 Community-based initiative/savings<br>3 Health insurance through employer<br>4 Social security<br>5 Other privately purchased commercial health insurance<br>6 Other
202	healthcare_pay_how_else<br><br>Show the field ONLY if:<br>[healthcare_pay_how] = '6'	If another methods of payment, please specify:	notes
203	feverdrug_when	Section Header: *6.B. Care Seeking for Fever*<br>6.B.1. I would like to ask you a few questions about fever in children. When a child is sick with fever, how long after the fever begins should the child be taken for treatment?	radio<br>1 Same day<br>2 Next day<br>3 Two days after onset of fever<br>4 Three or more days after onset of fever<br>5 Fever is normal in children, no treatment necessary<br>6 Depends on how serious the fever is

			7 Other<br>8 Don't know
204	feverdrug_when_other<br>Show the field ONLY if:<br>[feverdrug_when] = '7'	If other is selected, please specify:	notes
205	mrb_fever_yn	6.B.2. Has (NAME OF CHILD) been ill with a fever at any time in the last 2 weeks?	radio<br>1 Yes<br>0 No<br>99 Don't Know
206	mrb_blooddraw_yn<br>Show the field ONLY if:<br>[mrb_fever_yn] = '1'	At any time during the illness, did (NAME OF CHILD) have blood taken from his/her finger or heel for testing?	radio<br>1 Yes<br>2 No<br>3 Don't know
207	mrb_adviceortrt_yn<br>Show the field ONLY if:<br>[mrb_fever_yn]='1'	Did you seek advice or treatment for the illness from any source?	radio<br>1 Yes<br>0 No
208	mrb_adviceortrt_whynone<br>Show the field ONLY if:<br>[mrb_adviceortrt_yn] = '0'	If no, what was the main reason you have you not sought advice or treatment from any source?	radio<br>1 Child just fell ill<br>2 Child not very ill<br>3 Clinic too far<br>4 Have no money<br>5 Waiting for child's father<br>6 Don't know what to do<br>7 Already had medicine at home<br>8 Other
209	mrb_adviceortrt_whynone_other<br>Show the field ONLY if:<br>[mrb_adviceortrt_yn] = '8'	If another reason, please specify.	notes
210	mrb_adviceortrt_where<br>Show the field ONLY if:<br>[mrb_adviceortrt_yn] = '1'	Where did you seek advice or treatment? Anywhere else?<br>*Please check all that apply*	checkbox<br>1 mrb_adviceortrt_where___1 Government hospital<br>2 mrb_adviceortrt_where___2 Government health center<br>3 mrb_adviceortrt_where___3 Government health post<br>4 mrb_adviceortrt_where___4 Mobile clinic<br>5 mrb_adviceortrt_where___5 Community health worker (VHT)<br>6 mrb_adviceortrt_where___6 Other public sector<br>7 mrb_adviceortrt_where___7 Public sector (PNFP) hospital<br>8 mrb_adviceortrt_where___8 Public sector (PNFP) health center

			9 mrb_adviceortrt_where___9 Private hospital/clinic<br>10 mrb_adviceortrt_where___10 Pharmacy<br>11 mrb_adviceortrt_where___11 Private mobile clinic<br>12 mrb_adviceortrt_where___12 Fieldworker<br>13 mrb_adviceortrt_where___13 Other private medical sector<br>14 mrb_adviceortrt_where___14 Shop<br>15 mrb_adviceortrt_where___15 Traditional practitioner<br>16 mrb_adviceortrt_where___16 Market<br>17 mrb_adviceortrt_where___17 Other
211	mrb_adviceortrt_where_other<br>Show the field ONLY if:<br>[mrb_adviceortrt_yn]='1'	If another facility, please write the name of the place.	notes
212	mrb_drugstaken_yn	At any time during the illness, did (NAME OF CHILD) take any drugs for the illness?	radio<br>1 Yes<br>2 No<br>3 Don't know
213	malaria_cause	Section Header: *6.C. Malaria Knowledge*<br>6.C.1. In your opinion, what causes malaria?	checkbox<br>1 malaria_cause___1 Mosquito bites<br>2 malaria_cause___2 Parasite<br>3 malaria_cause___3 Eating maize<br>4 malaria_cause___4 Eating mangoes<br>5 malaria_cause___5 Eating dirty food<br>6 malaria_cause___6 Drinking unboiled water<br>7 malaria_cause___7 Getting soaked with rain<br>8 malaria_cause___8 Cold/changing weather<br>9 malaria_cause___9 Witchcraft<br>10 malaria_cause___10 Contact with infected person<br>11 malaria_cause___11 Germ<br>12 malaria_cause___12 Other<br>13 malaria_cause___13 Don't know
214	malaria_cause_other<br>Show the field ONLY if:<br>[malaria_cause(12)] = '1'	If other selected, please specify:	notes
215	malaria_prevent_yn	6.C.2. Are there ways to avoid getting malaria?	yesno<br>1 Yes<br>0 No
216	malaria_prevent_how<br>Show the field ONLY if:<br>[malaria_prevent_yn] = '1'	What are the ways to avoid getting malaria?<br>*Select all that apply*	checkbox<br>1 malaria_prevent_how___1 Sleep under mosquito net<br>2 malaria_prevent_how___2 Sleep under an insecticide treated

			net<br>3 malaria_prevent_how___3 Taking preventive medication<br>4 malaria_prevent_how___4 Use mosquito repellant<br>5 malaria_prevent_how___5 Spraying house with insecticide<br>6 malaria_prevent_how___6 Using mosquito coils<br>7 malaria_prevent_how___7 Destroy mosquito breeding sites<br>8 malaria_prevent_how___8 Other<br>9 malaria_prevent_how___9 Don't know
217	malaria_prevent_how_other<br>Show the field ONLY if:<br>[malaria_prevent_how(8)] = '1'	If other selected, please specify:	notes
218	malaria_drug_pregnancy	6.C.3. What medicine may be given to a pregnant woman to help her avoid getting malaria?<br>*Select all that apply*	checkbox<br>1 malaria_drug_pregnancy___1 SP/Fansidar<br>2 malaria_drug_pregnancy___2 Chloroquine<br>3 malaria_drug_pregnancy___3 Cholorquine w/Fansidar<br>4 malaria_drug_pregnancy___4 Coartem/Act<br>5 malaria_drug_pregnancy___5 Other<br>6 malaria_drug_pregnancy___6 Don't know
219	malaria_drug_pregnancy_other<br>Show the field ONLY if:<br>[malaria_drug_pregnancy(5)] = '1'	If other selected, please specify:	notes
220	spray_walls_yn	Section Header: *6.D. Malaria Prevention*<br>6.D.1. At any time in the past 6 months, has anyone come into your dwelling to spray the interior walls against mosquitoes?	radio<br>1 Yes<br>0 No<br>2 Don't know
221	spray_who<br>Show the field ONLY if:<br>[spray_walls_yn] = '1'	Who sprayed the dwelling?	radio<br>1 Government worker/program<br>2 Private company<br>3 Nongovernmental organization (NGO)<br>4 Other<br>5 Don't know
222	spray_who_other<br>Show the field ONLY if:<br>[spray_who] = '4'	If other selected, please specify:	notes
223	spray_pay_yn<br>Show the field ONLY if:<br>[spray_walls_yn] ='1'	Did you pay for your dwelling to be sprayed?	radio<br>1 Yes<br>2 No<br>3 Don't know

224	net_yn	6.D.2. Does your household have any mosquito nets?	yesno<br>1 Yes<br>0 No
225	net_number<br>Show the field ONLY if:<br>[net_yn] = '1'	How many mosquito nets does your household have?<br><br>Number of nets:	text (integer, Min: 1, Max: 10)
226	net_1_seen<br>Show the field ONLY if:<br>[net_number] >= 00	Net #1:	radio<br>1 Observed<br>0 Not observed
227	net_1_when<br>Show the field ONLY if:<br>[net_1_seen] = '1'	How many months ago did your household get the mosquito net?	radio<br>1 'Months ago' selected<br>2 More than 36 months ago<br>3 Not sure
228	net_1_months<br>Show the field ONLY if:<br>[net_1_when] = '1'	Months ago:	text (integer, Min: 1, Max: 36)
229	net_1_brand<br>Show the field ONLY if:<br>[net_1_seen] = '1'	Observe or ask brand/type of mosquito net.	radio<br>1 Permanent LLIN<br>2 Duranet LLIN<br>3 Interceptor LLIN<br>4 Olyset LLIN<br>5 Dawnet LLIN<br>6 Iconlife LLIN<br>7 Yorkool LLIN<br>8 DK brand LLIN<br>9 Govt brand LLIN<br>10 Other LLIN<br>11 Other brand<br>12 DK brand
230	net_1_brand_other<br>Show the field ONLY if:<br>[net_1_brand] = '10'	If other selected, please specify:	notes
231	net_1_how<br>Show the field ONLY if:<br>[net_number]>= '1'	Did you get the net through a mass distribution, during an antenatal care visit, or during an immunization visit?	radio<br>1 Yes (mass distribution)<br>2 Yes (ANC)<br>3 Yes (immunization visit)<br>4 No
232	net_1_where<br>Show the field ONLY if:<br>[net_number] >='1'	Where did you get the net?	radio<br>1 Govt. hospital<br>2 Govt. health facility<br>3 Hospital (PNFP/NGO)<br>4 Health facility (PNFP/NGO)<br>5 Private hospital/clinic<br>6 Pharmacy

			7 Shop/market<br>8 Hawker<br>9 CHW<br>10 Religious institution<br>11 Other<br>12 Don't know
233	net_1_sleep_yn<br>Show the field ONLY if:<br>[net_number]>='1'	Did anyone sleep under this mosquito net last night?	radio<br>1 Yes<br>0 No<br>99 Not sure
234	net_1_sleep_num<br>Show the field ONLY if:<br>[net_1_sleep_yn] = '1'	How many people slept under this mosquito net last night?	text (integer, Min: 1, Max: 10)
235	net_2_seen<br>Show the field ONLY if:<br>[net_number] >= 2	Net #2:	radio<br>1 Observed<br>0 Not observed
236	net_2_when<br>Show the field ONLY if:<br>[net_2_seen] = '1'	How many months ago did your household get the mosquito net?	radio<br>1 'Months ago' selected<br>2 More than 36 months ago<br>99 Not sure
237	net_2_months<br>Show the field ONLY if:<br>[net_2_when] = '1'	Months ago:	text (integer, Min: 1, Max: 36)
238	net_2_brand<br>Show the field ONLY if:<br>[net_2_seen] = '1'	Observe or ask brand/type of mosquito net.	radio<br>1 Permanent LLIN<br>2 Duranet LLIN<br>3 Interceptor LLIN<br>4 Olyset LLIN<br>5 Dawnet LLIN<br>6 Iconlife LLIN<br>7 Yorkool LLIN<br>8 DK brand LLIN<br>9 Govt brand LLIN<br>10 Other LLIN<br>11 Other brand<br>12 DK brand
239	net_2_brand_other<br>Show the field ONLY if:<br>[net_2_brand] = '10'	If other selected, please specify:	notes
240	net_2_how<br>Show the field ONLY if:<br>[net_number] >= 2	Did you get the net through a mass distribution, during an antenatal care visit, or during an immunization visit?	radio<br>1 Yes (mass distribution)<br>2 Yes (ANC)<br>3 Yes (immunization visit)<br>4 No

241	net_2_where<br>Show the field ONLY if:<br>[net_number] >= 2	Where did you get the net?	radio<br>1 Govt. hospital<br>2 Govt. health facility<br>3 Hospital (PNFP/NGO)<br>4 Health facility (PNFP/NGO)<br>5 Private hospital/clinic<br>6 Pharmacy<br>7 Shop/market<br>8 Hawker<br>9 CHW<br>10 Religious institution<br>11 Other<br>12 Don't know
242	net_2_sleep_yn<br>Show the field ONLY if:<br>[net_number] >= 2	Did anyone sleep under this mosquito net last night?	radio<br>1 Yes<br>2 No<br>3 Not sure
243	net_2_sleep_num<br>Show the field ONLY if:<br>[net_2_sleep_yn] = '1'	How many people slept under this mosquito net last night?	text (integer, Min: 1, Max: 10), Identifier
244	net_3_seen<br>Show the field ONLY if:<br>[net_number] >= 3	Net #3:	radio<br>1 Observed<br>0 Not observed
245	net_3_when<br>Show the field ONLY if:<br>[net_3_seen] = '1'	How many months ago did your household get the mosquito net?	radio<br>1 'Months ago' selected<br>2 More than 36 months ago<br>3 Not sure
246	net_3_months<br>Show the field ONLY if:<br>[net_3_when] = '1'	Months ago:	text (integer, Min: 1, Max: 36)
247	net_3_brand<br>Show the field ONLY if:<br>[net_3_seen] = '1'	Observe or ask brand/type of mosquito net.	radio<br>1 Permanent LLIN<br>2 Duranet LLIN<br>3 Interceptor LLIN<br>4 Olyset LLIN<br>5 Dawnet LLIN<br>6 Iconlife LLIN<br>7 Yorkool LLIN<br>8 DK brand LLIN<br>9 Govt brand LLIN<br>10 Other LLIN<br>11 Other brand<br>12 DK brand
248	net_3_brand_other	If other selected, please specify:	notes

	Show the field ONLY if: [net_3_brand] = '10'		
249	net_3_how<br>Show the field ONLY if: [net_number] >= 3	Did you get the net through a mass distribution, during an antenatal care visit, or during an immunization visit?	radio<br>1 Yes (mass distribution)<br>2 Yes (ANC)<br>3 Yes (immunization visit)<br>4 No
250	net_3_where<br>Show the field ONLY if: [net_number] >= 3	Where did you get the net?	radio<br>1 Govt. hospital<br>2 Govt. health facility<br>3 Hospital (PNFP/NGO)<br>4 Health facility (PNFP/NGO)<br>5 Private hospital/clinic<br>6 Pharmacy<br>7 Shop/market<br>8 Hawker<br>9 CHW<br>10 Religious institution<br>11 Other<br>12 Don't know
251	net_3_sleep_yn<br>Show the field ONLY if: [net_number] >= 3	Did anyone sleep under this mosquito net last night?	radio<br>1 Yes<br>0 No<br>99 Not sure
252	net_3_sleep_num<br>Show the field ONLY if: [net_3_sleep_yn] = '1'	How many people slept under this mosquito net last night?	text (integer, Min: 1, Max: 10), Identifier
253	net_4_seen<br>Show the field ONLY if: [net_number] >= 4	Net #4:	radio<br>1 Observed<br>0 Not observed
254	net_4_when<br>Show the field ONLY if: [net_4_seen] = '1'	How many months ago did your household get the mosquito net?	radio<br>1 'Months ago' selected<br>2 More than 36 months ago<br>3 Not sure
255	net_4_months<br>Show the field ONLY if: [net_4_when] = '1'	Months ago:	text (integer, Min: 1, Max: 36)
256	net_4_brand<br>Show the field ONLY if: [net_4_seen] = '1'	Observe or ask brand/type of mosquito net.	radio<br>1 Permanent LLIN<br>2 Duranet LLIN<br>3 Interceptor LLIN<br>4 Olyset LLIN<br>5 Dawnet LLIN<br>6 Iconlife LLIN

			7 Yorkool LLIN<br>8 DK brand LLIN<br>9 Govt brand LLIN<br>10 Other LLIN<br>11 Other brand<br>12 DK brand
257	net_4_brand_other<br>Show the field ONLY if:<br>[net_4_brand] = '10'	If other selected, please specify:	notes
258	net_4_how<br>Show the field ONLY if:<br>[net_number] >= 4	Did you get the net through a mass distribution, during an antenatal care visit, or during an immunization visit?	radio<br>1 Yes (mass distribution)<br>2 Yes (ANC)<br>3 Yes (immunization visit)<br>4 No
259	net_4_where<br>Show the field ONLY if:<br>[net_number] >= 4	Where did you get the net?	radio<br>1 Govt. hospital<br>2 Govt. health facility<br>3 Hospital (PNFP/NGO)<br>4 Health facility (PNFP/NGO)<br>5 Private hospital/clinic<br>6 Pharmacy<br>7 Shop/market<br>8 Hawker<br>9 CHW<br>10 Religious institution<br>11 Other<br>12 Don't know
260	net_4_sleep_yn<br>Show the field ONLY if:<br>[net_number] >= 4	Did anyone sleep under this mosquito net last night?	radio<br>1 Yes<br>0 No<br>99 Not sure
261	net_4_sleep_num<br>Show the field ONLY if:<br>[net_4_sleep_yn] = '1'	How many people slept under this mosquito net last night?	text (integer, Min: 1, Max: 10), Identifier
262	net_5_seen<br>Show the field ONLY if:<br>[net_number] >= 5	Net #5:	radio<br>1 Observed<br>0 Not observed
263	net_5_when<br>Show the field ONLY if:<br>[net_5_seen] = '1'	How many months ago did your household get the mosquito net?	radio<br>1 'Months ago' selected<br>2 More than 36 months ago<br>3 Not sure
264	net_5_months<br>Show the field ONLY if:<br>[net_5_when] = '1'	Months ago:	text (integer, Min: 1, Max: 36)

265	net_5_brand<br><br>Show the field ONLY if:<br>[net_5_seen] = '1'	Observe or ask brand/type of mosquito net.	radio<br>1 Permanent LLIN<br>2 Duranet LLIN<br>3 Interceptor LLIN<br>4 Olyset LLIN<br>5 Dawnet LLIN<br>6 Iconlife LLIN<br>7 Yorkool LLIN<br>8 DK brand LLIN<br>9 Govt brand LLIN<br>10 Other LLIN<br>11 Other brand<br>12 DK brand
266	net_5_brand_other<br><br>Show the field ONLY if:<br>[net_5_brand] = '10'	If other selected, please specify:	notes
267	net_5_how<br><br>Show the field ONLY if:<br>[net_number] >= 5	Did you get the net through a mass distribution, during an antenatal care visit, or during an immunization visit?	radio<br>1 Yes (mass distribution)<br>2 Yes (ANC)<br>3 Yes (immunization visit)<br>4 No
268	net_5_where<br><br>Show the field ONLY if:<br>[net_number] >= 5	Where did you get the net?	radio<br>1 Govt. hospital<br>2 Govt. health facility<br>3 Hospital (PNFP/NGO)<br>4 Health facility (PNFP/NGO)<br>5 Private hospital/clinic<br>6 Pharmacy<br>7 Shop/market<br>8 Hawker<br>9 CHW<br>10 Religious institution<br>11 Other<br>12 Don't know
269	net_5_sleep_yn<br><br>Show the field ONLY if:<br>[net_number] >= 5	Did anyone sleep under this mosquito net last night?	radio<br>1 Yes<br>0 No<br>99 Not sure
270	net_5_sleep_num<br><br>Show the field ONLY if:<br>[net_5_sleep_yn] = '1'	How many people slept under this mosquito net last night?	text (integer, Min: 1, Max: 10), Identifier
271	net_6_seen<br><br>Show the field ONLY if:<br>[net_number] >= 6	Net #6:	radio<br>1 Observed<br>0 Not observed

272	net_6_when<br>Show the field ONLY if:<br>[net_6_seen] = '1'	How many months ago did your household get the mosquito net?	radio<br>1 'Months ago' selected<br>2 More than 36 months ago<br>3 Not sure
273	net_6_months<br>Show the field ONLY if:<br>[net_6_when] = '1'	Months ago:	text (integer, Min: 1, Max: 36)
274	net_6_brand<br>Show the field ONLY if:<br>[net_6_seen] = '1'	Observe or ask brand/type of mosquito net.	radio<br>1 Permanent LLIN<br>2 Duranet LLIN<br>3 Interceptor LLIN<br>4 Olyset LLIN<br>5 Dawnet LLIN<br>6 Iconlife LLIN<br>7 Yorkool LLIN<br>8 DK brand LLIN<br>9 Govt brand LLIN<br>10 Other LLIN<br>11 Other brand<br>12 DK brand
275	net_6_brand_other<br>Show the field ONLY if:<br>[net_6_brand] = '10'	If other selected, please specify:	notes
276	net_6_how<br>Show the field ONLY if:<br>[net_number] >= 6	Did you get the net through a mass distribution, during an antenatal care visit, or during an immunization visit?	radio<br>1 Yes (mass distribution)<br>2 Yes (ANC)<br>3 Yes (immunization visit)<br>4 No
277	net_6_where<br>Show the field ONLY if:<br>[net_number] >= 6	Where did you get the net?	radio<br>1 Govt. hospital<br>2 Govt. health facility<br>3 Hospital (PNFP/NGO)<br>4 Health facility (PNFP/NGO)<br>5 Private hospital/clinic<br>6 Pharmacy<br>7 Shop/market<br>8 Hawker<br>9 CHW<br>10 Religious institution<br>11 Other<br>12 Don't know
278	net_6_sleep_yn<br>Show the field ONLY if:<br>[net_number] >= 6	Did anyone sleep under this mosquito net last night?	radio<br>1 Yes

			0 No<br>99 Not sure
279	net_6_sleep_num<br>Show the field ONLY if:<br>[net_6_sleep_yn] = '1'	How many people slept under this mosquito net last night?	text (integer, Min: 1, Max: 10), Identifier
280	net_7plus<br>Show the field ONLY if:<br>[net_number] >= 7	If more than six nets in the home, please describe information for the remaining:	notes
281	household_visit_malaria_and_care_seeking_complete	Section Header: *Form Status*<br>Complete?	dropdown<br>0 Incomplete<br>1 Unverified<br>2 Complete
**Instrument: 7. Household Visit - Testing and Treatment (household_visit_testing_and_treatment)**			
282	hh4_child_rdt	Section Header: *7.A. Child*<br>7.A.1. Child tested for malaria with a RDT?	yesno, Required<br>1 Yes<br>0 No
283	hh4_child_rdt_res	7.A.2. RDT Result	radio, Required<br>1 Positive<br>0 Negative
284	hh4_child_al<br>Show the field ONLY if:<br>[hh4_child_rdt_res] = '1'	Treatment with Coartem given?	yesno<br>1 Yes<br>0 No
285	hh4_mom_rdt	Section Header: *7.B. Mother*<br>7.B.1. Mother tested for malaria with a RDT?	yesno, Required<br>1 Yes<br>0 No
286	hh4_mom_rdt_res	7.B.2. RDT Result	radio, Required<br>1 Positive<br>0 Negative
287	hh4_mom_al<br>Show the field ONLY if:<br>[hh4_mom_rdt_res] = '1'	Treatment with Coartem given?	yesno<br>1 Yes<br>0 No
288	household_visit_testing_and_treatment_complete	Section Header: *Form Status*<br>Complete?	dropdown<br>0 Incomplete<br>1 Unverified<br>2 Complete
**Instrument: 8. Randomization (randomization)**			
289	r_id	Section Header: *Randomization Module*<br>Please confirm the subject's study ID	text, Identifier
290	r_check_id_y<br>Show the field ONLY if:<br>[study_id]=[r_id] and [r_id]<>''	PID confirmed - click 'Proceed with Randomization' button	radio<br>1 Proceed with Randomization
291	r_check_id_n<br>Show the field ONLY if:<br>[study_id]<>[r_id] and [r_id]<>''	PID incorrectly entered - please check previous fields and correct any typos	descriptive

292	r_site<br><br>Show the field ONLY if:<br>[r_check_id_y]='1'	Which site is the subject enrolled at?	radio<br>1 Bugoye<br>2 Mukathi
293	r_site_confirm<br><br>Show the field ONLY if:<br>[r_site]='1' or [r_site]='2'	You have selected the [r_site] site. Is this correct?	yesno<br>1 Yes<br>0 No
294	r_age<br><br>Show the field ONLY if:<br>[r_site_confirm]='1'	What is the child's age?	radio<br>1 6-11 months<br>2 12-18 months
295	r_age_confirm<br><br>Show the field ONLY if:<br>[r_age]='1' or [r_age]='2'	You have selected [r_age]. Is this correct?	yesno<br>1 Yes<br>0 No
296	r_dt<br><br>Show the field ONLY if:<br>[r_age_confirm]='1' and [r_site_confirm] = '1'	Randomization date/time	text (datetime_dmy), Identifier
297	r_rand_id<br><br>Show the field ONLY if:<br>[r_age_confirm]='1' and [r_site_confirm] = '1'	Randomization<br>*Randomization ID*	dropdown, Identifier<br>1001 1001<br>1002 1002<br>1003 1003<br>1004 1004<br>1005 1005<br>1006 1006<br>1007 1007<br>1008 1008<br>1009 1009<br>1010 1010<br>1011 1011<br>1012 1012<br>1013 1013<br>1014 1014<br>1015 1015<br>1016 1016<br>1017 1017<br>1018 1018<br>1019 1019<br>1020 1020<br>1021 1021<br>1022 1022<br>1023 1023<br>1024 1024<br>1025 1025<br>1026 1026<br>1027 1027

1028	1028
1029	1029
1030	1030
1031	1031
1032	1032
1033	1033
1034	1034
1035	1035
1036	1036
1037	1037
1038	1038
1039	1039
1040	1040
1041	1041
1042	1042
1043	1043
1044	1044
1045	1045
1046	1046
1047	1047
1048	1048
1049	1049
1050	1050
1051	1051
1052	1052
1053	1053
1054	1054
1055	1055
1056	1056
1057	1057
1058	1058
1059	1059
1060	1060
1061	1061
1062	1062
1063	1063
1064	1064
1065	1065
1066	1066
1067	1067
1068	1068
1069	1069
1070	1070

1071	1071
1072	1072
1073	1073
1074	1074
1075	1075
1076	1076
1077	1077
1078	1078
1079	1079
1080	1080
1081	1081
1082	1082
1083	1083
1084	1084
1085	1085
1086	1086
1087	1087
1088	1088
1089	1089
1090	1090
1091	1091
1092	1092
1093	1093
1094	1094
1095	1095
1096	1096
1097	1097
1098	1098
1099	1099
1100	1100
1101	1101
1102	1102
1103	1103
1104	1104
1105	1105
1106	1106
1107	1107
1108	1108
1109	1109
1110	1110
1111	1111
1112	1112
1113	1113

1114	1114
1115	1115
1116	1116
1117	1117
1118	1118
1119	1119
1120	1120
1121	1121
1122	1122
1123	1123
1124	1124
1125	1125
1126	1126
1127	1127
1128	1128
1129	1129
1130	1130
1131	1131
1132	1132
1133	1133
1134	1134
1135	1135
1136	1136
1137	1137
1138	1138
1139	1139
1140	1140
1141	1141
1142	1142
1143	1143
1144	1144
1145	1145
1146	1146
1147	1147
1148	1148
1149	1149
1150	1150
1151	1151
1152	1152
1153	1153
1154	1154
1155	1155
1156	1156
1157	1157

1158	1158
1159	1159
1160	1160
1161	1161
1162	1162
1163	1163
1164	1164
1165	1165
1166	1166
1167	1167
1168	1168
1169	1169
1170	1170
1171	1171
1172	1172
1173	1173
1174	1174
1175	1175
1176	1176
1177	1177
1178	1178
1179	1179
1180	1180
1181	1181
1182	1182
1183	1183
1184	1184
1185	1185
1186	1186
1187	1187
1188	1188
1189	1189
1190	1190
1191	1191
1192	1192
1193	1193
1194	1194
1195	1195
1196	1196
1197	1197
1198	1198
1199	1199
1200	1200

				1201	1201	
				1202	1202	
				1203	1203	
				1204	1204	
				1205	1205	
				1206	1206	
				1207	1207	
				1208	1208	
				1209	1209	
				1210	1210	
				1211	1211	
				1212	1212	
				1213	1213	
				1214	1214	
				1215	1215	
				1216	1216	
				1217	1217	
				1218	1218	
				1219	1219	
				1220	1220	
				1221	1221	
				1222	1222	
				1223	1223	
				1224	1224	
				1225	1225	
				1226	1226	
				1227	1227	
				1228	1228	
				1229	1229	
				1230	1230	
				1231	1231	
				1232	1232	
				1233	1233	
				1234	1234	
				1235	1235	
				1236	1236	
				1237	1237	
				1238	1238	
				1239	1239	
				1240	1240	
				1241	1241	
				1242	1242	
				1243	1243	

1244	1244
1245	1245
1246	1246
1247	1247
1248	1248
1249	1249
1250	1250
1251	1251
1252	1252
1253	1253
1254	1254
1255	1255
1256	1256
1257	1257
1258	1258
1259	1259
1260	1260
1261	1261
1262	1262
1263	1263
1264	1264
1265	1265
1266	1266
1267	1267
1268	1268
1269	1269
1270	1270
1271	1271
1272	1272
1273	1273
1274	1274
1275	1275
1276	1276
1277	1277
1278	1278
1279	1279
1280	1280
1281	1281
1282	1282
1283	1283
1284	1284
1285	1285
1286	1286
1287	1287

1288	1288
1289	1289
1290	1290
1291	1291
1292	1292
1293	1293
1294	1294
1295	1295
1296	1296
1297	1297
1298	1298
1299	1299
1300	1300
1301	1301
1302	1302
1303	1303
1304	1304
1305	1305
1306	1306
1307	1307
1308	1308
1309	1309
1310	1310
1311	1311
1312	1312
1313	1313
1314	1314
1315	1315
1316	1316
1317	1317
1318	1318
1319	1319
1320	1320
1321	1321
1322	1322
1323	1323
1324	1324
1325	1325
1326	1326
1327	1327
1328	1328
1329	1329
1330	1330

1331	1331
1332	1332
1333	1333
1334	1334
1335	1335
1336	1336
1337	1337
1338	1338
1339	1339
1340	1340
1341	1341
1342	1342
1343	1343
1344	1344
1345	1345
1346	1346
1347	1347
1348	1348
1349	1349
1350	1350
1351	1351
1352	1352
1353	1353
1354	1354
1355	1355
1356	1356
1357	1357
1358	1358
1359	1359
1360	1360
1361	1361
1362	1362
1363	1363
1364	1364
1365	1365
1366	1366
1367	1367
1368	1368
1369	1369
1370	1370
1371	1371
1372	1372
1373	1373

1374	1374
1375	1375
1376	1376
1377	1377
1378	1378
1379	1379
1380	1380
1381	1381
1382	1382
1383	1383
1384	1384
1385	1385
1386	1386
1387	1387
1388	1388
1389	1389
1390	1390
1391	1391
1392	1392
1393	1393
1394	1394
1395	1395
1396	1396
1397	1397
1398	1398
1399	1399
1400	1400
1801	1801
1802	1802
1803	1803
1804	1804
1805	1805
1806	1806
1807	1807
1808	1808
1809	1809
1810	1810
1811	1811
1812	1812
1813	1813
1814	1814
1815	1815
1816	1816
1817	1817

1818	1818
1819	1819
1820	1820
1821	1821
1822	1822
1823	1823
1824	1824
1825	1825
1826	1826
1827	1827
1828	1828
1829	1829
1830	1830
1831	1831
1832	1832
1833	1833
1834	1834
1835	1835
1836	1836
1837	1837
1838	1838
1839	1839
1840	1840
1841	1841
1842	1842
1843	1843
1844	1844
1845	1845
1846	1846
1847	1847
1848	1848
1849	1849
1850	1850
1851	1851
1852	1852
1853	1853
1854	1854
1855	1855
1856	1856
1857	1857
1858	1858
1859	1859
1860	1860

				1861	1861	
				1862	1862	
				1863	1863	
				1864	1864	
				1865	1865	
				1866	1866	
				1867	1867	
				1868	1868	
				1869	1869	
				1870	1870	
				1871	1871	
				1872	1872	
				1873	1873	
				1874	1874	
				1875	1875	
				1876	1876	
				1877	1877	
				1878	1878	
				1879	1879	
				1880	1880	
				1881	1881	
				1882	1882	
				1883	1883	
				1884	1884	
				1885	1885	
				1886	1886	
				1887	1887	
				1888	1888	
				1889	1889	
				1890	1890	
				1891	1891	
				1892	1892	
				1893	1893	
				1894	1894	
				1895	1895	
				1896	1896	
				1897	1897	
				1898	1898	
				1899	1899	
				1900	1900	
				1901	1901	
				1902	1902	
				1903	1903	
				1904	1904	

1905	1905
1906	1906
1907	1907
1908	1908
1909	1909
1910	1910
1911	1911
1912	1912
1913	1913
1914	1914
1915	1915
1916	1916
1917	1917
1918	1918
1919	1919
1920	1920
1921	1921
1922	1922
1923	1923
1924	1924
1925	1925
1926	1926
1927	1927
1928	1928
1929	1929
1930	1930
1931	1931
1932	1932
1933	1933
1934	1934
1935	1935
1936	1936
1937	1937
1938	1938
1939	1939
1940	1940
1941	1941
1942	1942
1943	1943
1944	1944
1945	1945
1946	1946
1947	1947

1948	1948
1949	1949
1950	1950
1951	1951
1952	1952
1953	1953
1954	1954
1955	1955
1956	1956
1957	1957
1958	1958
1959	1959
1960	1960
1961	1961
1962	1962
1963	1963
1964	1964
1965	1965
1966	1966
1967	1967
1968	1968
1969	1969
1970	1970
1971	1971
1972	1972
1973	1973
1974	1974
1975	1975
1976	1976
1977	1977
1978	1978
1979	1979
1980	1980
1981	1981
1982	1982
1983	1983
1984	1984
1985	1985
1986	1986
1987	1987
1988	1988
1989	1989
1990	1990

1991	1991
1992	1992
1993	1993
1994	1994
1995	1995
1996	1996
1997	1997
1998	1998
1999	1999
2000	2000
2001	2001
2002	2002
2003	2003
2004	2004
2005	2005
2006	2006
2007	2007
2008	2008
2009	2009
2010	2010
2011	2011
2012	2012
2013	2013
2014	2014
2015	2015
2016	2016
2017	2017
2018	2018
2019	2019
2020	2020
2021	2021
2022	2022
2023	2023
2024	2024
2025	2025
2026	2026
2027	2027
2028	2028
2029	2029
2030	2030
2031	2031
2032	2032
2033	2033

2034	2034
2035	2035
2036	2036
2037	2037
2038	2038
2039	2039
2040	2040
2041	2041
2042	2042
2043	2043
2044	2044
2045	2045
2046	2046
2047	2047
2048	2048
2049	2049
2050	2050
2051	2051
2052	2052
2053	2053
2054	2054
2055	2055
2056	2056
2057	2057
2058	2058
2059	2059
2060	2060
2061	2061
2062	2062
2063	2063
2064	2064
2065	2065
2066	2066
2067	2067
2068	2068
2069	2069
2070	2070
2071	2071
2072	2072
2073	2073
2074	2074
2075	2075
2076	2076

2077	2077
2078	2078
2079	2079
2080	2080
2081	2081
2082	2082
2083	2083
2084	2084
2085	2085
2086	2086
2087	2087
2088	2088
2089	2089
2090	2090
2091	2091
2092	2092
2093	2093
2094	2094
2095	2095
2096	2096
2097	2097
2098	2098
2099	2099
2100	2100
2101	2101
2102	2102
2103	2103
2104	2104
2105	2105
2106	2106
2107	2107
2108	2108
2109	2109
2110	2110
2111	2111
2112	2112
2113	2113
2114	2114
2115	2115
2116	2116
2117	2117
2118	2118
2119	2119
2120	2120

			2121	2121	
			2122	2122	
			2123	2123	
			2124	2124	
			2125	2125	
			2126	2126	
			2127	2127	
			2128	2128	
			2129	2129	
			2130	2130	
			2131	2131	
			2132	2132	
			2133	2133	
			2134	2134	
			2135	2135	
			2136	2136	
			2137	2137	
			2138	2138	
			2139	2139	
			2140	2140	
			2141	2141	
			2142	2142	
			2143	2143	
			2144	2144	
			2145	2145	
			2146	2146	
			2147	2147	
			2148	2148	
			2149	2149	
			2150	2150	
			2151	2151	
			2152	2152	
			2153	2153	
			2154	2154	
			2155	2155	
			2156	2156	
			2157	2157	
			2158	2158	
			2159	2159	
			2160	2160	
			2161	2161	
			2162	2162	
			2163	2163	

				2164	2164
				2165	2165
				2166	2166
				2167	2167
				2168	2168
				2169	2169
				2170	2170
				2171	2171
				2172	2172
				2173	2173
				2174	2174
				2175	2175
				2176	2176
				2177	2177
				2178	2178
				2179	2179
				2180	2180
				2181	2181
				2182	2182
				2183	2183
				2184	2184
				2185	2185
				2186	2186
				2187	2187
				2188	2188
				2189	2189
				2190	2190
				2191	2191
				2192	2192
				2193	2193
				2194	2194
				2195	2195
				2196	2196
				2197	2197
				2198	2198
				2199	2199
				2200	2200
				1401	1401
				1402	1402
				1403	1403
				1404	1404
				1405	1405
				1406	1406

				1407	1407
				1408	1408
				1409	1409
				1410	1410
				1411	1411
				1412	1412
				1413	1413
				1414	1414
				1415	1415
				1416	1416
				1417	1417
				1418	1418
				1419	1419
				1420	1420
				1421	1421
				1422	1422
				1423	1423
				1424	1424
				1425	1425
				1426	1426
				1427	1427
				1428	1428
				1429	1429
				1430	1430
				1431	1431
				1432	1432
				1433	1433
				1434	1434
				1435	1435
				1436	1436
				1437	1437
				1438	1438
				1439	1439
				1440	1440
				1441	1441
				1442	1442
				1443	1443
				1444	1444
				1445	1445
				1446	1446
				1447	1447
				1448	1448
				1449	1449

				1450	1450	
				1451	1451	
				1452	1452	
				1453	1453	
				1454	1454	
				1455	1455	
				1456	1456	
				1457	1457	
				1458	1458	
				1459	1459	
				1460	1460	
				1461	1461	
				1462	1462	
				1463	1463	
				1464	1464	
				1465	1465	
				1466	1466	
				1467	1467	
				1468	1468	
				1469	1469	
				1470	1470	
				1471	1471	
				1472	1472	
				1473	1473	
				1474	1474	
				1475	1475	
				1476	1476	
				1477	1477	
				1478	1478	
				1479	1479	
				1480	1480	
				1481	1481	
				1482	1482	
				1483	1483	
				1484	1484	
				1485	1485	
				1486	1486	
				1487	1487	
				1488	1488	
				1489	1489	
				1490	1490	
				1491	1491	
				1492	1492	

1493	1493
1494	1494
1495	1495
1496	1496
1497	1497
1498	1498
1499	1499
1500	1500
1501	1501
1502	1502
1503	1503
1504	1504
1505	1505
1506	1506
1507	1507
1508	1508
1509	1509
1510	1510
1511	1511
1512	1512
1513	1513
1514	1514
1515	1515
1516	1516
1517	1517
1518	1518
1519	1519
1520	1520
1521	1521
1522	1522
1523	1523
1524	1524
1525	1525
1526	1526
1527	1527
1528	1528
1529	1529
1530	1530
1531	1531
1532	1532
1533	1533
1534	1534
1535	1535

1536	1536
1537	1537
1538	1538
1539	1539
1540	1540
1541	1541
1542	1542
1543	1543
1544	1544
1545	1545
1546	1546
1547	1547
1548	1548
1549	1549
1550	1550
1551	1551
1552	1552
1553	1553
1554	1554
1555	1555
1556	1556
1557	1557
1558	1558
1559	1559
1560	1560
1561	1561
1562	1562
1563	1563
1564	1564
1565	1565
1566	1566
1567	1567
1568	1568
1569	1569
1570	1570
1571	1571
1572	1572
1573	1573
1574	1574
1575	1575
1576	1576
1577	1577
1578	1578

1579	1579
1580	1580
1581	1581
1582	1582
1583	1583
1584	1584
1585	1585
1586	1586
1587	1587
1588	1588
1589	1589
1590	1590
1591	1591
1592	1592
1593	1593
1594	1594
1595	1595
1596	1596
1597	1597
1598	1598
1599	1599
1600	1600
1601	1601
1602	1602
1603	1603
1604	1604
1605	1605
1606	1606
1607	1607
1608	1608
1609	1609
1610	1610
1611	1611
1612	1612
1613	1613
1614	1614
1615	1615
1616	1616
1617	1617
1618	1618
1619	1619
1620	1620
1621	1621

1622	1622
1623	1623
1624	1624
1625	1625
1626	1626
1627	1627
1628	1628
1629	1629
1630	1630
1631	1631
1632	1632
1633	1633
1634	1634
1635	1635
1636	1636
1637	1637
1638	1638
1639	1639
1640	1640
1641	1641
1642	1642
1643	1643
1644	1644
1645	1645
1646	1646
1647	1647
1648	1648
1649	1649
1650	1650
1651	1651
1652	1652
1653	1653
1654	1654
1655	1655
1656	1656
1657	1657
1658	1658
1659	1659
1660	1660
1661	1661
1662	1662
1663	1663
1664	1664

1665	1665
1666	1666
1667	1667
1668	1668
1669	1669
1670	1670
1671	1671
1672	1672
1673	1673
1674	1674
1675	1675
1676	1676
1677	1677
1678	1678
1679	1679
1680	1680
1681	1681
1682	1682
1683	1683
1684	1684
1685	1685
1686	1686
1687	1687
1688	1688
1689	1689
1690	1690
1691	1691
1692	1692
1693	1693
1694	1694
1695	1695
1696	1696
1697	1697
1698	1698
1699	1699
1700	1700
1701	1701
1702	1702
1703	1703
1704	1704
1705	1705
1706	1706
1707	1707

1708	1708
1709	1709
1710	1710
1711	1711
1712	1712
1713	1713
1714	1714
1715	1715
1716	1716
1717	1717
1718	1718
1719	1719
1720	1720
1721	1721
1722	1722
1723	1723
1724	1724
1725	1725
1726	1726
1727	1727
1728	1728
1729	1729
1730	1730
1731	1731
1732	1732
1733	1733
1734	1734
1735	1735
1736	1736
1737	1737
1738	1738
1739	1739
1740	1740
1741	1741
1742	1742
1743	1743
1744	1744
1745	1745
1746	1746
1747	1747
1748	1748
1749	1749
1750	1750

1751	1751
1752	1752
1753	1753
1754	1754
1755	1755
1756	1756
1757	1757
1758	1758
1759	1759
1760	1760
1761	1761
1762	1762
1763	1763
1764	1764
1765	1765
1766	1766
1767	1767
1768	1768
1769	1769
1770	1770
1771	1771
1772	1772
1773	1773
1774	1774
1775	1775
1776	1776
1777	1777
1778	1778
1779	1779
1780	1780
1781	1781
1782	1782
1783	1783
1784	1784
1785	1785
1786	1786
1787	1787
1788	1788
1789	1789
1790	1790
1791	1791
1792	1792
1793	1793

				1794	1794
				1795	1795
				1796	1796
				1797	1797
				1798	1798
				1799	1799
				1800	1800
				2201	2201
				2202	2202
				2203	2203
				2204	2204
				2205	2205
				2206	2206
				2207	2207
				2208	2208
				2209	2209
				2210	2210
				2211	2211
				2212	2212
				2213	2213
				2214	2214
				2215	2215
				2216	2216
				2217	2217
				2218	2218
				2219	2219
				2220	2220
				2221	2221
				2222	2222
				2223	2223
				2224	2224
				2225	2225
				2226	2226
				2227	2227
				2228	2228
				2229	2229
				2230	2230
				2231	2231
				2232	2232
				2233	2233
				2234	2234
				2235	2235
				2236	2236

			2237	2237	
			2238	2238	
			2239	2239	
			2240	2240	
			2241	2241	
			2242	2242	
			2243	2243	
			2244	2244	
			2245	2245	
			2246	2246	
			2247	2247	
			2248	2248	
			2249	2249	
			2250	2250	
			2251	2251	
			2252	2252	
			2253	2253	
			2254	2254	
			2255	2255	
			2256	2256	
			2257	2257	
			2258	2258	
			2259	2259	
			2260	2260	
			2261	2261	
			2262	2262	
			2263	2263	
			2264	2264	
			2265	2265	
			2266	2266	
			2267	2267	
			2268	2268	
			2269	2269	
			2270	2270	
			2271	2271	
			2272	2272	
			2273	2273	
			2274	2274	
			2275	2275	
			2276	2276	
			2277	2277	
			2278	2278	
			2279	2279	

2280	2280
2281	2281
2282	2282
2283	2283
2284	2284
2285	2285
2286	2286
2287	2287
2288	2288
2289	2289
2290	2290
2291	2291
2292	2292
2293	2293
2294	2294
2295	2295
2296	2296
2297	2297
2298	2298
2299	2299
2300	2300
2301	2301
2302	2302
2303	2303
2304	2304
2305	2305
2306	2306
2307	2307
2308	2308
2309	2309
2310	2310
2311	2311
2312	2312
2313	2313
2314	2314
2315	2315
2316	2316
2317	2317
2318	2318
2319	2319
2320	2320
2321	2321
2322	2322

2323	2323
2324	2324
2325	2325
2326	2326
2327	2327
2328	2328
2329	2329
2330	2330
2331	2331
2332	2332
2333	2333
2334	2334
2335	2335
2336	2336
2337	2337
2338	2338
2339	2339
2340	2340
2341	2341
2342	2342
2343	2343
2344	2344
2345	2345
2346	2346
2347	2347
2348	2348
2349	2349
2350	2350
2351	2351
2352	2352
2353	2353
2354	2354
2355	2355
2356	2356
2357	2357
2358	2358
2359	2359
2360	2360
2361	2361
2362	2362
2363	2363
2364	2364
2365	2365

2366	2366
2367	2367
2368	2368
2369	2369
2370	2370
2371	2371
2372	2372
2373	2373
2374	2374
2375	2375
2376	2376
2377	2377
2378	2378
2379	2379
2380	2380
2381	2381
2382	2382
2383	2383
2384	2384
2385	2385
2386	2386
2387	2387
2388	2388
2389	2389
2390	2390
2391	2391
2392	2392
2393	2393
2394	2394
2395	2395
2396	2396
2397	2397
2398	2398
2399	2399
2400	2400
2401	2401
2402	2402
2403	2403
2404	2404
2405	2405
2406	2406
2407	2407
2408	2408

			2409	2409	
			2410	2410	
			2411	2411	
			2412	2412	
			2413	2413	
			2414	2414	
			2415	2415	
			2416	2416	
			2417	2417	
			2418	2418	
			2419	2419	
			2420	2420	
			2421	2421	
			2422	2422	
			2423	2423	
			2424	2424	
			2425	2425	
			2426	2426	
			2427	2427	
			2428	2428	
			2429	2429	
			2430	2430	
			2431	2431	
			2432	2432	
			2433	2433	
			2434	2434	
			2435	2435	
			2436	2436	
			2437	2437	
			2438	2438	
			2439	2439	
			2440	2440	
			2441	2441	
			2442	2442	
			2443	2443	
			2444	2444	
			2445	2445	
			2446	2446	
			2447	2447	
			2448	2448	
			2449	2449	
			2450	2450	
			2451	2451	

2452	2452
2453	2453
2454	2454
2455	2455
2456	2456
2457	2457
2458	2458
2459	2459
2460	2460
2461	2461
2462	2462
2463	2463
2464	2464
2465	2465
2466	2466
2467	2467
2468	2468
2469	2469
2470	2470
2471	2471
2472	2472
2473	2473
2474	2474
2475	2475
2476	2476
2477	2477
2478	2478
2479	2479
2480	2480
2481	2481
2482	2482
2483	2483
2484	2484
2485	2485
2486	2486
2487	2487
2488	2488
2489	2489
2490	2490
2491	2491
2492	2492
2493	2493
2494	2494

			2495	2495	
			2496	2496	
			2497	2497	
			2498	2498	
			2499	2499	
			2500	2500	
			2501	2501	
			2502	2502	
			2503	2503	
			2504	2504	
			2505	2505	
			2506	2506	
			2507	2507	
			2508	2508	
			2509	2509	
			2510	2510	
			2511	2511	
			2512	2512	
			2513	2513	
			2514	2514	
			2515	2515	
			2516	2516	
			2517	2517	
			2518	2518	
			2519	2519	
			2520	2520	
			2521	2521	
			2522	2522	
			2523	2523	
			2524	2524	
			2525	2525	
			2526	2526	
			2527	2527	
			2528	2528	
			2529	2529	
			2530	2530	
			2531	2531	
			2532	2532	
			2533	2533	
			2534	2534	
			2535	2535	
			2536	2536	
			2537	2537	

2538	2538
2539	2539
2540	2540
2541	2541
2542	2542
2543	2543
2544	2544
2545	2545
2546	2546
2547	2547
2548	2548
2549	2549
2550	2550
2551	2551
2552	2552
2553	2553
2554	2554
2555	2555
2556	2556
2557	2557
2558	2558
2559	2559
2560	2560
2561	2561
2562	2562
2563	2563
2564	2564
2565	2565
2566	2566
2567	2567
2568	2568
2569	2569
2570	2570
2571	2571
2572	2572
2573	2573
2574	2574
2575	2575
2576	2576
2577	2577
2578	2578
2579	2579
2580	2580

			2581 2581<br>2582 2582<br>2583 2583<br>2584 2584<br>2585 2585<br>2586 2586<br>2587 2587<br>2588 2588<br>2589 2589<br>2590 2590<br>2591 2591<br>2592 2592<br>2593 2593<br>2594 2594<br>2595 2595<br>2596 2596<br>2597 2597<br>2598 2598<br>2599 2599<br>2600 2600
298	r_comment_yn<br>Show the field ONLY if:<br>[r_age_confirm]='1' and [r_site_confirm] = '1'	Are there any comments about randomization?	radio<br>1 Yes<br>0 No
299	r_comment<br>Show the field ONLY if:<br>[r_comment_yn]=1	Comments:	notes
300	randomization_complete	Section Header: *Form Status*<br>Complete?	dropdown<br>0 Incomplete<br>1 Unverified<br>2 Complete
**Instrument: 9. Initial Clinic Visit (initial_clinic_visit)**			
301	unique	Confirm Study ID<br>*Example BUG-001*	text, Identifier
302	date_visit_v0	Section Header: *Initial Visit*<br>Date of visit<br>*DD-MM-YYYY*	text (date_dmy), Required
303	height_v0	Section Header: *I. Vital Signs - Child*<br>Height<br>*cm*	text (number, Min: 50, Max: 100), Required
304	weight_v0	Weight<br>*kg*	text (number, Min: 5, Max: 20), Required
305	muac_v0	Mid-Upper Arm Circumference<br>*cm*	text (number, Min: 5, Max: 25), Required
306	temp_v0	Axillary temperature<br>*degrees Celsius*	text (number, Min: 35, Max: 45), Required
307	bednet_v0	Section Header: *II. Medical History - Child*	yesno, Required

		Did the child sleep under a bed net last night?	1 Yes<br>0 No
308	fever_v0	Has the child had fever in last two weeks?	yesno, Required<br>1 Yes<br>0 No
309	onset_v0<br>Show the field ONLY if:<br>[fever_v0] = '1'	If yes, when did the fever start?<br>*DD-MM-YYYY*	text (date_dmy)
310	sick_v0<br>Show the field ONLY if:<br>[fever_v0] = '0'	Even if the child has not had a fever, has he or she been otherwise unwell?	yesno, Required<br>1 Yes<br>0 No
311	symp_v0<br>Show the field ONLY if:<br>[sick_v0] = '1'	If yes, what symptoms has the child experienced?	checkbox<br>0 symp_v0___0 Cough<br>1 symp_v0___1 Diarrhea<br>2 symp_v0___2 Ear Ache<br>3 symp_v0___3 Not feeding<br>4 symp_v0___4 Rash<br>5 symp_v0___5 Runny nose<br>6 symp_v0___6 Other (specify below)
312	symp_other_v0<br>Show the field ONLY if:<br>[symp_v0(6)] = '1'	List other symptoms:	notes
313	healthcentre_v0<br>Show the field ONLY if:<br>[fever_v0] = '1' or [sick_v0] = '1'	Has the child been seen at a hospital, health centre, clinic, drug shop, or other medical attendant for these symptoms?	yesno<br>1 Yes<br>0 No
314	hc_where_v0<br>Show the field ONLY if:<br>[healthcentre_v0] = '1'	If yes, where?	radio<br>0 Hospital<br>1 Health Centre<br>2 Drug Shop or Pharmacy<br>3 VHT<br>4 Traditional Medicine
315	medicine_v0<br>Show the field ONLY if:<br>[healthcentre_v0] = '1'	Did the child receive medicine for malaria?	yesno<br>1 Yes<br>0 No
316	med_date_v0<br>Show the field ONLY if:<br>[medicine_v0] = '1'	When did the child take the last dose (i.e. pill) of medicine?<br>*DD-MM-YYYY*	text (date_dmy)
317	mrdt_v0	Section Header: *III. Laboratory Testing - Child*<br>Malaria RDT performed?	yesno, Required<br>1 Yes<br>0 No
318	mrdt_res_v0<br>Show the field ONLY if:<br>[mrdt_v0] = '1'	Malaria RDT Result<br>*Repeat any invalid tests*	radio<br>0 Negative<br>1 Positive

319	treat_v0<br>Show the field ONLY if:<br>([temp_v0] >= 37.5 or [fever_v0] = '1') and [mrdt_res_v0] = '1'	If mother reported fever or child's temperature was >37.5 C, which antimalarial treatment provided?	radio<br>0 None (explain below)<br>1 Coartem<br>2 Quinine<br>3 Admitted
320	no_treat_v0<br>Show the field ONLY if:<br>[treat_v0] = '0'	Why was treatment NOT given?	notes
321	cbc_v0	Hemoglobin measured?	yesno<br>1 Yes<br>0 No
322	hb_v0<br>Show the field ONLY if:<br>[cbc_v0] = '1'	Hemoglobin (g/dL)	text (number, Min: 3, Max: 20)
323	dbs_v0	Dried blood spots collected?	yesno<br>1 Yes<br>0 No
324	urine_v0	Urine sample collected?	yesno<br>1 Yes<br>0 No
325	bednet_mot_v0	Section Header: *IV. Medical History - Mother*<br>Did the mother sleep under a bed net last night?	yesno, Required<br>1 Yes<br>0 No
326	cosleep_v0<br>Show the field ONLY if:<br>[bednet_mot_v0] = '1'	Did the mother sleep under the same net as the child?	yesno<br>1 Yes<br>0 No
327	cbc_mot_v0	Section Header: *V. Laboratory Testing - Mother*<br>Hemoglobin measured?	yesno<br>1 Yes<br>0 No
328	hb_mot_v0<br>Show the field ONLY if:<br>[cbc_mot_v0] = '1'	Hemoglobin (g/dL)	text (number, Min: 3, Max: 20)
329	dbs_mot_v0	Dried blood spots collected?	yesno<br>1 Yes<br>0 No
330	urine_mot_v0	Urine sample collected?	yesno<br>1 Yes<br>0 No
331	dc_v0	Section Header: *VI. Discharge Actions*<br>Prior to discharge, ensure the following are complete:	checkbox<br>0 dc_v0___0 Provide with Study ID card<br>1 dc_v0___1 Give new Lesu according to assignment<br>2 dc_v0___2 Instruct to return to clinic with card if child sick<br>3 dc_v0___3 Remind about next scheduled visit in

			2 weeks
332	initial_clinic_visit_complete	Section Header: *Form Status*<br>Complete?	dropdown<br>0 Incomplete<br>1 Unverified<br>2 Complete
**Instrument: 10. Week 2 Clinic Visit (week_2_clinic_visit)**			
333	date_visit_v2	Section Header: *Week 2 Visit*<br>Date of visit<br>*DD-MM-YYYY*	text (date_dmy), Required
334	temp_v2	Section Header: *I. Vital Signs - Child*<br>Axillary temperature<br>*degrees Celsius*	text (number, Min: 35, Max: 45), Required
335	bednet_v2	Section Header: *II. Medical History - Child*<br>Did the child sleep under a bed net last night?	yesno, Required<br>1 Yes<br>0 No
336	fever_v2	Has the child had fever in last two weeks?	yesno, Required<br>1 Yes<br>0 No
337	onset_v2<br>Show the field ONLY if:<br>[fever_v2] = '1'	If yes, when did the fever start<br>*DD-MM-YYYY*	text (date_dmy)
338	sick_v2<br>Show the field ONLY if:<br>[fever_v2] = '0' OR [fever_v2] = '1'	Even if the child has not had a fever, has he or she been otherwise unwell?	yesno, Required<br>1 Yes<br>0 No
339	symp_v2<br>Show the field ONLY if:<br>[sick_v2] = '1'	If yes, what symptoms has the child experienced?	checkbox<br>0 symp_v2___0 Cough<br>1 symp_v2___1 Diarrhea<br>2 symp_v2___2 Ear Ache<br>3 symp_v2___3 Not feeding<br>4 symp_v2___4 Rash<br>5 symp_v2___5 Runny nose<br>6 symp_v2___6 Other (specify below)
340	symp_other_v2<br>Show the field ONLY if:<br>[symp_v2(6)] = '1'	List other symptoms:	notes
341	healthcentre_v2<br>Show the field ONLY if:<br>[fever_v2] = '1' or [sick_v2] = '1'	Has the child been seen at a hospital, health centre, clinic, drug shop, or other medical attendant for these symptoms?	yesno<br>1 Yes<br>0 No
342	hc_where_v2<br>Show the field ONLY if:<br>[healthcentre_v2] = '1'	If yes, where?	radio<br>0 Hospital<br>1 Health Centre<br>2 Drug Shop or Pharmacy<br>3 VHT<br>4 Traditional Medicine

343	medicine_v2<br>Show the field ONLY if:<br>[healthcentre_v2] = '1'	Did the child receive medicine for malaria?	yesno<br>1 Yes<br>0 No
344	med_date_v2<br>Show the field ONLY if:<br>[medicine_v2] = '1'	When did the child take the last dose (i.e. pill) of medicine?<br>*DD-MM-YYYY*	text (date_dmy)
345	mrdt_v2	Section Header: *III. Laboratory Testing - Child*<br>Malaria RDT performed?	yesno, Required<br>1 Yes<br>0 No
346	mrdt_res_v2<br>Show the field ONLY if:<br>[mrdt_v2] = '1'	Malaria RDT Result<br>*Repeat any invalid tests*	radio<br>0 Negative<br>1 Positive
347	treat_v2<br>Show the field ONLY if:<br>([temp_v2] >= 37.5 or [fever_v2] = '1') and [mrdt_res_v2] = '1'	If mother reported fever or child's temperature was >37.5 C, which antimalarial treatment provided?	radio<br>0 None (explain below)<br>1 Coartem<br>2 Quinine<br>3 Admitted
348	no_treat_v2<br>Show the field ONLY if:<br>[treat_v2] = '0'	Why was treatment NOT given?	notes
349	dbs_v2	Dried blood spots collected?	yesno<br>1 Yes<br>0 No
350	lesu_use_v2	Section Header: *IV. Lesu Questions*<br>Since your last visit, how often did you use the lesu to carry the child?	radio, Required<br>0 Never<br>1 Some days (1 - 3 per week)<br>2 Most days (4 - 6 per week)<br>3 Every day
351	washing_v2	Since your last visit, how many times did you wash the lesu?	text (integer, Min: 0, Max: 50), Required
352	se_v2	Did the child experience any side effects, to include itching or rash, from the lesu?	yesno, Required<br>1 Yes<br>0 No
353	se_symp_v2<br>Show the field ONLY if:<br>[se_v2] = '1'	If yes, what were the child's side effects?	checkbox<br>0 se_symp_v2___0 Headache<br>1 se_symp_v2___1 Itching<br>2 se_symp_v2___2 Nausea or not feeding<br>3 se_symp_v2___3 Rash<br>4 se_symp_v2___4 Other
354	se_other_v2<br>Show the field ONLY if:<br>[se_symp_v2(4)] = '1'	Describe the child's other symptoms:	notes
355	se_impact_v2<br>Show the field ONLY if:<br>[se_v2] = '1'	Did the side effects make you stop using the lesu or use the lesu less frequently?	radio<br>0 No change in use<br>1 Used it less frequently

			2 Stopped using it
356	se_mot_v2	Did the mother experience any side effects, to include itching or rash, from the lesu?	yesno, Required<br>1 Yes<br>0 No
357	se_symp_mot_v2<br>Show the field ONLY if:<br>[se_mot_v2] = '1'	If yes, what were the side effects?	checkbox<br>0 se_symp_mot_v2___0 Headache<br>1 se_symp_mot_v2___1 Itching<br>2 se_symp_mot_v2___2 Nausea or loss of appetite<br>3 se_symp_mot_v2___3 Rash<br>4 se_symp_mot_v2___4 Other
358	se_mot_other_v2<br>Show the field ONLY if:<br>[se_symp_mot_v2(4)] = '1'	Describe other symptoms:	notes
359	diary_lesu1_v2	Section Header: *V. Lesu Diary*<br>How many days did the participant report using LESU #1<br>*Enter number between 0 to 14*	text (integer, Min: 0, Max: 14)
360	diary_lesu2_v2	How many days did the participant report using LESU #2<br>*Enter number between 0 to 14*	text (integer, Min: 0, Max: 14)
361	diary_llin_mom_v2	How many nights did the MOTHER report sleeping under a bed net?<br>*Enter number between 0 to 14*	text (integer, Min: 0, Max: 14)
362	diary_llin_child_v2	How many nights did the CHILD report sleeping under a bed net?<br>*Enter number between 0 to 14*	text (integer, Min: 0, Max: 14)
363	diary_carry_v2	How many days was a lesu used to CARRY the child?<br>*Enter number between 0 to 14*	text (integer, Min: 0, Max: 14)
364	diary_sit_v2	How many days was a lesu used as a place for the child to SIT?<br>*Enter number between 0 to 14*	text (integer, Min: 0, Max: 14)
365	diary_sleep_v2	How many days was a lesu used as a BLANKET when the child was put to sleep?<br>*Enter number between 0 to 14*	text (integer, Min: 0, Max: 14)
366	diary_wash_v2	How many days was LESU #1 WASHED?<br>*Enter number between 0 to 14*	text (integer, Min: 0, Max: 14)
367	diary_wash2_v2	How many days was LESU #2 WASHED?<br>*Enter number between 0 to 14*	text (integer, Min: 0, Max: 14)
368	se_impact_mot_v2<br>Show the field ONLY if:<br>[se_mot_v2] = '1'	Section Header: *VI. Discharge Actions*<br>Did these side effects make you stop using the lesu or use the lesu less frequently?	radio<br>0 No change in use<br>1 Used it less frequently<br>2 Stopped using it
369	dc_v2	Prior to discharge, ensure the following are complete:	checkbox<br>2 dc_v2___2 Instruct to return to clinic with card if child sick<br>3 dc_v2___3 Remind about next scheduled visit in 2 weeks
370	week_2_clinic_visit_complete	Section Header: *Form Status*<br>Complete?	dropdown<br>0 Incomplete<br>1 Unverified<br>2 Complete

Instrument: 11. Week 4 Clinic Visit (week_4_clinic_visit)

371	date_visit_v4	Section Header: *Week 4 Visit*<br>Date of visit<br>*DD-MM-YYYY*	text (date_dmy), Required
372	temp_v4	Section Header: *I. Vital Signs - Child*<br>Axillary temperature<br>*degrees Celsius*	text (number, Min: 35, Max: 45), Required
373	bednet_v4	Section Header: *II. Medical History - Child*<br>Did the child sleep under a bed net last night?	yesno, Required<br>1 Yes<br>0 No
374	fever_v4	Has the child had fever in last two weeks?	yesno, Required<br>1 Yes<br>0 No
375	onset_v4<br>Show the field ONLY if:<br>[fever_v4] = '1'	If yes, when did the fever start<br>*DD-MM-YYYY*	text (date_dmy)
376	sick_v4<br>Show the field ONLY if:<br>[fever_v4] = '0' OR [fever_v4] = '1'	Even if the child has not had a fever, has he or she been otherwise unwell?	yesno, Required<br>1 Yes<br>0 No
377	symp_v4<br>Show the field ONLY if:<br>[sick_v4] = '1'	If yes, what symptoms has the child experienced?	checkbox<br>0 symp_v4___0 Cough<br>1 symp_v4___1 Diarrhea<br>2 symp_v4___2 Ear Ache<br>3 symp_v4___3 Not feeding<br>4 symp_v4___4 Rash<br>5 symp_v4___5 Runny nose<br>6 symp_v4___6 Other (specify below)
378	symp_other_v4<br>Show the field ONLY if:<br>[symp_v4(6)] = '1'	List other symptoms:	notes
379	healthcentre_v4<br>Show the field ONLY if:<br>[fever_v4] = '1' or [sick_v4] = ' 1'	Has the child been seen at a hospital, health centre, clinic, drug shop, or other medical attendant for these symptoms?	yesno<br>1 Yes<br>0 No
380	hc_where_v4<br>Show the field ONLY if:<br>[healthcentre_v4] = '1'	If yes, where?	radio<br>0 Hospital<br>1 Health Centre<br>2 Drug Shop or Pharmacy<br>3 VHT<br>4 Traditional Medicine
381	medicine_v4<br>Show the field ONLY if:<br>[healthcentre_v4] = '1'	Did the child receive medicine for malaria?	yesno<br>1 Yes<br>0 No
382	med_date_v4<br>Show the field ONLY if:	When did the child take the last dose (i.e. pill) of medicine?<br>*DD-MM-YYYY*	text (date_dmy)

	[medicine_v4] = '1'		
383	mrdt_v4	Section Header: *III. Laboratory Testing - Child*<br>Malaria RDT performed?	yesno, Required<br>1 Yes<br>0 No
384	mrdt_res_v4<br>Show the field ONLY if:<br>[mrdt_v4] = '1'	Malaria RDT Result<br>*Repeat any invalid tests*	radio<br>0 Negative<br>1 Positive
385	treat_v4<br>Show the field ONLY if:<br>([temp_v4] >= 37.5 or [fever_v4] = '1') and [mrdt_res_v4] = '1'	If mother reported fever or child's temperature was >37.5 C, which antimalarial treatment provided?	radio<br>0 None (explain below)<br>1 Coartem<br>2 Quinine<br>3 Admitted
386	no_treat_v4<br>Show the field ONLY if:<br>[treat_v4] = '0'	Why was treatment NOT given?	notes
387	dbs_v4	Dried blood spots collected?	yesno<br>1 Yes<br>0 No
388	lesu_use_v4	Section Header: *IV. Lesu Questions*<br>Since your last visit, how often did you use the lesu to carry the child?	radio, Required<br>0 Never<br>1 Some days (1 - 3 per week)<br>2 Most days (4 - 6 per week)<br>3 Every day
389	washing_v4	Since your last visit, how many times did you wash the lesu?	text (integer, Min: 0, Max: 50), Required
390	se_v4	Did the child experience any side effects, to include itching or rash, from the lesu?	yesno, Required<br>1 Yes<br>0 No
391	se_symp_v4<br>Show the field ONLY if:<br>[se_v4] = '1'	If yes, what were the child's side effects?	checkbox<br>0 se_symp_v4___0 Headache<br>1 se_symp_v4___1 Itching<br>2 se_symp_v4___2 Nausea or not feeding<br>3 se_symp_v4___3 Rash<br>4 se_symp_v4___4 Other
392	se_other_v4<br>Show the field ONLY if:<br>[se_symp_v4(4)] = '1'	Describe the child's other symptoms:	notes
393	se_impact_v4<br>Show the field ONLY if:<br>[se_v4] = '1'	Did the side effects make you stop using the lesu or use the lesu less frequently?	radio<br>0 No change in use<br>1 Used it less frequently<br>2 Stopped using it
394	se_mot_v4	Did the mother experience any side effects, to include itching or rash, from the lesu?	yesno, Required<br>1 Yes<br>0 No
395	se_symp_mot_v4	If yes, what were the side effects?	checkbox

	Show the field ONLY if: [se_mot_v4] = '1'		0 se_symp_mot_v4___0 Headache<br>1 se_symp_mot_v4___1 Itching<br>2 se_symp_mot_v4___2 Nausea or loss of appetite<br>3 se_symp_mot_v4___3 Rash<br>4 se_symp_mot_v4___4 Other
396	se_mot_other_v4<br>Show the field ONLY if: [se_symp_mot_v4(4)] = '1'	Describe other symptoms:	notes
397	se_impact_mot_v4<br>Show the field ONLY if: [se_mot_v4] = '1'	Did these side effects make you stop using the lesu or use the lesu less frequently?	radio<br>0 No change in use<br>1 Used it less frequently<br>2 Stopped using it
398	diary_lesu1_v4	Section Header: *V. Lesu Diary*<br>How many days did the participant report using LESU #1<br>*Enter number between 0 to 14*	text (integer, Min: 0, Max: 14)
399	diary_lesu2_v4	How many days did the participant report using LESU #2<br>*Enter number between 0 to 14*	text (integer, Min: 0, Max: 14)
400	diary_llin_mom_v4	How many nights did the MOTHER report sleeping under a bed net?<br>*Enter number between 0 to 14*	text (integer, Min: 0, Max: 14)
401	diary_llin_child_v4	How many nights did the CHILD report sleeping under a bed net?<br>*Enter number between 0 to 14*	text (integer, Min: 0, Max: 14)
402	diary_carry_v4	How many days was a lesu used to CARRY the child?<br>*Enter number between 0 to 14*	text (integer, Min: 0, Max: 14)
403	diary_sit_v4	How many days was a lesu used as a place for the child to SIT?<br>*Enter number between 0 to 14*	text (integer, Min: 0, Max: 14)
404	diary_sleep_v4	How many days was a lesu used as a BLANKET when the child was put to sleep?<br>*Enter number between 0 to 14*	text (integer, Min: 0, Max: 14)
405	diary_wash_v4	How many days was LESU #1 WASHED?<br>*Enter number between 0 to 14*	text (integer, Min: 0, Max: 14)
406	diary_wash2_v4	How many days was LESU #2 WASHED?<br>*Enter number between 0 to 14*	text (integer, Min: 0, Max: 14)
407	dc_v4	Section Header: *VI. Discharge Actions*<br>Prior to discharge, ensure the following are complete:	checkbox<br>1 dc_v4___1 Retreat lesu according to assignment<br>2 dc_v4___2 Instruct to return to clinic with card if child sick<br>3 dc_v4___3 Remind about next scheduled visit in 2 weeks
408	week_4_clinic_visit_complete	Section Header: *Form Status*<br>Complete?	dropdown<br>0 Incomplete<br>1 Unverified<br>2 Complete
**Instrument: 12. Week 6 Clinic Visit (week_6_clinic_visit)**			
409	date_visit_v6	Section Header: *Week 6 Visit*<br>Date of visit<br>*DD-MM-YYYY*	text (date_dmy), Required
410	temp_v6	Section Header: *I. Vital Signs - Child*	text (number, Min: 35, Max: 45), Required

		Axillary temperature<br>*degrees Celsius*	
411	bednet_v6	Section Header: *II. Medical History - Child*<br>Did the child sleep under a bed net last night?	yesno, Required<br>1 Yes<br>0 No
412	fever_v6	Has the child had fever in last two weeks?	yesno, Required<br>1 Yes<br>0 No
413	onset_v6<br>Show the field ONLY if:<br>[fever_v6] = '1'	If yes, when did the fever start<br>*DD-MM-YYYY*	text (date_dmy)
414	sick_v6<br>Show the field ONLY if:<br>[fever_v6] = '0' OR [fever_v6] = '1'	Even if the child has not had a fever, has he or she been otherwise unwell?	yesno, Required<br>1 Yes<br>0 No
415	symp_v6<br>Show the field ONLY if:<br>[sick_v6] = '1'	If yes, what symptoms has the child experienced?	checkbox<br>0 symp_v6___0 Cough<br>1 symp_v6___1 Diarrhea<br>2 symp_v6___2 Ear Ache<br>3 symp_v6___3 Not feeding<br>4 symp_v6___4 Rash<br>5 symp_v6___5 Runny nose<br>6 symp_v6___6 Other (specify below)
416	symp_other_v6<br>Show the field ONLY if:<br>[symp_v6(6)] = '1'	List other symptoms:	notes
417	healthcentre_v6<br>Show the field ONLY if:<br>[fever_v6] = '1' or [sick_v6] = ' 1'	Has the child been seen at a hospital, health centre, clinic, drug shop, or other medical attendant for these symptoms?	yesno<br>1 Yes<br>0 No
418	hc_where_v6<br>Show the field ONLY if:<br>[healthcentre_v6] = '1'	If yes, where?	radio<br>0 Hospital<br>1 Health Centre<br>2 Drug Shop or Pharmacy<br>3 VHT<br>4 Traditional Medicine
419	medicine_v6<br>Show the field ONLY if:<br>[healthcentre_v6] = '1'	Did the child receive medicine for malaria?	yesno<br>1 Yes<br>0 No
420	med_date_v6<br>Show the field ONLY if:<br>[medicine_v6] = '1'	When did the child take the last dose (i.e. pill) of medicine?<br>*DD-MM-YYYY*	text (date_mdy)
421	mrdt_v6	Section Header: *III. Laboratory Testing - Child*<br>Malaria RDT performed?	yesno, Required<br>1 Yes<br>0 No
422	mrdt_res_v6	Malaria RDT Result	radio

	Show the field ONLY if:<br>[mrdt_v6] = '1'	*Repeat any invalid tests*	0 Negative<br>1 Positive
423	treat_v6<br><br>Show the field ONLY if:<br>([temp_v6] >= 37.5 or [fever_v6] = '1') and [mrdt_res_v6] = '1'	If mother reported fever or child's temperature was >37.5 C, which antimalarial treatment provided?	radio<br>0 None (explain below)<br>1 Coartem<br>2 Quinine<br>3 Admitted
424	no_treat_v6<br><br>Show the field ONLY if:<br>[treat_v6] = '0'	Why was treatment NOT given?	notes
425	dbs_v6	Dried blood spots collected?	yesno<br>1 Yes<br>0 No
426	lesu_use_v6	Section Header: *IV. Lesu Questions*<br>Since your last visit, how often did you use the lesu to carry the child?	radio, Required<br>0 Never<br>1 Some days (1 - 3 per week)<br>2 Most days (4 - 6 per week)<br>3 Every day
427	washing_v6	Since your last visit, how many times did you wash the lesu?	text (integer, Min: 0, Max: 50), Required
428	se_v6	Did the child experience any side effects, to include itching or rash, from the lesu?	yesno, Required<br>1 Yes<br>0 No
429	se_symp_v6<br><br>Show the field ONLY if:<br>[se_v6] = '1'	If yes, what were the child's side effects?	checkbox<br>0 se_symp_v6___0 Headache<br>1 se_symp_v6___1 Itching<br>2 se_symp_v6___2 Nausea or not feeding<br>3 se_symp_v6___3 Rash<br>4 se_symp_v6___4 Other
430	se_other_v6<br><br>Show the field ONLY if:<br>[se_symp_v6(4)] = '1'	Describe the child's other symptoms:	notes
431	se_impact_v6<br><br>Show the field ONLY if:<br>[se_v6] = '1'	Did the side effects make you stop using the lesu or use the lesu less frequently?	radio<br>0 No change in use<br>1 Used it less frequently<br>2 Stopped using it
432	se_mot_v6	Did the mother experience any side effects, to include itching or rash, from the lesu?	yesno, Required<br>1 Yes<br>0 No
433	se_symp_mot_v6<br><br>Show the field ONLY if:<br>[se_mot_v6] = '1'	If yes, what were the side effects?	checkbox<br>0 se_symp_mot_v6___0 Headache<br>1 se_symp_mot_v6___1 Itching<br>2 se_symp_mot_v6___2 Nausea or loss of appetite<br>3 se_symp_mot_v6___3 Rash

			4 | se_symp_mot_v6___4 | Other
434	se_mot_other_v6<br>Show the field ONLY if:<br>[se_symp_mot_v6(4)] = '1'	Describe other symptoms:	notes
435	se_impact_mot_v6<br>Show the field ONLY if:<br>[se_mot_v6] = '1'	Did these side effects make you stop using the lesu or use the lesu less frequently?	radio<br>0 | No change in use<br>1 | Used it less frequently<br>2 | Stopped using it
436	diary_lesu1_v6	Section Header: *V. Lesu Diary*<br>How many days did the participant report using LESU #1<br>*Enter number between 0 to 14*	text (integer, Min: 0, Max: 14)
437	diary_lesu2_v6	How many days did the participant report using LESU #2<br>*Enter number between 0 to 14*	text (integer, Min: 0, Max: 14)
438	diary_llin_mom_v6	How many nights did the MOTHER report sleeping under a bed net?<br>*Enter number between 0 to 14*	text (integer, Min: 0, Max: 14)
439	diary_llin_child_v6	How many nights did the CHILD report sleeping under a bed net?<br>*Enter number between 0 to 14*	text (integer, Min: 0, Max: 14)
440	diary_carry_v6	How many days was a lesu used to CARRY the child?<br>*Enter number between 0 to 14*	text (integer, Min: 0, Max: 14)
441	diary_sit_v6	How many days was a lesu used as a place for the child to SIT?<br>*Enter number between 0 to 14*	text (integer, Min: 0, Max: 14)
442	diary_sleep_v6	How many days was a lesu used as a BLANKET when the child was put to sleep?<br>*Enter number between 0 to 14*	text (integer, Min: 0, Max: 14)
443	diary_wash_v6	How many days was LESU #1 WASHED?<br>*Enter number between 0 to 14*	text (integer, Min: 0, Max: 14)
444	diary_wash2_v6	How many days was LESU #2 WASHED?<br>*Enter number between 0 to 14*	text (integer, Min: 0, Max: 14)
445	dc_v6	Section Header: *VI. Discharge Actions*<br>Prior to discharge, ensure the following are complete:	checkbox<br>2 | dc_v6___2 | Instruct to return to clinic with card if child sick<br>3 | dc_v6___3 | Remind about next scheduled visit in 2 weeks
446	week_6_clinic_visit_complete	Section Header: *Form Status*<br>Complete?	dropdown<br>0 | Incomplete<br>1 | Unverified<br>2 | Complete
**Instrument: 13. Week 8 Clinic Visit (week_8_clinic_visit)**			
447	date_visit_v8	Section Header: *Week 8 Visit*<br>Date of visit<br>*DD-MM-YYYY*	text (date_dmy), Required
448	temp_v8	Section Header: *I. Vital Signs - Child*<br>Axillary temperature<br>*degrees Celsius*	text (number, Min: 35, Max: 45), Required
449	bednet_v8	Section Header: *II. Medical History - Child*<br>Did the child sleep under a bed net last night?	yesno, Required<br>1 | Yes<br>0 | No
450	fever_v8	Has the child had fever in last two weeks?	yesno, Required

			1 Yes<br>0 No
451	onset_v8<br>Show the field ONLY if:<br>[fever_v8] = '1'	If yes, when did the fever start<br>*DD-MM-YYYY*	text (date_dmy)
452	sick_v8<br>Show the field ONLY if:<br>[fever_v8] = '0' OR [fever_v8] = '1'	Even if the child has not had a fever, has he or she been otherwise unwell?	yesno, Required<br>1 Yes<br>0 No
453	symp_v8<br>Show the field ONLY if:<br>[sick_v8] = '1'	If yes, what symptoms has the child experienced?	checkbox<br>0 symp_v8___0 Cough<br>1 symp_v8___1 Diarrhea<br>2 symp_v8___2 Ear Ache<br>3 symp_v8___3 Not feeding<br>4 symp_v8___4 Rash<br>5 symp_v8___5 Runny nose<br>6 symp_v8___6 Other (specify below)
454	symp_other_v8<br>Show the field ONLY if:<br>[symp_v8(6)] = '1'	List other symptoms:	notes
455	healthcentre_v8<br>Show the field ONLY if:<br>[fever_v8] = '1' or [sick_v8] = '1'	Has the child been seen at a hospital, health centre, clinic, drug shop, or other medical attendant for these symptoms?	yesno<br>1 Yes<br>0 No
456	hc_where_v8<br>Show the field ONLY if:<br>[healthcentre_v8] = '1'	If yes, where?	radio<br>0 Hospital<br>1 Health Centre<br>2 Drug Shop or Pharmacy<br>3 VHT<br>4 Traditional Medicine
457	medicine_v8<br>Show the field ONLY if:<br>[healthcentre_v8] = '1'	Did the child receive medicine for malaria?	yesno<br>1 Yes<br>0 No
458	med_date_v8<br>Show the field ONLY if:<br>[medicine_v8] = '1'	When did the child take the last dose (i.e. pill) of medicine?<br>*DD-MM-YYYY*	text (date_dmy)
459	mrdt_v8	Section Header: *III. Laboratory Testing - Child*<br>Malaria RDT performed?	yesno, Required<br>1 Yes<br>0 No
460	mrdt_res_v8<br>Show the field ONLY if:<br>[mrdt_v8] = '1'	Malaria RDT Result<br>*Repeat any invalid tests*	radio<br>0 Negative<br>1 Positive
461	treat_v8<br>Show the field ONLY if:<br>([temp_v8] >= 37.5 or [fever_v8] = '1') and [mrdt_res_v8] = '1	If mother reported fever or child's temperature was >37.5 C, which antimalarial treatment provided?	radio<br>0 None (explain below)<br>1 Coartem

	'		2 Quinine<br>3 Admitted
462	no_treat_v8<br>Show the field ONLY if:<br>[treat_v8] = '0'	Why was treatment NOT given?	notes
463	dbs_v8	Dried blood spots collected?	yesno<br>1 Yes<br>0 No
464	lesu_use_v8	Section Header: *IV. Lesu Questions*<br>Since your last visit, how often did you use the lesu to carry the child?	radio, Required<br>0 Never<br>1 Some days (1 - 3 per week)<br>2 Most days (4 - 6 per week)<br>3 Every day
465	washing_v8	Since your last visit, how many times did you wash the lesu?	text (integer, Min: 0, Max: 50), Required
466	se_v8	Did the child experience any side effects, to include itching or rash, from the lesu?	yesno, Required<br>1 Yes<br>0 No
467	se_symp_v8<br>Show the field ONLY if:<br>[se_v8] = '1'	If yes, what were the child's side effects?	checkbox<br>0 se_symp_v8___0 Headache<br>1 se_symp_v8___1 Itching<br>2 se_symp_v8___2 Nausea or not feeding<br>3 se_symp_v8___3 Rash<br>4 se_symp_v8___4 Other
468	se_other_v8<br>Show the field ONLY if:<br>[se_symp_v8(4)] = '1'	Describe the child's other symptoms:	notes
469	se_impact_v8<br>Show the field ONLY if:<br>[se_v8] = '1'	Did the side effects make you stop using the lesu or use the lesu less frequently?	radio<br>0 No change in use<br>1 Used it less frequently<br>2 Stopped using it
470	se_mot_v8	Did the mother experience any side effects, to include itching or rash, from the lesu?	yesno, Required<br>1 Yes<br>0 No
471	se_symp_mot_v8<br>Show the field ONLY if:<br>[se_mot_v8] = '1'	If yes, what were the side effects?	checkbox<br>0 se_symp_mot_v8___0 Headache<br>1 se_symp_mot_v8___1 Itching<br>2 se_symp_mot_v8___2 Nausea or loss of appetite<br>3 se_symp_mot_v8___3 Rash<br>4 se_symp_mot_v8___4 Other
472	se_mot_other_v8<br>Show the field ONLY if:<br>[se_symp_mot_v8(4)] = '1'	Describe other symptoms:	notes
473	se_impact_mot_v8	Did these side effects make you stop using the lesu or use the	radio

	Show the field ONLY if: [se_mot_v8] = '1'	lesu less frequently?	0 No change in use<br>1 Used it less frequently<br>2 Stopped using it
474	diary_lesu1_v8	Section Header: *V. Lesu Diary*<br>How many days did the participant report using LESU #1<br>*Enter number between 0 to 14*	text (integer, Min: 0, Max: 14)
475	diary_lesu2_v8	How many days did the participant report using LESU #2<br>*Enter number between 0 to 14*	text (integer, Min: 0, Max: 14)
476	diary_llin_mom_v8	How many nights did the MOTHER report sleeping under a bed net?<br>*Enter number between 0 to 14*	text (integer, Min: 0, Max: 14)
477	diary_llin_child_v8	How many nights did the CHILD report sleeping under a bed net?<br>*Enter number between 0 to 14*	text (integer, Min: 0, Max: 14)
478	diary_carry_v8	How many days was a lesu used to CARRY the child?<br>*Enter number between 0 to 14*	text (integer, Min: 0, Max: 14)
479	diary_sit_v8	How many days was a lesu used as a place for the child to SIT?<br>*Enter number between 0 to 14*	text (integer, Min: 0, Max: 14)
480	diary_sleep_v8	How many days was a lesu used as a BLANKET when the child was put to sleep?<br>*Enter number between 0 to 14*	text (integer, Min: 0, Max: 14)
481	diary_wash_v8	How many days was LESU #1 WASHED?<br>*Enter number between 0 to 14*	text (integer, Min: 0, Max: 14)
482	diary_wash2_v8	How many days was LESU #2 WASHED?<br>*Enter number between 0 to 14*	text (integer, Min: 0, Max: 14)
483	dc_v8	Section Header: *VI. Discharge Actions*<br>Prior to discharge, ensure the following are complete:	checkbox<br>1 dc_v8___1 Retreat lesu according to assignment<br>2 dc_v8___2 Instruct to return to clinic with card if child sick<br>3 dc_v8___3 Remind about next scheduled visit in 2 weeks
484	week_8_clinic_visit_complete	Section Header: *Form Status*<br>Complete?	dropdown<br>0 Incomplete<br>1 Unverified<br>2 Complete
**Instrument: 14. Week 10 Clinic Visit (week_10_clinic_visit)**			
485	date_visit_v10	Section Header: *Week 10 Visit*<br>Date of visit<br>*DD-MM-YYYY*	text (date_dmy), Required
486	temp_v10	Section Header: *I. Vital Signs - Child*<br>Axillary temperature<br>*degrees Celsius*	text (number, Min: 35, Max: 45), Required
487	bednet_v10	Section Header: *II. Medical History - Child*<br>Did the child sleep under a bed net last night?	yesno, Required<br>1 Yes<br>0 No
488	fever_v10	Has the child had fever in last two weeks?	yesno, Required<br>1 Yes<br>0 No
489	onset_v10<br>Show the field ONLY if:	If yes, when did the fever start<br>*DD-MM-YYYY*	text (date_dmy)

	[fever_v10] = '1'		
490	sick_v10<br><br>Show the field ONLY if:<br>[fever_v10] = '0' OR [fever_v10] = '1'	Even if the child has not had a fever, has he or she been otherwise unwell?	yesno, Required<br>1 Yes<br>0 No
491	symp_v10<br><br>Show the field ONLY if:<br>[sick_v10] = '1'	If yes, what symptoms has the child experienced?	checkbox<br>0 symp_v10___0 Cough<br>1 symp_v10___1 Diarrhea<br>2 symp_v10___2 Ear Ache<br>3 symp_v10___3 Not feeding<br>4 symp_v10___4 Rash<br>5 symp_v10___5 Runny nose<br>6 symp_v10___6 Other (specify below)
492	symp_other_v10<br><br>Show the field ONLY if:<br>[symp_v10(6)] = '1'	List other symptoms:	notes
493	healthcentre_v10<br><br>Show the field ONLY if:<br>[fever_v10] = '1' or [sick_v10] = '1'	Has the child been seen at a hospital, health centre, clinic, drug shop, or other medical attendant for these symptoms?	yesno<br>1 Yes<br>0 No
494	hc_where_v10<br><br>Show the field ONLY if:<br>[healthcentre_v10] = '1'	If yes, where?	radio<br>0 Hospital<br>1 Health Centre<br>2 Drug Shop or Pharmacy<br>3 VHT<br>4 Traditional Medicine
495	medicine_v10<br><br>Show the field ONLY if:<br>[healthcentre_v10] = '1'	Did the child receive medicine for malaria?	yesno<br>1 Yes<br>0 No
496	med_date_v10<br><br>Show the field ONLY if:<br>[medicine_v10] = '1'	When did the child take the last dose (i.e. pill) of medicine?<br>*DD-MM-YYYY*	text (date_dmy)
497	mrdt_v10	Section Header: *III. Laboratory Testing - Child*<br>Malaria RDT performed?	yesno, Required<br>1 Yes<br>0 No
498	mrdt_res_v10<br><br>Show the field ONLY if:<br>[mrdt_v10] = '1'	Malaria RDT Result<br>*Repeat any invalid tests*	radio<br>0 Negative<br>1 Positive
499	treat_v10<br><br>Show the field ONLY if:<br>([temp_v10] >= 37.5 or [fever_v10] = '1') and [mrdt_res_v10] = '1'	If mother reported fever or child's temperature was >37.5 C, which antimalarial treatment provided?	radio<br>0 None (explain below)<br>1 Coartem<br>2 Quinine<br>3 Admitted
500	no_treat_v10<br><br>Show the field ONLY if:<br>[treat_v10] = '0'	Why was treatment NOT given?	notes

501	dbs_v10	Dried blood spots collected?	yesno<br>1 Yes<br>0 No
502	lesu_use_v10	Section Header: *IV. Lesu Questions*<br>Since your last visit, how often did you use the lesu to carry the child?	radio, Required<br>0 Never<br>1 Some days (1 - 3 per week)<br>2 Most days (4 - 6 per week)<br>3 Every day
503	washing_v10	Since your last visit, how many times did you wash the lesu?	text (integer, Min: 0, Max: 50), Required
504	se_v10	Did the child experience any side effects, to include itching or rash, from the lesu?	yesno, Required<br>1 Yes<br>0 No
505	se_symp_v10<br>Show the field ONLY if:<br>[se_v10] = '1'	If yes, what were the child's side effects?	checkbox<br>0 se_symp_v10___0 Headache<br>1 se_symp_v10___1 Itching<br>2 se_symp_v10___2 Nausea or not feeding<br>3 se_symp_v10___3 Rash<br>4 se_symp_v10___4 Other
506	se_other_v10<br>Show the field ONLY if:<br>[se_symp_v10(4)] = '1'	Describe the child's other symptoms:	notes
507	se_impact_v10<br>Show the field ONLY if:<br>[se_v10] = '1'	Did the side effects make you stop using the lesu or use the lesu less frequently?	radio<br>0 No change in use<br>1 Used it less frequently<br>2 Stopped using it
508	se_mot_v10	Did the mother experience any side effects, to include itching or rash, from the lesu?	yesno, Required<br>1 Yes<br>0 No
509	se_symp_mot_v10<br>Show the field ONLY if:<br>[se_mot_v10] = '1'	If yes, what were the side effects?	checkbox<br>0 se_symp_mot_v10___0 Headache<br>1 se_symp_mot_v10___1 Itching<br>2 se_symp_mot_v10___2 Nausea or loss of appetite<br>3 se_symp_mot_v10___3 Rash<br>4 se_symp_mot_v10___4 Other
510	se_mot_other_v10<br>Show the field ONLY if:<br>[se_symp_mot_v10(4)] = '1'	Describe other symptoms:	notes
511	se_impact_mot_v10<br>Show the field ONLY if:<br>[se_mot_v10] = '1'	Did these side effects make you stop using the lesu or use the lesu less frequently?	radio<br>0 No change in use<br>1 Used it less frequently<br>2 Stopped using it
512	diary_lesu1_v10	Section Header: *V. Lesu Diary*<br>How many days did the participant report using LESU #1	text (integer, Min: 0, Max: 14)

		Enter number between 0 to 14	
513	diary_lesu2_v10	How many days did the participant report using LESU #2<br>*Enter number between 0 to 14*	text (integer, Min: 0, Max: 14)
514	diary_llin_mom_v10	How many nights did the MOTHER report sleeping under a bed net?<br>*Enter number between 0 to 14*	text (integer, Min: 0, Max: 14)
515	diary_llin_child_v10	How many nights did the CHILD report sleeping under a bed net?<br>*Enter number between 0 to 14*	text (integer, Min: 0, Max: 14)
516	diary_carry_v10	How many days was a lesu used to CARRY the child?<br>*Enter number between 0 to 14*	text (integer, Min: 0, Max: 14)
517	diary_sit_v10	How many days was a lesu used as a place for the child to SIT?<br>*Enter number between 0 to 14*	text (integer, Min: 0, Max: 14)
518	diary_sleep_v10	How many days was a lesu used as a BLANKET when the child was put to sleep?<br>*Enter number between 0 to 14*	text (integer, Min: 0, Max: 14)
519	diary_wash_v10	How many days was LESU #1 WASHED?<br>*Enter number between 0 to 14*	text (integer, Min: 0, Max: 14)
520	diary_wash2_v10	How many days was LESU #2 WASHED?<br>*Enter number between 0 to 14*	text (integer, Min: 0, Max: 14)
521	dc_v10	Section Header: *VI. Discharge Actions*<br>Prior to discharge, ensure the following are complete:	checkbox<br>2 | dc_v10___2 | Instruct to return to clinic with card if child sick<br>3 | dc_v10___3 | Remind about next scheduled visit in 2 weeks
522	week_10_clinic_visit_complete	Section Header: *Form Status*<br>Complete?	dropdown<br>0 | Incomplete<br>1 | Unverified<br>2 | Complete
**Instrument: 15. Week 12 Clinic Visit (week_12_clinic_visit)**			
523	date_visit_v12	Section Header: *Week 12 (Midpoint) Visit*<br>Date of visit<br>*DD-MM-YYYY*	text (date_dmy), Required
524	height_v12	Section Header: *I. Vital Signs - Child*<br>Height<br>*cm*	text (number, Min: 50, Max: 100), Required
525	weight_v12	Weight<br>*kg*	text (number, Min: 5, Max: 20), Required
526	muac_v12	Mid-Upper Arm Circumference<br>*cm*	text (number, Min: 5, Max: 25), Required
527	temp_v12	Axillary temperature<br>*degrees Celsius*	text (number, Min: 35, Max: 45), Required
528	bednet_v12	Section Header: *II. Medical History - Child*<br>Did the child sleep under a bed net last night?	yesno, Required<br>1 | Yes<br>0 | No
529	fever_v12	Has the child had fever in last two weeks?	yesno, Required<br>1 | Yes<br>0 | No
530	onset_v12<br>Show the field ONLY if:<br>[fever_v12] = '1'	If yes, when did the fever start<br>*DD-MM-YYYY*	text (date_dmy)

531	sick_v12<br>Show the field ONLY if:<br>[fever_v12] = '0' OR [fever_v12] = '1'	Even if the child has not had a fever, has he or she been otherwise unwell?	yesno, Required<br>1 Yes<br>0 No
532	symp_v12<br>Show the field ONLY if:<br>[sick_v12] = '1'	If yes, what symptoms has the child experienced?	checkbox<br>0 symp_v12___0 Cough<br>1 symp_v12___1 Diarrhea<br>2 symp_v12___2 Ear Ache<br>3 symp_v12___3 Not feeding<br>4 symp_v12___4 Rash<br>5 symp_v12___5 Runny nose<br>6 symp_v12___6 Other (specify below)
533	symp_other_v12<br>Show the field ONLY if:<br>[symp_v12(6)] = '1'	List other symptoms:	notes
534	healthcentre_v12<br>Show the field ONLY if:<br>[fever_v12] = '1' or [sick_v12] = '1'	Has the child been seen at a hospital, health centre, clinic, drug shop, or other medical attendant for these symptoms?	yesno<br>1 Yes<br>0 No
535	hc_where_v12<br>Show the field ONLY if:<br>[healthcentre_v12] = '1'	If yes, where?	radio<br>0 Hospital<br>1 Health Centre<br>2 Drug Shop or Pharmacy<br>3 VHT<br>4 Traditional Medicine
536	medicine_v12<br>Show the field ONLY if:<br>[healthcentre_v12] = '1'	Did the child receive medicine for malaria?	yesno<br>1 Yes<br>0 No
537	med_date_v12<br>Show the field ONLY if:<br>[medicine_v12] = '1'	When did the child take the last dose (i.e. pill) of medicine?<br>*DD-MM-YYYY*	text (date_dmy)
538	mrdt_v12	Section Header: *III. Laboratory Testing - Child*<br>Malaria RDT performed?	yesno, Required<br>1 Yes<br>0 No
539	mrdt_res_v12<br>Show the field ONLY if:<br>[mrdt_v12] = '1'	Malaria RDT Result<br>*Repeat any invalid tests*	radio<br>0 Negative<br>1 Positive
540	treat_v12<br>Show the field ONLY if:<br>([temp_v12] >= 37.5 or [fever_v12] = '1') and [mrdt_res_v12] = '1'	If mother reported fever or child's temperature was >37.5 C, which antimalarial treatment provided?	radio<br>0 None (explain below)<br>1 Coartem<br>2 Quinine<br>3 Admitted
541	no_treat_v12<br>Show the field ONLY if:<br>[treat_v12] = '0'	Why was treatment NOT given?	notes
542	cbc_v12	Hemoglobin measured?	yesno

			1 Yes<br>0 No
543	hb_v12<br>Show the field ONLY if:<br>[cbc_v12] = '1'	Hemoglobin (g/dL)	text (number, Min: 3, Max: 20)
544	dbs_v12	Dried blood spots collected?	yesno<br>1 Yes<br>0 No
545	urine_v12	Urine sample collected?	yesno<br>1 Yes<br>0 No
546	bednet_mot_v12	Section Header: *IV. Medical History - Mother*<br>Did the mother sleep under a bed net last night?	yesno, Required<br>1 Yes<br>0 No
547	cosleep_v12<br>Show the field ONLY if:<br>[bednet_mot_v12] = '1'	Did the mother sleep under the same net as the child?	yesno<br>1 Yes<br>0 No
548	cbc_mot_v12	Section Header: *V. Laboratory Testing - Mother*<br>Hemoglobin measured?	yesno<br>1 Yes<br>0 No
549	hb_mot_v12<br>Show the field ONLY if:<br>[cbc_mot_v12] = '1'	Hemoglobin (g/dL)	text (number, Min: 3, Max: 20)
550	dbs_mot_v12	Dried blood spots collected?	yesno<br>1 Yes<br>0 No
551	urine_mot_v12	Urine sample collected?	yesno<br>1 Yes<br>0 No
552	lesu_use_v12	Section Header: *VI. Lesu Questions*<br>Since your last visit, how often did you use the lesu to carry the child?	radio, Required<br>0 Never<br>1 Some days (1 - 3 per week)<br>2 Most days (4 - 6 per week)<br>3 Every day
553	washing_v12	Since your last visit, how many times did you wash the lesu?	text (integer, Min: 0, Max: 50), Required
554	se_v12	Did the child experience any side effects, to include itching or rash, from the lesu?	yesno, Required<br>1 Yes<br>0 No
555	se_symp_v12<br>Show the field ONLY if:<br>[se_v12] = '1'	If yes, what were the child's side effects?	checkbox<br>0 se_symp_v12___0 Headache<br>1 se_symp_v12___1 Itching<br>2 se_symp_v12___2 Nausea or not feeding<br>3 se_symp_v12___3 Rash

			4 se_symp_v12___4 Other
556	se_other_v12<br>Show the field ONLY if:<br>[se_symp_v12(4)] = '1'	Describe the child's other symptoms:	notes
557	se_impact_v12<br>Show the field ONLY if:<br>[se_v12] = '1'	Did the side effects make you stop using the lesu or use the lesu less frequently?	radio<br>0 No change in use<br>1 Used it less frequently<br>2 Stopped using it
558	se_mot_v12	Did the mother experience any side effects, to include itching or rash, from the lesu?	yesno, Required<br>1 Yes<br>0 No
559	se_symp_mot_v12<br>Show the field ONLY if:<br>[se_mot_v12] = '1'	If yes, what were the side effects?	checkbox<br>0 se_symp_mot_v12___0 Headache<br>1 se_symp_mot_v12___1 Itching<br>2 se_symp_mot_v12___2 Nausea or loss of appetite<br>3 se_symp_mot_v12___3 Rash<br>4 se_symp_mot_v12___4 Other
560	se_mot_other_v12<br>Show the field ONLY if:<br>[se_symp_mot_v12(4)] = '1'	Describe other symptoms:	notes
561	se_impact_mot_v12<br>Show the field ONLY if:<br>[se_mot_v12] = '1'	Did these side effects make you stop using the lesu or use the lesu less frequently?	radio<br>0 No change in use<br>1 Used it less frequently<br>2 Stopped using it
562	diary_lesu1_v12	Section Header: *V. Lesu Diary*<br>How many days did the participant report using LESU #1<br>*Enter number between 0 to 14*	text (integer, Min: 0, Max: 14)
563	diary_lesu2_v12	How many days did the participant report using LESU #2<br>*Enter number between 0 to 14*	text (integer, Min: 0, Max: 14)
564	diary_llin_mom_v12	How many nights did the MOTHER report sleeping under a bed net?<br>*Enter number between 0 to 14*	text (integer, Min: 0, Max: 14)
565	diary_llin_child_v12	How many nights did the CHILD report sleeping under a bed net?<br>*Enter number between 0 to 14*	text (integer, Min: 0, Max: 14)
566	diary_carry_v12	How many days was a lesu used to CARRY the child?<br>*Enter number between 0 to 14*	text (integer, Min: 0, Max: 14)
567	diary_sit_v12	How many days was a lesu used as a place for the child to SIT?<br>*Enter number between 0 to 14*	text (integer, Min: 0, Max: 14)
568	diary_sleep_v12	How many days was a lesu used as a BLANKET when the child was put to sleep?<br>*Enter number between 0 to 14*	text (integer, Min: 0, Max: 14)
569	diary_wash_v12	How many days was LESU #1 WASHED?<br>*Enter number between 0 to 14*	text (integer, Min: 0, Max: 14)
570	diary_wash2_v12	How many days was LESU #2 WASHED?<br>*Enter number between 0 to 14*	text (integer, Min: 0, Max: 14)
571	dc_v12	Section Header: *VII. Discharge Actions*<br>Prior to discharge, ensure the following are complete:	checkbox<br>1 dc_v12___1 Obtain punch from lesu<br>2 dc_v12___2 Retreat lesu according to

			(continued) assignment<br>3 | dc_v12___3 | Instruct to return to clinic with card if child sick<br>4 | dc_v12___4 | Remind about next scheduled visit in 2 weeks
572	week_12_clinic_visit_complete	Section Header: *Form Status*<br>Complete?	dropdown<br>0 | Incomplete<br>1 | Unverified<br>2 | Complete
**Instrument: 16. Week 14 Clinic Visit (week_14_clinic_visit)**			
573	date_visit_v14	Section Header: *Week 14 Visit*<br>Date of visit<br>*DD-MM-YYYY*	text (date_dmy), Required
574	temp_v14	Section Header: *I. Vital Signs - Child*<br>Axillary temperature<br>*degrees Celsius*	text (number, Min: 35, Max: 45), Required
575	bednet_v14	Section Header: *II. Medical History - Child*<br>Did the child sleep under a bed net last night?	yesno, Required<br>1 | Yes<br>0 | No
576	fever_v14	Has the child had fever in last two weeks?	yesno, Required<br>1 | Yes<br>0 | No
577	onset_v14<br>Show the field ONLY if:<br>[fever_v14] = '1'	If yes, when did the fever start<br>*DD-MM-YYYY*	text (date_dmy)
578	sick_v14<br>Show the field ONLY if:<br>[fever_v14] = '0' OR [fever_v14] = '1'	Even if the child has not had a fever, has he or she been otherwise unwell?	yesno, Required<br>1 | Yes<br>0 | No
579	symp_v14<br>Show the field ONLY if:<br>[sick_v14] = '1'	If yes, what symptoms has the child experienced?	checkbox<br>0 | symp_v14___0 | Cough<br>1 | symp_v14___1 | Diarrhea<br>2 | symp_v14___2 | Ear Ache<br>3 | symp_v14___3 | Not feeding<br>4 | symp_v14___4 | Rash<br>5 | symp_v14___5 | Runny nose<br>6 | symp_v14___6 | Other (specify below)
580	symp_other_v14<br>Show the field ONLY if:<br>[symp_v14(6)] = '1'	List other symptoms:	notes
581	healthcentre_v14<br>Show the field ONLY if:<br>[fever_v14] = '1' or [sick_v14] = '1'	Has the child been seen at a hospital, health centre, clinic, drug shop, or other medical attendant for these symptoms?	yesno<br>1 | Yes<br>0 | No
582	hc_where_v14<br>Show the field ONLY if:	If yes, where?	radio<br>0 | Hospital

	[healthcentre_v14] = '1'		1 Health Centre<br>2 Drug Shop or Pharmacy<br>3 VHT<br>4 Traditional Medicine
583	medicine_v14<br><br>Show the field ONLY if:<br>[healthcentre_v14] = '1'	Did the child receive medicine for malaria?	yesno<br>1 Yes<br>0 No
584	med_date_v14<br><br>Show the field ONLY if:<br>[medicine_v14] = '1'	When did the child take the last dose (i.e. pill) of medicine?<br>*DD-MM-YYYY*	text (date_dmy)
585	mrdt_v14	Section Header: *III. Laboratory Testing - Child*<br>Malaria RDT performed?	yesno, Required<br>1 Yes<br>0 No
586	mrdt_res_v14<br><br>Show the field ONLY if:<br>[mrdt_v14] = '1'	Malaria RDT Result<br>*Repeat any invalid tests*	radio<br>0 Negative<br>1 Positive
587	treat_v14<br><br>Show the field ONLY if:<br>([temp_v14] >= 37.5 or [fever_v14] = '1') and [mrdt_res_v14] = '1'	If mother reported fever or child's temperature was >37.5 C, which antimalarial treatment provided?	radio<br>0 None (explain below)<br>1 Coartem<br>2 Quinine<br>3 Admitted
588	no_treat_v14<br><br>Show the field ONLY if:<br>[treat_v14] = '0'	Why was treatment NOT given?	notes
589	dbs_v14	Dried blood spots collected?	yesno<br>1 Yes<br>0 No
590	lesu_use_v14	Section Header: *IV. Lesu Questions*<br>Since your last visit, how often did you use the lesu to carry the child?	radio, Required<br>0 Never<br>1 Some days (1 - 3 per week)<br>2 Most days (4 - 6 per week)<br>3 Every day
591	washing_v14	Since your last visit, how many times did you wash the lesu?	text (integer, Min: 0, Max: 50), Required
592	se_v14	Did the child experience any side effects, to include itching or rash, from the lesu?	yesno, Required<br>1 Yes<br>0 No
593	se_symp_v14<br><br>Show the field ONLY if:<br>[se_v14] = '1'	If yes, what were the child's side effects?	checkbox<br>0 se_symp_v14___0 Headache<br>1 se_symp_v14___1 Itching<br>2 se_symp_v14___2 Nausea or not feeding<br>3 se_symp_v14___3 Rash<br>4 se_symp_v14___4 Other
594	se_other_v14	Describe the child's other symptoms:	notes

	Show the field ONLY if:<br>[se_symp_v14(4)] = '1'		
595	se_impact_v14<br><br>Show the field ONLY if:<br>[se_v14] = '1'	Did the side effects make you stop using the lesu or use the lesu less frequently?	radio<br>0 No change in use<br>1 Used it less frequently<br>2 Stopped using it
596	se_mot_v14	Did the mother experience any side effects, to include itching or rash, from the lesu?	yesno, Required<br>1 Yes<br>0 No
597	se_symp_mot_v14<br><br>Show the field ONLY if:<br>[se_mot_v14] = '1'	If yes, what were the side effects?	checkbox<br>0 se_symp_mot_v14___0 Headache<br>1 se_symp_mot_v14___1 Itching<br>2 se_symp_mot_v14___2 Nausea or loss of appetite<br>3 se_symp_mot_v14___3 Rash<br>4 se_symp_mot_v14___4 Other
598	se_mot_other_v14<br><br>Show the field ONLY if:<br>[se_symp_mot_v14(4)] = '1'	Describe other symptoms:	notes
599	se_impact_mot_v14<br><br>Show the field ONLY if:<br>[se_mot_v14] = '1'	Did these side effects make you stop using the lesu or use the lesu less frequently?	radio<br>0 No change in use<br>1 Used it less frequently<br>2 Stopped using it
600	diary_lesu1_v14	Section Header: *V. Lesu Diary*<br>How many days did the participant report using LESU #1<br>*Enter number between 0 to 14*	text (integer, Min: 0, Max: 14)
601	diary_lesu2_v14	How many days did the participant report using LESU #2<br>*Enter number between 0 to 14*	text (integer, Min: 0, Max: 14)
602	diary_llin_mom_v14	How many nights did the MOTHER report sleeping under a bed net?<br>*Enter number between 0 to 14*	text (integer, Min: 0, Max: 14)
603	diary_llin_child_v14	How many nights did the CHILD report sleeping under a bed net?<br>*Enter number between 0 to 14*	text (integer, Min: 0, Max: 14)
604	diary_carry_v14	How many days was a lesu used to CARRY the child?<br>*Enter number between 0 to 14*	text (integer, Min: 0, Max: 14)
605	diary_sit_v14	How many days was a lesu used as a place for the child to SIT?<br>*Enter number between 0 to 14*	text (integer, Min: 0, Max: 14)
606	diary_sleep_v14	How many days was a lesu used as a BLANKET when the child was put to sleep?<br>*Enter number between 0 to 14*	text (integer, Min: 0, Max: 14)
607	diary_wash_v14	How many days was LESU #1 WASHED?<br>*Enter number between 0 to 14*	text (integer, Min: 0, Max: 14)
608	diary_wash2_v14	How many days was LESU #2 WASHED?<br>*Enter number between 0 to 14*	text (integer, Min: 0, Max: 14)
609	dc_v14	Section Header: *VI. Discharge Actions*<br>Prior to discharge, ensure the following are complete:	checkbox<br>2 dc_v14___2 Instruct to return to clinic with card if child sick<br>3 dc_v14___3 Remind about next scheduled visit in 2 weeks

610	week_14_clinic_visit_complete	Section Header: *Form Status*<br>Complete?	dropdown<br>0 Incomplete<br>1 Unverified<br>2 Complete
**Instrument: 17. Week 16 Clinic Visit (week_16_clinic_visit)**			
611	date_visit_v16	Section Header: *Week 16 Visit*<br>Date of visit<br>*DD-MM-YYYY*	text (date_dmy), Required
612	temp_v16	Section Header: *I. Vital Signs - Child*<br>Axillary temperature<br>*degrees Celsius*	text (number, Min: 35, Max: 45), Required
613	bednet_v16	Section Header: *II. Medical History - Child*<br>Did the child sleep under a bed net last night?	yesno, Required<br>1 Yes<br>0 No
614	fever_v16	Has the child had fever in last two weeks?	yesno, Required<br>1 Yes<br>0 No
615	onset_v16<br>Show the field ONLY if:<br>[fever_v16] = '1'	If yes, when did the fever start<br>*DD-MM-YYYY*	text (date_dmy)
616	sick_v16<br>Show the field ONLY if:<br>[fever_v16] = '0' OR [fever_v16] = '1'	Even if the child has not had a fever, has he or she been otherwise unwell?	yesno, Required<br>1 Yes<br>0 No
617	symp_v16<br>Show the field ONLY if:<br>[sick_v16] = '1'	If yes, what symptoms has the child experienced?	checkbox<br>0 symp_v16___0 Cough<br>1 symp_v16___1 Diarrhea<br>2 symp_v16___2 Ear Ache<br>3 symp_v16___3 Not feeding<br>4 symp_v16___4 Rash<br>5 symp_v16___5 Runny nose<br>6 symp_v16___6 Other (specify below)
618	symp_other_v16<br>Show the field ONLY if:<br>[symp_v16(6)] = '1'	List other symptoms:	notes
619	healthcentre_v16<br>Show the field ONLY if:<br>[fever_v16] = '1' or [sick_v16] = '1'	Has the child been seen at a hospital, health centre, clinic, drug shop, or other medical attendant for these symptoms?	yesno<br>1 Yes<br>0 No
620	hc_where_v16<br>Show the field ONLY if:<br>[healthcentre_v16] = '1'	If yes, where?	radio<br>0 Hospital<br>1 Health Centre<br>2 Drug Shop or Pharmacy<br>3 VHT<br>4 Traditional Medicine
621	medicine_v16	Did the child receive medicine for malaria?	yesno<br>1 Yes

	Show the field ONLY if: [healthcentre_v16] = '1'		0 No
622	med_date_v16<br>Show the field ONLY if: [medicine_v16] = '1'	When did the child take the last dose (i.e. pill) of medicine?<br>*DD-MM-YYYY*	text (date_dmy)
623	mrdt_v16	Section Header: *III. Laboratory Testing - Child*<br>Malaria RDT performed?	yesno, Required<br>1 Yes<br>0 No
624	mrdt_res_v16<br>Show the field ONLY if: [mrdt_v16] = '1'	Malaria RDT Result<br>*Repeat any invalid tests*	radio<br>0 Negative<br>1 Positive
625	treat_v16<br>Show the field ONLY if: ([temp_v16] >= 37.5 or [fever_v16] = '1') and [mrdt_res_v16] = '1'	If mother reported fever or child's temperature was >37.5 C, which antimalarial treatment provided?	radio<br>0 None (explain below)<br>1 Coartem<br>2 Quinine<br>3 Admitted
626	no_treat_v16<br>Show the field ONLY if: [treat_v16] = '0'	Why was treatment NOT given?	notes
627	dbs_v16	Dried blood spots collected?	yesno<br>1 Yes<br>0 No
628	lesu_use_v16	Section Header: *IV. Lesu Questions*<br>Since your last visit, how often did you use the lesu to carry the child?	radio, Required<br>0 Never<br>1 Some days (1 - 3 per week)<br>2 Most days (4 - 6 per week)<br>3 Every day
629	washing_v16	Since your last visit, how many times did you wash the lesu?	text (integer, Min: 0, Max: 50), Required
630	se_v16	Did the child experience any side effects, to include itching or rash, from the lesu?	yesno, Required<br>1 Yes<br>0 No
631	se_symp_v16<br>Show the field ONLY if: [se_v16] = '1'	If yes, what were the child's side effects?	checkbox<br>0 se_symp_v16___0 Headache<br>1 se_symp_v16___1 Itching<br>2 se_symp_v16___2 Nausea or not feeding<br>3 se_symp_v16___3 Rash<br>4 se_symp_v16___4 Other
632	se_other_v16<br>Show the field ONLY if: [se_symp_v16(4)] = '1'	Describe the child's other symptoms:	notes
633	se_impact_v16<br>Show the field ONLY if: [se_v16] = '1'	Did the side effects make you stop using the lesu or use the lesu less frequently?	radio<br>0 No change in use<br>1 Used it less frequently<br>2 Stopped using it

634	se_mot_v16	Did the mother experience any side effects, to include itching or rash, from the lesu?	yesno, Required<br>1 Yes<br>0 No
635	se_symp_mot_v16<br>Show the field ONLY if:<br>[se_mot_v16] = '1'	If yes, what were the side effects?	checkbox<br>0 se_symp_mot_v16___0 Headache<br>1 se_symp_mot_v16___1 Itching<br>2 se_symp_mot_v16___2 Nausea or loss of appetite<br>3 se_symp_mot_v16___3 Rash<br>4 se_symp_mot_v16___4 Other
636	se_mot_other_v16<br>Show the field ONLY if:<br>[se_symp_mot_v16(4)] = '1'	Describe other symptoms:	notes
637	se_impact_mot_v16<br>Show the field ONLY if:<br>[se_mot_v16] = '1'	Did these side effects make you stop using the lesu or use the lesu less frequently?	radio<br>0 No change in use<br>1 Used it less frequently<br>2 Stopped using it
638	diary_lesu1_v16	Section Header: *V. Lesu Diary*<br>How many days did the participant report using LESU #1<br>*Enter number between 0 to 14*	text (integer, Min: 0, Max: 14)
639	diary_lesu2_v16	How many days did the participant report using LESU #2<br>*Enter number between 0 to 14*	text (integer, Min: 0, Max: 14)
640	diary_llin_mom_v16	How many nights did the MOTHER report sleeping under a bed net?<br>*Enter number between 0 to 14*	text (integer, Min: 0, Max: 14)
641	diary_llin_child_v16	How many nights did the CHILD report sleeping under a bed net?<br>*Enter number between 0 to 14*	text (integer, Min: 0, Max: 14)
642	diary_carry_v16	How many days was a lesu used to CARRY the child?<br>*Enter number between 0 to 14*	text (integer, Min: 0, Max: 14)
643	diary_sit_v16	How many days was a lesu used as a place for the child to SIT?<br>*Enter number between 0 to 14*	text (integer, Min: 0, Max: 14)
644	diary_sleep_v16	How many days was a lesu used as a BLANKET when the child was put to sleep?<br>*Enter number between 0 to 14*	text (integer, Min: 0, Max: 14)
645	diary_wash_v16	How many days was LESU #1 WASHED?<br>*Enter number between 0 to 14*	text (integer, Min: 0, Max: 14)
646	diary_wash2_v16	How many days was LESU #2 WASHED?<br>*Enter number between 0 to 14*	text (integer, Min: 0, Max: 14)
647	dc_v16	Section Header: *VI. Discharge Actions*<br>Prior to discharge, ensure the following are complete:	checkbox<br>1 dc_v16___1 Retreat lesu according to assignment<br>2 dc_v16___2 Instruct to return to clinic with card if child sick<br>3 dc_v16___3 Remind about next scheduled visit in 2 weeks
648	week_16_clinic_visit_complete	Section Header: *Form Status*<br>Complete?	dropdown<br>0 Incomplete<br>1 Unverified<br>2 Complete

Instrument: 18. Week 18 Clinic Visit (week_18_clinic_visit)

649	date_visit_v18	Section Header: *Week 18 Visit*<br>Date of visit<br>*DD-MM-YYYY*	text (date_dmy), Required
650	temp_v18	Section Header: *I. Vital Signs - Child*<br>Axillary temperature<br>*degrees Celsius*	text (number, Min: 35, Max: 45), Required
651	bednet_v18	Section Header: *II. Medical History - Child*<br>Did the child sleep under a bed net last night?	yesno, Required<br>1 Yes<br>0 No
652	fever_v18	Has the child had fever in last two weeks?	yesno, Required<br>1 Yes<br>0 No
653	onset_v18<br>Show the field ONLY if:<br>[fever_v18] = '1'	If yes, when did the fever start<br>*DD-MM-YYYY*	text (date_dmy)
654	sick_v18<br>Show the field ONLY if:<br>[fever_v18] = '0' OR [fever_v18] = '1'	Even if the child has not had a fever, has he or she been otherwise unwell?	yesno, Required<br>1 Yes<br>0 No
655	symp_v18<br>Show the field ONLY if:<br>[sick_v18] = '1'	If yes, what symptoms has the child experienced?	checkbox<br>0 symp_v18___0 Cough<br>1 symp_v18___1 Diarrhea<br>2 symp_v18___2 Ear Ache<br>3 symp_v18___3 Not feeding<br>4 symp_v18___4 Rash<br>5 symp_v18___5 Runny nose<br>6 symp_v18___6 Other (specify below)
656	symp_other_v18<br>Show the field ONLY if:<br>[symp_v18(6)] = '1'	List other symptoms:	notes
657	healthcentre_v18<br>Show the field ONLY if:<br>[fever_v18] = '1' or [sick_v18] = '1'	Has the child been seen at a hospital, health centre, clinic, drug shop, or other medical attendant for these symptoms?	yesno<br>1 Yes<br>0 No
658	hc_where_v18<br>Show the field ONLY if:<br>[healthcentre_v18] = '1'	If yes, where?	radio<br>0 Hospital<br>1 Health Centre<br>2 Drug Shop or Pharmacy<br>3 VHT<br>4 Traditional Medicine
659	medicine_v18<br>Show the field ONLY if:<br>[healthcentre_v18] = '1'	Did the child receive medicine for malaria?	yesno<br>1 Yes<br>0 No
660	med_date_v18<br>Show the field ONLY if:	When did the child take the last dose (i.e. pill) of medicine?<br>*DD-MM-YYYY*	text (date_dmy)

	[medicine_v18] = '1'		
661	mrdt_v18	Section Header: *III. Laboratory Testing - Child*<br>Malaria RDT performed?	yesno, Required<br>1 Yes<br>0 No
662	mrdt_res_v18<br>Show the field ONLY if:<br>[mrdt_v18] = '1'	Malaria RDT Result<br>*Repeat any invalid tests*	radio<br>0 Negative<br>1 Positive
663	treat_v18<br>Show the field ONLY if:<br>([temp_v18] >= 37.5 or [fever_v18] = '1') and [mrdt_res_v18] = '1'	If mother reported fever or child's temperature was >37.5 C, which antimalarial treatment provided?	radio<br>0 None (explain below)<br>1 Coartem<br>2 Quinine<br>3 Admitted
664	no_treat_v18<br>Show the field ONLY if:<br>[treat_v18] = '0'	Why was treatment NOT given?	notes
665	dbs_v18	Dried blood spots collected?	yesno<br>1 Yes<br>0 No
666	lesu_use_v18	Section Header: *IV. Lesu Questions*<br>Since your last visit, how often did you use the lesu to carry the child?	radio, Required<br>0 Never<br>1 Some days (1 - 3 per week)<br>2 Most days (4 - 6 per week)<br>3 Every day
667	washing_v18	Since your last visit, how many times did you wash the lesu?	text (integer, Min: 0, Max: 50), Required
668	se_v18	Did the child experience any side effects, to include itching or rash, from the lesu?	yesno, Required<br>1 Yes<br>0 No
669	se_symp_v18<br>Show the field ONLY if:<br>[se_v18] = '1'	If yes, what were the child's side effects?	checkbox<br>0 se_symp_v18___0 Headache<br>1 se_symp_v18___1 Itching<br>2 se_symp_v18___2 Nausea or not feeding<br>3 se_symp_v18___3 Rash<br>4 se_symp_v18___4 Other
670	se_other_v18<br>Show the field ONLY if:<br>[se_symp_v18(4)] = '1'	Describe the child's other symptoms:	notes
671	se_impact_v18<br>Show the field ONLY if:<br>[se_v18] = '1'	Did the side effects make you stop using the lesu or use the lesu less frequently?	radio<br>0 No change in use<br>1 Used it less frequently<br>2 Stopped using it
672	se_mot_v18	Did the mother experience any side effects, to include itching or rash, from the lesu?	yesno, Required<br>1 Yes<br>0 No

673	se_symp_mot_v18<br>Show the field ONLY if:<br>[se_mot_v18] = '1'	If yes, what were the side effects?	checkbox<br>0 se_symp_mot_v18___0 Headache<br>1 se_symp_mot_v18___1 Itching<br>2 se_symp_mot_v18___2 Nausea or loss of appetite<br>3 se_symp_mot_v18___3 Rash<br>4 se_symp_mot_v18___4 Other
674	se_mot_other_v18<br>Show the field ONLY if:<br>[se_symp_mot_v18(4)] = '1'	Describe other symptoms:	notes
675	se_impact_mot_v18<br>Show the field ONLY if:<br>[se_mot_v18] = '1'	Did these side effects make you stop using the lesu or use the lesu less frequently?	radio<br>0 No change in use<br>1 Used it less frequently<br>2 Stopped using it
676	diary_lesu1_v18	Section Header: *V. Lesu Diary*<br>How many days did the participant report using LESU #1<br>*Enter number between 0 to 14*	text (integer, Min: 0, Max: 14)
677	diary_lesu2_v18	How many days did the participant report using LESU #2<br>*Enter number between 0 to 14*	text (integer, Min: 0, Max: 14)
678	diary_llin_mom_v18	How many nights did the MOTHER report sleeping under a bed net?<br>*Enter number between 0 to 14*	text (integer, Min: 0, Max: 14)
679	diary_llin_child_v18	How many nights did the CHILD report sleeping under a bed net?<br>*Enter number between 0 to 14*	text (integer, Min: 0, Max: 14)
680	diary_carry_v18	How many days was a lesu used to CARRY the child?<br>*Enter number between 0 to 14*	text (integer, Min: 0, Max: 14)
681	diary_sit_v18	How many days was a lesu used as a place for the child to SIT?<br>*Enter number between 0 to 14*	text (integer, Min: 0, Max: 14)
682	diary_sleep_v18	How many days was a lesu used as a BLANKET when the child was put to sleep?<br>*Enter number between 0 to 14*	text (integer, Min: 0, Max: 14)
683	diary_wash_v18	How many days was LESU #1 WASHED?<br>*Enter number between 0 to 14*	text (integer, Min: 0, Max: 14)
684	diary_wash2_v18	How many days was LESU #2 WASHED?<br>*Enter number between 0 to 14*	text (integer, Min: 0, Max: 14)
685	dc_v18	Section Header: *VI. Discharge Actions*<br>Prior to discharge, ensure the following are complete:	checkbox<br>2 dc_v18___2 Instruct to return to clinic with card if child sick<br>3 dc_v18___3 Remind about next scheduled visit in 2 weeks
686	week_18_clinic_visit_complete	Section Header: *Form Status*<br>Complete?	dropdown<br>0 Incomplete<br>1 Unverified<br>2 Complete
Instrument: **19. Week 20 Clinic Visit** (week_20_clinic_visit)			
687	date_visit_v20	Section Header: *Week 20 Visit*<br>Date of visit<br>*DD-MM-YYYY*	text (date_dmy), Required
688	temp_v20	Section Header: *I. Vital Signs - Child*	text (number, Min: 35, Max: 45), Required

		Axillary temperature<br>*degrees Celsius*	
689	bednet_v20	Section Header: *II. Medical History - Child*<br>Did the child sleep under a bed net last night?	yesno, Required<br>1 Yes<br>0 No
690	fever_v20	Has the child had fever in last two weeks?	yesno, Required<br>1 Yes<br>0 No
691	onset_v20<br>Show the field ONLY if:<br>[fever_v20] = '1'	If yes, when did the fever start<br>*DD-MM-YYYY*	text (date_dmy)
692	sick_v20<br>Show the field ONLY if:<br>[fever_v20] = '0' OR [fever_v20] = '1'	Even if the child has not had a fever, has he or she been otherwise unwell?	yesno, Required<br>1 Yes<br>0 No
693	symp_v20<br>Show the field ONLY if:<br>[sick_v20] = '1'	If yes, what symptoms has the child experienced?	checkbox<br>0 symp_v20___0 Cough<br>1 symp_v20___1 Diarrhea<br>2 symp_v20___2 Ear Ache<br>3 symp_v20___3 Not feeding<br>4 symp_v20___4 Rash<br>5 symp_v20___5 Runny nose<br>6 symp_v20___6 Other (specify below)
694	symp_other_v20<br>Show the field ONLY if:<br>[symp_v20(6)] = '1'	List other symptoms:	notes
695	healthcentre_v20<br>Show the field ONLY if:<br>[fever_v20] = '1' or [sick_v20] = '1'	Has the child been seen at a hospital, health centre, clinic, drug shop, or other medical attendant for these symptoms?	yesno<br>1 Yes<br>0 No
696	hc_where_v20<br>Show the field ONLY if:<br>[healthcentre_v20] = '1'	If yes, where?	radio<br>0 Hospital<br>1 Health Centre<br>2 Drug Shop or Pharmacy<br>3 VHT<br>4 Traditional Medicine
697	medicine_v20<br>Show the field ONLY if:<br>[healthcentre_v20] = '1'	Did the child receive medicine for malaria?	yesno<br>1 Yes<br>0 No
698	med_date_v20<br>Show the field ONLY if:<br>[medicine_v20] = '1'	When did the child take the last dose (i.e. pill) of medicine?<br>*DD-MM-YYYY*	text (date_dmy)
699	mrdt_v20	Section Header: *III. Laboratory Testing - Child*<br>Malaria RDT performed?	yesno, Required<br>1 Yes<br>0 No
700	mrdt_res_v20	Malaria RDT Result	radio

	Show the field ONLY if:<br>[mrdt_v20] = '1'	*Repeat any invalid tests*	0 Negative<br>1 Positive
701	treat_v20<br>Show the field ONLY if:<br>([temp_v20] >= 37.5 or [fever_v20] = '1') and [mrdt_res_v20] = '1'	If mother reported fever or child's temperature was >37.5 C, which antimalarial treatment provided?	radio<br>0 None (explain below)<br>1 Coartem<br>2 Quinine<br>3 Admitted
702	no_treat_v20<br>Show the field ONLY if:<br>[treat_v20] = '0'	Why was treatment NOT given?	notes
703	dbs_v20	Dried blood spots collected?	yesno<br>1 Yes<br>0 No
704	lesu_use_v20	Section Header: *IV. Lesu Questions*<br>Since your last visit, how often did you use the lesu to carry the child?	radio, Required<br>0 Never<br>1 Some days (1 - 3 per week)<br>2 Most days (4 - 6 per week)<br>3 Every day
705	washing_v20	Since your last visit, how many times did you wash the lesu?	text (integer, Min: 0, Max: 50), Required
706	se_v20	Did the child experience any side effects, to include itching or rash, from the lesu?	yesno, Required<br>1 Yes<br>0 No
707	se_symp_v20<br>Show the field ONLY if:<br>[se_v20] = '1'	If yes, what were the child's side effects?	checkbox<br>0 se_symp_v20___0 Headache<br>1 se_symp_v20___1 Itching<br>2 se_symp_v20___2 Nausea or not feeding<br>3 se_symp_v20___3 Rash<br>4 se_symp_v20___4 Other
708	se_other_v20<br>Show the field ONLY if:<br>[se_symp_v20(4)] = '1'	Describe the child's other symptoms:	notes
709	se_impact_v20<br>Show the field ONLY if:<br>[se_v20] = '1'	Did the side effects make you stop using the lesu or use the lesu less frequently?	radio<br>0 No change in use<br>1 Used it less frequently<br>2 Stopped using it
710	se_mot_v20	Did the mother experience any side effects, to include itching or rash, from the lesu?	yesno, Required<br>1 Yes<br>0 No
711	se_symp_mot_v20<br>Show the field ONLY if:<br>[se_mot_v20] = '1'	If yes, what were the side effects?	checkbox<br>0 se_symp_mot_v20___0 Headache<br>1 se_symp_mot_v20___1 Itching<br>2 se_symp_mot_v20___2 Nausea or loss of appetite

			3 se_symp_mot_v20___3 Rash<br>4 se_symp_mot_v20___4 Other
712	se_mot_other_v20<br>Show the field ONLY if:<br>[se_symp_mot_v20(4)] = '1'	Describe other symptoms:	notes
713	se_impact_mot_v20<br>Show the field ONLY if:<br>[se_mot_v20] = '1'	Did these side effects make you stop using the lesu or use the lesu less frequently?	radio<br>0 No change in use<br>1 Used it less frequently<br>2 Stopped using it
714	diary_lesu1_v20	Section Header: *V. Lesu Diary*<br>How many days did the participant report using LESU #1<br>*Enter number between 0 to 14*	text (integer, Min: 0, Max: 14)
715	diary_lesu2_v20	How many days did the participant report using LESU #2<br>*Enter number between 0 to 14*	text (integer, Min: 0, Max: 14)
716	diary_llin_mom_v20	How many nights did the MOTHER report sleeping under a bed net?<br>*Enter number between 0 to 14*	text (integer, Min: 0, Max: 14)
717	diary_llin_child_v20	How many nights did the CHILD report sleeping under a bed net?<br>*Enter number between 0 to 14*	text (integer, Min: 0, Max: 14)
718	diary_carry_v20	How many days was a lesu used to CARRY the child?<br>*Enter number between 0 to 14*	text (integer, Min: 0, Max: 14)
719	diary_sit_v20	How many days was a lesu used as a place for the child to SIT?<br>*Enter number between 0 to 14*	text (integer, Min: 0, Max: 14)
720	diary_sleep_v20	How many days was a lesu used as a BLANKET when the child was put to sleep?<br>*Enter number between 0 to 14*	text (integer, Min: 0, Max: 14)
721	diary_wash_v20	How many days was LESU #1 WASHED?<br>*Enter number between 0 to 14*	text (integer, Min: 0, Max: 14)
722	diary_wash2_v20	How many days was LESU #2 WASHED?<br>*Enter number between 0 to 14*	text (integer, Min: 0, Max: 14)
723	dc_v20	Section Header: *VI. Discharge Actions*<br>Prior to discharge, ensure the following are complete:	checkbox<br>1 dc_v20___1 Retreat lesu according to assignment<br>2 dc_v20___2 Instruct to return to clinic with card if child sick<br>3 dc_v20___3 Remind about next scheduled visit in 2 weeks
724	week_20_clinic_visit_complete	Section Header: *Form Status*<br>Complete?	dropdown<br>0 Incomplete<br>1 Unverified<br>2 Complete
**Instrument: 20. Week 22 Clinic Visit (week_22_clinic_visit)**			
725	date_visit_v22	Section Header: *Week 22 Visit*<br>Date of visit<br>*DD-MM-YYYY*	text (date_dmy), Required
726	temp_v22	Section Header: *I. Vital Signs - Child*<br>Axillary temperature<br>*degrees Celsius*	text (number, Min: 35, Max: 45), Required
727	bednet_v22	Section Header: *II. Medical History - Child*<br>Did the child sleep under a bed net last night?	yesno, Required<br>1 Yes

			0 No
728	fever_v22	Has the child had fever in last two weeks?	yesno, Required<br>1 Yes<br>0 No
729	onset_v22<br>Show the field ONLY if:<br>[fever_v22] = '1'	If yes, when did the fever start<br>*DD-MM-YYYY*	text (date_dmy)
730	sick_v22<br>Show the field ONLY if:<br>[fever_v22] = '0' OR [fever_v22] = '1'	Even if the child has not had a fever, has he or she been otherwise unwell?	yesno, Required<br>1 Yes<br>0 No
731	symp_v22<br>Show the field ONLY if:<br>[sick_v22] = '1'	If yes, what symptoms has the child experienced?	checkbox<br>0 symp_v22___0 Cough<br>1 symp_v22___1 Diarrhea<br>2 symp_v22___2 Ear Ache<br>3 symp_v22___3 Not feeding<br>4 symp_v22___4 Rash<br>5 symp_v22___5 Runny nose<br>6 symp_v22___6 Other (specify below)
732	symp_other_v22<br>Show the field ONLY if:<br>[symp_v22(6)] = '1'	List other symptoms:	notes
733	healthcentre_v22<br>Show the field ONLY if:<br>[fever_v22] = '1' or [sick_v22] = '1'	Has the child been seen at a hospital, health centre, clinic, drug shop, or other medical attendant for these symptoms?	yesno<br>1 Yes<br>0 No
734	hc_where_v22<br>Show the field ONLY if:<br>[healthcentre_v22] = '1'	If yes, where?	radio<br>0 Hospital<br>1 Health Centre<br>2 Drug Shop or Pharmacy<br>3 VHT<br>4 Traditional Medicine
735	medicine_v22<br>Show the field ONLY if:<br>[healthcentre_v22] = '1'	Did the child receive medicine for malaria?	yesno<br>1 Yes<br>0 No
736	med_date_v22<br>Show the field ONLY if:<br>[medicine_v22] = '1'	When did the child take the last dose (i.e. pill) of medicine?<br>*DD-MM-YYYY*	text (date_dmy)
737	mrdt_v22	Section Header: *III. Laboratory Testing - Child*<br>Malaria RDT performed?	yesno, Required<br>1 Yes<br>0 No
738	mrdt_res_v22<br>Show the field ONLY if:<br>[mrdt_v22] = '1'	Malaria RDT Result<br>*Repeat any invalid tests*	radio<br>0 Negative<br>1 Positive
739	treat_v22	If mother reported fever or child's temperature was >37.5 C, which antimalarial treatment provided?	radio

	Show the field ONLY if:<br>([temp_v22] >= 37.5 or [fever_v22] = '1') and [mrdt_res_v22] = '1'		0 None (explain below)<br>1 Coartem<br>2 Quinine<br>3 Admitted
740	no_treat_v22<br>Show the field ONLY if:<br>[treat_v22] = '0'	Why was treatment NOT given?	notes
741	dbs_v22	Dried blood spots collected?	yesno<br>1 Yes<br>0 No
742	lesu_use_v22	Section Header: *IV. Lesu Questions*<br>Since your last visit, how often did you use the lesu to carry the child?	radio, Required<br>0 Never<br>1 Some days (1 - 3 per week)<br>2 Most days (4 - 6 per week)<br>3 Every day
743	washing_v22	Since your last visit, how many times did you wash the lesu?	text (integer, Min: 0, Max: 50), Required
744	se_v22	Did the child experience any side effects, to include itching or rash, from the lesu?	yesno, Required<br>1 Yes<br>0 No
745	se_symp_v22<br>Show the field ONLY if:<br>[se_v22] = '1'	If yes, what were the child's side effects?	checkbox<br>0 se_symp_v22___0 Headache<br>1 se_symp_v22___1 Itching<br>2 se_symp_v22___2 Nausea or not feeding<br>3 se_symp_v22___3 Rash<br>4 se_symp_v22___4 Other
746	se_other_v22<br>Show the field ONLY if:<br>[se_symp_v22(4)] = '1'	Describe the child's other symptoms:	notes
747	se_impact_v22<br>Show the field ONLY if:<br>[se_v22] = '1'	Did the side effects make you stop using the lesu or use the lesu less frequently?	radio<br>0 No change in use<br>1 Used it less frequently<br>2 Stopped using it
748	se_mot_v22	Did the mother experience any side effects, to include itching or rash, from the lesu?	yesno, Required<br>1 Yes<br>0 No
749	se_symp_mot_v22<br>Show the field ONLY if:<br>[se_mot_v22] = '1'	If yes, what were the side effects?	checkbox<br>0 se_symp_mot_v22___0 Headache<br>1 se_symp_mot_v22___1 Itching<br>2 se_symp_mot_v22___2 Nausea or loss of appetite<br>3 se_symp_mot_v22___3 Rash<br>4 se_symp_mot_v22___4 Other
750	se_mot_other_v22<br>Show the field ONLY if:	Describe other symptoms:	notes

	[se_symp_mot_v22(4)] = '1'		
751	se_impact_mot_v22<br>Show the field ONLY if:<br>[se_mot_v22] = '1'	Did these side effects make you stop using the lesu or use the lesu less frequently?	radio<br>0 No change in use<br>1 Used it less frequently<br>2 Stopped using it
752	diary_lesu1_v22	Section Header: *V. Lesu Diary*<br>How many days did the participant report using LESU #1<br>*Enter number between 0 to 14*	text (integer, Min: 0, Max: 14)
753	diary_lesu2_v22	How many days did the participant report using LESU #2<br>*Enter number between 0 to 14*	text (integer, Min: 0, Max: 14)
754	diary_llin_mom_v22	How many nights did the MOTHER report sleeping under a bed net?<br>*Enter number between 0 to 14*	text (integer, Min: 0, Max: 14)
755	diary_llin_child_v22	How many nights did the CHILD report sleeping under a bed net?<br>*Enter number between 0 to 14*	text (integer, Min: 0, Max: 14)
756	diary_carry_v22	How many days was a lesu used to CARRY the child?<br>*Enter number between 0 to 14*	text (integer, Min: 0, Max: 14)
757	diary_sit_v22	How many days was a lesu used as a place for the child to SIT?<br>*Enter number between 0 to 14*	text (integer, Min: 0, Max: 14)
758	diary_sleep_v22	How many days was a lesu used as a BLANKET when the child was put to sleep?<br>*Enter number between 0 to 14*	text (integer, Min: 0, Max: 14)
759	diary_wash_v22	How many days was LESU #1 WASHED?<br>*Enter number between 0 to 14*	text (integer, Min: 0, Max: 14)
760	diary_wash2_v22	How many days was LESU #2 WASHED?<br>*Enter number between 0 to 14*	text (integer, Min: 0, Max: 14)
761	dc_v22	Section Header: *VI. Discharge Actions*<br>Prior to discharge, ensure the following are complete:	checkbox<br>2 dc_v22___2 Instruct to return to clinic with card if child sick<br>3 dc_v22___3 Remind about next scheduled visit in 2 weeks
762	week_22_clinic_visit_complete	Section Header: *Form Status*<br>Complete?	dropdown<br>0 Incomplete<br>1 Unverified<br>2 Complete
**Instrument: 21. Week 24 Clinic Visit (week_24_clinic_visit)**			
763	date_visit_v24	Section Header: *Week 24 (Final) Visit*<br>Date of visit<br>*DD-MM-YYYY*	text (date_dmy), Required
764	height_v24	Section Header: *I. Vital Signs - Child*<br>Height<br>*cm*	text (number, Min: 50, Max: 100), Required
765	weight_v24	Weight<br>*kg*	text (number, Min: 5, Max: 20), Required
766	muac_v24	Mid-Upper Arm Circumference<br>*cm*	text (number, Min: 5, Max: 25), Required
767	temp_v24	Axillary temperature<br>*degrees Celsius*	text (number, Min: 35, Max: 45), Required
768	bednet_v24	Section Header: *II. Medical History - Child*<br>Did the child sleep under a bed net last night?	yesno, Required<br>1 Yes<br>0 No

769	fever_v24	Has the child had fever in last two weeks?	yesno, Required<br>1 Yes<br>0 No
770	onset_v24<br><br>Show the field ONLY if:<br>[fever_v24] = '1'	If yes, when did the fever start<br>*DD-MM-YYYY*	text (date_dmy)
771	sick_v24<br><br>Show the field ONLY if:<br>[fever_v24] = '0' OR [fever_v24] = '1'	Even if the child has not had a fever, has he or she been otherwise unwell?	yesno, Required<br>1 Yes<br>0 No
772	symp_v24<br><br>Show the field ONLY if:<br>[sick_v24] = '1'	If yes, what symptoms has the child experienced?	checkbox<br>0 symp_v24___0 Cough<br>1 symp_v24___1 Diarrhea<br>2 symp_v24___2 Ear Ache<br>3 symp_v24___3 Not feeding<br>4 symp_v24___4 Rash<br>5 symp_v24___5 Runny nose<br>6 symp_v24___6 Other (specify below)
773	symp_other_v24<br><br>Show the field ONLY if:<br>[symp_v24(6)] = '1'	List other symptoms:	notes
774	healthcentre_v24<br><br>Show the field ONLY if:<br>[fever_v24] = '1' or [sick_v24] = '1'	Has the child been seen at a hospital, health centre, clinic, drug shop, or other medical attendant for these symptoms?	yesno<br>1 Yes<br>0 No
775	hc_where_v24<br><br>Show the field ONLY if:<br>[healthcentre_v24] = '1'	If yes, where?	radio<br>0 Hospital<br>1 Health Centre<br>2 Drug Shop or Pharmacy<br>3 VHT<br>4 Traditional Medicine
776	medicine_v24<br><br>Show the field ONLY if:<br>[healthcentre_v24] = '1'	Did the child receive medicine for malaria?	yesno<br>1 Yes<br>0 No
777	med_date_v24<br><br>Show the field ONLY if:<br>[medicine_v24] = '1'	When did the child take the last dose (i.e. pill) of medicine?<br>*DD-MM-YYYY*	text (date_dmy)
778	mrdt_v24	Section Header: *III. Laboratory Testing - Child*<br>Malaria RDT performed?	yesno, Required<br>1 Yes<br>0 No
779	mrdt_res_v24<br><br>Show the field ONLY if:<br>[mrdt_v24] = '1'	Malaria RDT Result<br>*Repeat any invalid tests*	radio<br>0 Negative<br>1 Positive
780	treat_v24<br><br>Show the field ONLY if:	If mother reported fever or child's temperature was >37.5 C, which antimalarial treatment provided?	radio<br>0 None (explain below)

	([temp_v24] >= 37.5 or [fever_v24] = '1') and [mrdt_res_v24] = '1'		1 Coartem<br>2 Quinine<br>3 Admitted
781	no_treat_v24<br><br>Show the field ONLY if:<br>[treat_v24] = '0'	Why was treatment NOT given?	notes
782	cbc_v24	Hemoglobin measured?	yesno<br>1 Yes<br>0 No
783	hb_v24<br><br>Show the field ONLY if:<br>[cbc_v24] = '1'	Hemoglobin (g/dL)	text (number, Min: 3, Max: 20)
784	dbs_v24	Dried blood spots collected?	yesno<br>1 Yes<br>0 No
785	urine_v24	Urine sample collected?	yesno<br>1 Yes<br>0 No
786	bednet_mot_v24	Section Header: *IV. Medical History - Mother*<br>Did the mother sleep under a bed net last night?	yesno, Required<br>1 Yes<br>0 No
787	cosleep_v24<br><br>Show the field ONLY if:<br>[bednet_mot_v24] = '1'	Did the mother sleep under the same net as the child?	yesno<br>1 Yes<br>0 No
788	cbc_mot_v24	Section Header: *V. Laboratory Testing - Mother*<br>Hemoglobin measured?	yesno<br>1 Yes<br>0 No
789	hb_mot_v24<br><br>Show the field ONLY if:<br>[cbc_mot_v24] = '1'	Hemoglobin (g/dL)	text (number, Min: 3, Max: 20)
790	dbs_mot_v24	Dried blood spots collected?	yesno<br>1 Yes<br>0 No
791	urine_mot_v24	Urine sample collected?	yesno<br>1 Yes<br>0 No
792	lesu_use_v24	Section Header: *VI. Lesu Use*<br>Since your last visit, how often did you use the lesu to carry the child?	radio, Required<br>0 Never<br>1 Some days (1 - 3 per week)<br>2 Most days (4 - 6 per week)<br>3 Every day
793	washing_v24	Since your last visit, how many times did you wash the lesu?	text (integer, Min: 0, Max: 50), Required
794	se_v24	Did the child experience any side effects, to include itching or rash, from the lesu?	yesno, Required<br>1 Yes

			0 No
795	se_symp_v24<br>Show the field ONLY if:<br>[se_v24] = '1'	If yes, what were the child's side effects?	checkbox<br>0 se_symp_v24___0 Headache<br>1 se_symp_v24___1 Itching<br>2 se_symp_v24___2 Nausea or not feeding<br>3 se_symp_v24___3 Rash<br>4 se_symp_v24___4 Other
796	se_other_v24<br>Show the field ONLY if:<br>[se_symp_v24(4)] = '1'	Describe the child's other symptoms:	notes
797	se_impact_v24<br>Show the field ONLY if:<br>[se_v24] = '1'	Did the side effects make you stop using the lesu or use the lesu less frequently?	radio<br>0 No change in use<br>1 Used it less frequently<br>2 Stopped using it
798	se_mot_v24	Did the mother experience any side effects, to include itching or rash, from the lesu?	yesno, Required<br>1 Yes<br>0 No
799	se_symp_mot_v24<br>Show the field ONLY if:<br>[se_mot_v24] = '1'	If yes, what were the side effects?	checkbox<br>0 se_symp_mot_v24___0 Headache<br>1 se_symp_mot_v24___1 Itching<br>2 se_symp_mot_v24___2 Nausea or loss of appetite<br>3 se_symp_mot_v24___3 Rash<br>4 se_symp_mot_v24___4 Other
800	se_mot_other_v24<br>Show the field ONLY if:<br>[se_symp_mot_v24(4)] = '1'	Describe other symptoms:	notes
801	se_impact_mot_v24<br>Show the field ONLY if:<br>[se_mot_v24] = '1'	Did these side effects make you stop using the lesu or use the lesu less frequently?	radio<br>0 No change in use<br>1 Used it less frequently<br>2 Stopped using it
802	diary_lesu1_v24	Section Header: *V. Lesu Diary*<br>How many days did the participant report using LESU #1<br>*Enter number between 0 to 14*	text (integer, Min: 0, Max: 14)
803	diary_lesu2_v24	How many days did the participant report using LESU #2<br>*Enter number between 0 to 14*	text (integer, Min: 0, Max: 14)
804	diary_llin_mom_v24	How many nights did the MOTHER report sleeping under a bed net?<br>*Enter number between 0 to 14*	text (integer, Min: 0, Max: 14)
805	diary_llin_child_v24	How many nights did the CHILD report sleeping under a bed net?<br>*Enter number between 0 to 14*	text (integer, Min: 0, Max: 14)
806	diary_carry_v24	How many days was a lesu used to CARRY the child?<br>*Enter number between 0 to 14*	text (integer, Min: 0, Max: 14)
807	diary_sit_v24	How many days was a lesu used as a place for the child to SIT?<br>*Enter number between 0 to 14*	text (integer, Min: 0, Max: 14)
808	diary_sleep_v24	How many days was a lesu used as a BLANKET when the child	text (integer, Min: 0, Max: 14)

		was put to sleep?<br>*Enter number between 0 to 14*	
809	diary_wash_v24	How many days was LESU #1 WASHED?<br>*Enter number between 0 to 14*	text (integer, Min: 0, Max: 14)
810	diary_wash2_v24	How many days was LESU #2 WASHED?<br>*Enter number between 0 to 14*	text (integer, Min: 0, Max: 14)
811	dc_v24	Section Header: *VII. Discharge Actions*<br>Prior to discharge, ensure the following are complete:	checkbox<br>0 dc_v24___0 Obtain punch from lesu<br>1 dc_v24___1 Complete Exit Interview<br>2 dc_v24___2 Give completion bonus (if eligible)<br>3 dc_v24___3 Thank for participation in the study
812	week_24_clinic_visit_complete	Section Header: *Form Status*<br>Complete?	dropdown<br>0 Incomplete<br>1 Unverified<br>2 Complete
**Instrument: 22. Exit Interview (exit_interview)**			
813	exit_date	Date	text (date_dmy), Required
814	exit_use	How did you use the lesu?<br>*Check all that apply*	checkbox, Required<br>0 exit_use___0 Carry child on back<br>1 exit_use___1 Blanket for sleep<br>2 exit_use___2 Cloth to set child down on ground<br>3 exit_use___3 Other<br>Custom alignment: RH
815	exit_use_other	If other, please describe	notes<br>Custom alignment: RH
816	exit_lessmore	Dod you use this lesu more or less than lesus with your other children?	dropdown, Required<br>0 Less<br>1 More<br>2 Not applicable - this was first child<br>Custom alignment: RH
817	exit_lessmore_des<br>Show the field ONLY if:<br>[exit_lessmore] = '0' or [exit_lessmore] = '1'	Why did you use it less or more?	notes<br>Custom alignment: RH
818	exit_se	Did you observe your child having any side effects from the lesu over the study period?	yesno, Required<br>1 Yes<br>0 No
819	exit_se_des<br>Show the field ONLY if:<br>[exit_se] = '1'	If yes, please describe:	notes
820	exit_mat_se	Did you have any side effects from wearing the lesu over the study period?	yesno, Required<br>1 Yes<br>0 No
821	exit_mat_se_des<br>Show the field ONLY if:	If yes, please describe:	notes

	[exit_mat_se] = '1'		
822	exit_ben	Did you observe any benefit against mosquitos and other bugs from the lesu?	yesno, Required<br>1 Yes<br>0 No
823	exit_ben_des<br>Show the field ONLY if:<br>[exit_ben] = '1'	If yes, please describe:	notes
824	exit_rec	Would you recommend lesus treated with insecticide to a family member or friend?	yesno<br>1 Yes<br>0 No
825	exit_rec_des	Please explain why or why not?	notes
826	exit_pay	Would you be willing to pay more for a lesu that was treated with insecticide?	yesno, Required<br>1 Yes<br>0 No
827	exit_pay_amt<br>Show the field ONLY if:<br>[exit_pay] = '1'	How much more would you be willing to pay for a treated lesu than a standard lesu?	dropdown<br>0 1,000 shillings<br>1 2,500 shillings<br>2 5,000 shillings<br>3 10,000 shillings<br>4 >10,000 shillings<br>Custom alignment: RH
828	exit_final	Do you have any final comments about the lesu?	notes, Required<br>Custom alignment: RH
829	exit_interview_complete	Section Header: *Form Status*<br>Complete?	dropdown<br>0 Incomplete<br>1 Unverified<br>2 Complete
**Instrument: 23. Participant Discontinuation (participant_discontinuation)**			
830	d_date	Date of Discontinuation:	text (date_dmy), Required
831	d_reason	Please describe the reason for discontinuation:	notes, Required
832	participant_discontinuation_complete	Section Header: *Form Status*<br>Complete?	dropdown<br>0 Incomplete<br>1 Unverified<br>2 Complete
**Instrument: 24. Adverse Events (adverse_events)**			
833	adv_date	Date of adverse event (AE) or serious adverse event (SAE):	text (date_dmy), Required
834	adv_describe	Please describe the event (including which safety parameter(s) exceeded the threshold):	notes, Required
835	adv_type	Section Header: *THE FOLLOWING TO BE COMPLETED BY PI:*<br>Is this an AE or SAE?	radio<br>1 AE<br>2 SAE
836	adv_severity	What is the severity?	radio<br>1 Mild

			2 Moderate<br>3 Severe
837	adv_related	Is the event related or unrelated to study participation?	radio<br>1 Related (possible, probable, definite)<br>2 Not related (unlikely, not related)
838	adverse_events_complete	Section Header: *Form Status*<br>Complete?	dropdown<br>0 Incomplete<br>1 Unverified<br>2 Complete
**Instrument: A1. Acute Visit 1 - Case Report Form (a1_acute_visit_form)**			
839	acute_date	Date of visit<br>*DD-MM-YYYY*	text (date_dmy, Min: 2022-06-01, Max: 2025-05-30), Required, Identifier
840	acute_site	Location of visit	radio, Required<br>0 Bugoye Health Centre<br>1 Mukathi Health Centre
841	acute_hpi	Section Header: *I. Clinical History*<br>What is the reason for today's visit?	radio, Required<br>0 Fever<br>1 Cough or difficulty breathing<br>2 Vomiting<br>3 Diarrhea<br>4 Other (specify below)
842	acute_hpi_other<br>Show the field ONLY if:<br>[acute_hpi] = '4'	If another reason, please list	text
843	acute_symp	In addition to the above, what other symptoms has the child experienced, if any?	checkbox<br>0 acute_symp___0 Cough<br>1 acute_symp___1 Headache<br>2 acute_symp___2 Ear Ache<br>3 acute_symp___3 Not feeding<br>4 acute_symp___4 Vomiting<br>5 acute_symp___5 Diarrhea<br>6 acute_symp___6 Rash<br>7 acute_symp___7 Runny nose<br>8 acute_symp___8 Sore throat<br>9 acute_symp___9 Other (specify below)
844	acute_onset	When did the first symptoms start?<br>*DD-MM-YYYY*	text (date_dmy, Min: 2022-06-01, Max: 2025-05-30)
845	acute_net	Did the child sleep under a bed net last night?	yesno<br>1 Yes<br>0 No
846	acute_care	Has the child been seen at a hospital, health centre, clinic, drug shop, or other medical attendant for these symptoms?	yesno<br>1 Yes<br>0 No
847	acute_care_site	If yes, where?	radio

	Show the field ONLY if:<br>[acute_care] = '1'		0 Hospital<br>1 Health Centre<br>2 Drug Shop or Pharmacy<br>3 VHT<br>4 Traditional Medicine
848	acute_malmed<br>Show the field ONLY if:<br>[acute_care] = '1'	Did the child receive medicine for malaria?	yesno<br>1 Yes<br>0 No
849	acute_med_dose<br>Show the field ONLY if:<br>[acute_malmed] = '1'	When did the child finish the last dose (i.e. pill) of medicine?<br>*DD-MM-YYYY*	text (date_dmy, Min: 2022-06-01, Max: 2025-05-30)
850	acute_temp	Section Header: *II. Vital Signs - Child*<br>Axillary temperature<br>*degrees Celsius*	text (number, Min: 35, Max: 45), Required
851	acute_mrdt	Section Header: *III. Laboratory Testing - Child*<br>Was a malaria RDT performed?	yesno, Required<br>1 Yes<br>0 No
852	acute_mrdt_res<br>Show the field ONLY if:<br>[acute_mrdt] = '1'	Malaria RDT Result<br>*Repeat any invalid tests*	radio<br>0 Negative<br>1 Positive
853	acute_treat<br>Show the field ONLY if:<br>[acute_mrdt_res] = '1'	Which antimalarial treatment provided?	radio, Required<br>0 None (explain below)<br>1 Coartem<br>2 Quinine (oral)<br>3 Admitted for IV artesunate
854	acute_no_treat<br>Show the field ONLY if:<br>[acute_treat] = '0'	Why was treatment NOT given?	notes
855	acute_dbs	Dried blood spots collected?Ensure labeled with Study ID, date, and clearly marked as ACUTE VISIT	yesno<br>1 Yes<br>0 No
856	acute_dispo	Section Header: *III. Disposition and Discharge*<br>What was the child's disposition at the end of the visit?	radio, Required<br>0 Discharged home<br>1 Admitted to IPD<br>2 Transferred to other facility (e.g., St. Pauls, Kilembe)<br>3 Other (explain below)
857	acute_ipd_dc<br>Show the field ONLY if:<br>[acute_dispo] = '1'	If child was admitted to IPD, what was date of discharge?	text (date_dmy, Min: 2022-06-01, Max: 2025-05-30), Identifier
858	acute_trans<br>Show the field ONLY if:<br>[acute_dispo] = '2'	If child was transferred, please describe reason for transfer and where patient was transferred.	notes
859	acute_dispo_other<br>Show the field ONLY if:	If other, please describe here:	notes

	[acute_dispo] = '3'		
860	acute_other	Record any other information about the visit here, including treatments given if non-malarial illness suspected	notes
861	acute_check	Prior to discharge, ensure the following are complete:	checkbox<br>0 acute_check___0 Ensure DBS made and labeled as acute visit<br>1 acute_check___1 Remind about next scheduled visit
862	a1_acute_visit_form_complete	Section Header: *Form Status*<br>Complete?	dropdown<br>0 Incomplete<br>1 Unverified<br>2 Complete
**Instrument: A2. Acute Visit 2 - Case Report Form (a2_acute_visit_2_case_report_form)**			
863	acute_date_v2	Date of visit<br>*DD-MM-YYYY*	text (date_dmy, Min: 2022-06-01, Max: 2025-05-30), Required, Identifier
864	acute_site_v2	Location of visit	radio, Required<br>0 Bugoye Health Centre<br>1 Mukathi Health Centre
865	acute_hpi_v2	Section Header: *I. Clinical History*<br>What is the reason for today's visit?	radio, Required<br>0 Fever<br>1 Cough or difficulty breathing<br>2 Vomiting<br>3 Diarrhea<br>4 Other (specify below)
866	acute_hpi_other_v2<br>Show the field ONLY if:<br>[acute_hpi_v2] = '4'	If another reason, please list	text
867	acute_symp_v2	In addition to the above, what other symptoms has the child experienced, if any?	checkbox<br>0 acute_symp_v2___0 Cough<br>1 acute_symp_v2___1 Headache<br>2 acute_symp_v2___2 Ear Ache<br>3 acute_symp_v2___3 Not feeding<br>4 acute_symp_v2___4 Vomiting<br>5 acute_symp_v2___5 Diarrhea<br>6 acute_symp_v2___6 Rash<br>7 acute_symp_v2___7 Runny nose<br>8 acute_symp_v2___8 Sore throat<br>9 acute_symp_v2___9 Other (specify below)
868	acute_onset_v2	When did the first symptoms start?<br>*DD-MM-YYYY*	text (date_dmy, Min: 2022-06-01, Max: 2025-05-30)
869	acute_net_v2	Did the child sleep under a bed net last night?	yesno<br>1 Yes<br>0 No
870	acute_care_v2	Has the child been seen at a hospital, health centre, clinic, drug shop, or other medical attendant for these symptoms?	yesno<br>1 Yes

			0 No
871	acute_care_site_v2<br>Show the field ONLY if:<br>[acute_care_v2] = '1'	If yes, where?	radio<br>0 Hospital<br>1 Health Centre<br>2 Drug Shop or Pharmacy<br>3 VHT<br>4 Traditional Medicine
872	acute_malmed_v2<br>Show the field ONLY if:<br>[acute_care_v2] = '1'	Did the child receive medicine for malaria?	yesno<br>1 Yes<br>0 No
873	acute_med_dose_v2<br>Show the field ONLY if:<br>[acute_malmed_v2] = '1'	When did the child finish the last dose (i.e. pill) of medicine?<br>*DD-MM-YYYY*	text (date_dmy, Min: 2022-06-01, Max: 2025-05-30)
874	acute_temp_v2	Section Header: *II. Vital Signs - Child*<br>Axillary temperature<br>*degrees Celsius*	text (number, Min: 35, Max: 45), Required
875	acute_mrdt_v2	Section Header: *III. Laboratory Testing - Child*<br>Was a malaria RDT performed?	yesno, Required<br>1 Yes<br>0 No
876	acute_mrdt_res_v2<br>Show the field ONLY if:<br>[acute_mrdt_v2] = '1'	Malaria RDT Result<br>*Repeat any invalid tests*	radio<br>0 Negative<br>1 Positive
877	acute_treat_v2<br>Show the field ONLY if:<br>[acute_mrdt_res_v2] = '1'	Which antimalarial treatment provided?	radio, Required<br>0 None (explain below)<br>1 Coartem<br>2 Quinine (oral)<br>3 Admitted for IV artesunate
878	acute_no_treat_v2<br>Show the field ONLY if:<br>[acute_treat_v2] = '0'	Why was treatment NOT given?	notes
879	acute_dbs_v2	Dried blood spots collected?Ensure labeled with Study ID, date, and clearly marked as ACUTE VISIT	yesno<br>1 Yes<br>0 No
880	acute_dispo_v2	Section Header: *III. Disposition and Discharge*<br>What was the child's disposition at the end of the visit?	radio, Required<br>0 Discharged home<br>1 Admitted to IPD<br>2 Transferred to other facility (e.g., St. Pauls, Kilembe)<br>3 Other (explain below)
881	acute_ipd_dc_v2<br>Show the field ONLY if:<br>[acute_dispo_v2] = '1'	If child was admitted to IPD, what was date of discharge?	text (date_dmy, Min: 2022-06-01, Max: 2025-05-30), Identifier
882	acute_trans_v2<br>Show the field ONLY if:<br>[acute_dispo_v2] = '2'	If child was transferred, please describe reason for transfer and where patient was transferred.	notes

883	acute_dispo_other_v2<br>Show the field ONLY if:<br>[acute_dispo_v2] = '3'	If other, please describe here:	notes
884	acute_other_v2	Record any other information about the visit here, including treatments given if non-malarial illness suspected	notes
885	acute_check_v2	Prior to discharge, ensure the following are complete:	checkbox<br>0 acute_check_v2___0 Ensure DBS made and labeled as acute visit<br>1 acute_check_v2___1 Remind about next scheduled visit
886	a2_acute_visit_2_case_report_form_complete	Section Header: *Form Status*<br>Complete?	dropdown<br>0 Incomplete<br>1 Unverified<br>2 Complete
**Instrument: A3. Acute Visit 3 - Case Report Form (a3_acute_visit_3_case_report_form)**			
887	acute_date_v3	Date of visit<br>*DD-MM-YYYY*	text (date_dmy, Min: 2022-06-01, Max: 2025-05-30), Required, Identifier
888	acute_site_v3	Location of visit	radio, Required<br>0 Bugoye Health Centre<br>1 Mukathi Health Centre
889	acute_hpi_v3	Section Header: *I. Clinical History*<br>What is the reason for today's visit?	radio, Required<br>0 Fever<br>1 Cough or difficulty breathing<br>2 Vomiting<br>3 Diarrhea<br>4 Other (specify below)
890	acute_hpi_other_v3<br>Show the field ONLY if:<br>[acute_hpi_v3] = '4'	If another reason, please list	text
891	acute_symp_v3	In addition to the above, what other symptoms has the child experienced, if any?	checkbox<br>0 acute_symp_v3___0 Cough<br>1 acute_symp_v3___1 Headache<br>2 acute_symp_v3___2 Ear Ache<br>3 acute_symp_v3___3 Not feeding<br>4 acute_symp_v3___4 Vomiting<br>5 acute_symp_v3___5 Diarrhea<br>6 acute_symp_v3___6 Rash<br>7 acute_symp_v3___7 Runny nose<br>8 acute_symp_v3___8 Sore throat<br>9 acute_symp_v3___9 Other (specify below)
892	acute_onset_v3	When did the first symptoms start?<br>*DD-MM-YYYY*	text (date_dmy, Min: 2022-06-01, Max: 2025-05-30)
893	acute_net_v3	Did the child sleep under a bed net last night?	yesno<br>1 Yes<br>0 No

894	acute_care_v3	Has the child been seen at a hospital, health centre, clinic, drug shop, or other medical attendant for these symptoms?	yesno<br>1 Yes<br>0 No
895	acute_care_site_v3<br>Show the field ONLY if:<br>[acute_care_v3] = '1'	If yes, where?	radio<br>0 Hospital<br>1 Health Centre<br>2 Drug Shop or Pharmacy<br>3 VHT<br>4 Traditional Medicine
896	acute_malmed_v3<br>Show the field ONLY if:<br>[acute_care_v3] = '1'	Did the child receive medicine for malaria?	yesno<br>1 Yes<br>0 No
897	acute_med_dose_v3<br>Show the field ONLY if:<br>[acute_malmed_v3] = '1'	When did the child finish the last dose (i.e. pill) of medicine?<br>*DD-MM-YYYY*	text (date_dmy, Min: 2022-06-01, Max: 2025-05-30)
898	acute_temp_v3	Section Header: *II. Vital Signs - Child*<br>Axillary temperature<br>*degrees Celsius*	text (number, Min: 35, Max: 45), Required
899	acute_mrdt_v3	Section Header: *III. Laboratory Testing - Child*<br>Was a malaria RDT performed?	yesno, Required<br>1 Yes<br>0 No
900	acute_mrdt_res_v3<br>Show the field ONLY if:<br>[acute_mrdt_v3] = '1'	Malaria RDT Result<br>*Repeat any invalid tests*	radio<br>0 Negative<br>1 Positive
901	acute_treat_v3<br>Show the field ONLY if:<br>[acute_mrdt_res_v3] = '1'	Which antimalarial treatment provided?	radio, Required<br>0 None (explain below)<br>1 Coartem<br>2 Quinine (oral)<br>3 Admitted for IV artesunate
902	acute_no_treat_v3<br>Show the field ONLY if:<br>[acute_treat_v3] = '0'	Why was treatment NOT given?	notes
903	acute_dbs_v3	Dried blood spots collected?Ensure labeled with Study ID, date, and clearly marked as ACUTE VISIT	yesno<br>1 Yes<br>0 No
904	acute_dispo_v3	Section Header: *III. Disposition and Discharge*<br>What was the child's disposition at the end of the visit?	radio, Required<br>0 Discharged home<br>1 Admitted to IPD<br>2 Transferred to other facility (e.g., St. Pauls, Kilembe)<br>3 Other (explain below)
905	acute_ipd_dc_v3<br>Show the field ONLY if:<br>[acute_dispo_v3] = '1'	If child was admitted to IPD, what was date of discharge?	text (date_dmy, Min: 2022-06-01, Max: 2025-05-30), Identifier
906	acute_trans_v3	If child was transferred, please describe reason for transfer	notes

	Show the field ONLY if: [acute_dispo_v3] = '2'	and where patient was transferred.	
907	acute_dispo_other_v3<br>Show the field ONLY if: [acute_dispo_v3] = '3'	If other, please describe here:	notes
908	acute_other_v3	Record any other information about the visit here, including treatments given if non-malarial illness suspected	notes
909	acute_check_v3	Prior to discharge, ensure the following are complete:	checkbox<br>0 | acute_check_v3___0 | Ensure DBS made and labeled as acute visit<br>1 | acute_check_v3___1 | Remind about next scheduled visit
910	a3_acute_visit_3_case_report_form_complete	Section Header: *Form Status*<br>Complete?	dropdown<br>0 | Incomplete<br>1 | Unverified<br>2 | Complete
