# Supplementary material for: Permethrin-treated baby wraps for the prevention of malaria in children: Protocol for a double-blind, randomized placebo-controlled controlled trial in western Uganda
Source: PLoS One. 2023 Apr 27;18(4):e0284322. doi: 10.1371/journal.pone.0284322 (PMC10138219; doi:10.1371/journal.pone.0284322)
Supplement: S2 File — (DOCX) [file pone.0284322.s003.docx]

**MUST-UNC RESEARCH COLLABORATION**

| **PROTOCOL TITLE:** | Getting Malaria “off the backs” of women and children in western Uganda | |
| --- | --- | --- |
|  |  |  |
| **PROTOCOL VERSION:** | Version 1.1 |  |
|  |  |  |
| **PROTOCOL DATE:** | 6/7/22 |  |
|  |  |  |
| **INSTITUTIONS:** | University of North Carolina at Chapel Hill (UNC) | |
|  | Mbarara University of Science and Technology (MUST)  Peoples’ Health and Economic Development (PHEALED) | |
|  |  |  |
| **FUNDING:** | Doris Duke Charitable Foundation  Clinical Scientist Development Award  Grant # 2021196 | |
|  |  |  |
| **PERIOD:** | 7/1/2021 – 6/30/2024 |  |
|  |  |  |
| **PRINCIPAL INVESTIGATOR(S)** | Ross M Boyce MD, MSc  Assistant Professor  Division of Infectious Diseases  CB 7036, 111 Mason Farm Rd  Chapel Hill, NC 27599  (+001) 919-966-2537  roboyce@med.unc.edu | |
|  | Edgar Mulogo, PhD, MPH  Associate Professor  Dept. of Community Health  PO Box 1410  Mbarara, Uganda  (+256) 772-433-508  emulogo@must.ac.ug | |

STATEMENT OF COMPLIANCE

The study will be conducted in accordance with the International Conference on Harmonization guidelines for Good Clinical Practice (ICH E6), the Code of Federal Regulations on the Protection of Human Subjects (45 CFR Part 46), and the Terms of Award. All personnel involved in the conduct of this study have completed human subjects protection training.

SIGNATURE PAGE

The signature below constitutes the approval of this protocol and the attachments, and provides the necessary assurances that this trial will be conducted according to all stipulations of the protocol, including all statements regarding confidentiality, and according to local legal and regulatory requirements and applicable U.S. federal regulations and ICH guidelines.

Principal Investigator or Clinical Site Investigator:

| Signed: |  | Date: |  |
| --- | --- | --- | --- |
|  | Name: Ross M. Boyce, MD, MSc | | |
|  | Title: Assistant Professor of Medicine | | |

TABLE OF CONTENTS

PAGE

[STATEMENT OF COMPLIANCE 2](#_Toc85200724)

[SIGNATURE PAGE 3](#_Toc85200725)

[TABLE OF CONTENTS 4](#_Toc85200726)

[LIST OF ABBREVIATIONS 7](#_Toc85200727)

[PROTOCOL SYNOPSIS 9](#_Toc85200728)

[1. KEY ROLES AND CONTACT INFORMATION 11](#_Toc85200729)

[1 INTRODUCTION: BACKGROUND INFORMATION AND SCIENTIFIC RATIONALE 13](#_Toc85200730)

[1.1 Background Information 13](#_Toc85200731)

[1.2 Pilot Study 14](#_Toc85200732)

[1.3 Rationale 15](#_Toc85200733)

[1.3.1 Intervention 15](#_Toc85200734)

[1.3.2 Study Site & Population 16](#_Toc85200735)

[1.4 Potential Risks and Benefits 16](#_Toc85200736)

[1.4.1 Potential Risks 16](#_Toc85200737)

[1.4.2 Potential Benefits 17](#_Toc85200738)

[2 OBJECTIVES 18](#_Toc85200739)

[2.1 Study Objectives 18](#_Toc85200740)

[2.2 Study Outcome Measures 18](#_Toc85200741)

[3 STUDY DESIGN 20](#_Toc85200742)

[4 STUDY ENROLLMENT AND WITHDRAWAL 21](#_Toc85200743)

[4.1 Subject Inclusion Criteria 21](#_Toc85200744)

[4.2 Subject Exclusion Criteria 21](#_Toc85200745)

[4.3 Strategies for Recruitment and Retention 21](#_Toc85200746)

[4.3.1 Sensitization Meetings 21](#_Toc85200747)

[4.3.2 Information Sessions 22](#_Toc85200748)

[4.3.3 Participant Incentives 22](#_Toc85200749)

[4.3.4 Contacts 22](#_Toc85200750)

[4.4 Treatment Assignment Procedures 23](#_Toc85200751)

[4.4.1 Randomization Procedures 23](#_Toc85200752)

[4.4.2 Masking Procedures 23](#_Toc85200753)

[4.5 Subject Withdrawal 23](#_Toc85200754)

[4.5.1 Reasons for Withdrawal 23](#_Toc85200755)

[4.5.2 Handling of Subject Withdrawals or Subject Discontinuation of Study Intervention 24](#_Toc85200756)

[4.6 Premature Termination or Suspension of Study 24](#_Toc85200757)

[5 STUDY INTERVENTION 25](#_Toc85200758)

[5.1 Study Product Description 25](#_Toc85200759)

[5.1.1 Acquisition 25](#_Toc85200760)

[5.1.2 Formulation, Packaging, and Labeling 25](#_Toc85200761)

[5.1.3 Product Storage and Stability 25](#_Toc85200762)

[5.2 Dosage, Preparation, and Administration of Study Product 26](#_Toc85200763)

[5.3 Modification of Study Product Administration for a Subject 26](#_Toc85200764)

[5.4 Accountability Procedures for the Study Product 26](#_Toc85200765)

[5.5 Assessment of Subject Compliance with Study Product Administration 26](#_Toc85200766)

[5.6 Concomitant Medications/Treatments 27](#_Toc85200767)

[5.7 Administration of Intervention 27](#_Toc85200768)

[5.8 Procedures for Training Interventionists and Monitoring Intervention Fidelity 27](#_Toc85200769)

[5.9 Assessment of Subject Compliance with Study Intervention 27](#_Toc85200770)

[6 STUDY SCHEDULE 28](#_Toc85200771)

[6.1 Screening 28](#_Toc85200772)

[6.1.1 Information Sessions 28](#_Toc85200773)

[6.1.2 Final Screening (Day -21 to -7) 28](#_Toc85200774)

[6.2 Household Survey 28](#_Toc85200775)

[6.3 Baseline Clinic Visit (Day 0) 29](#_Toc85200776)

[6.4 Bi-Monthly Visits (Days 14, 28, 42, 56, 70, 84, 98, 112, 126, 140, 154) 29](#_Toc85200777)

[6.5 Final Study Visit (Day 168) 30](#_Toc85200778)

[6.6 Unscheduled Visits 31](#_Toc85200779)

[7 STUDY PROCEDURES /EVALUATIONS 32](#_Toc85200780)

[7.1 Study Procedures/Evaluations 32](#_Toc85200781)

[7.1.1 Demographic and Medical History 32](#_Toc85200782)

[7.1.2 Household 32](#_Toc85200783)

[7.1.3 Physical examinations 32](#_Toc85200784)

[7.1.4 Biological specimens 32](#_Toc85200785)

[7.1.5 Self-reported data 33](#_Toc85200786)

[7.1.6 Observation logs 33](#_Toc85200787)

[7.1.7 Entomological surveillance 33](#_Toc85200788)

[7.2 Laboratory Procedures/Evaluations 33](#_Toc85200789)

[7.2.1 Screening Laboratory Evaluations 33](#_Toc85200790)

[7.2.2 Clinical Laboratory Evaluations 33](#_Toc85200791)

[7.2.3 Special Assays or Procedures 34](#_Toc85200792)

[7.2.4 Specimen Preparation, Handling, and Storage 34](#_Toc85200793)

[7.2.5 Specimen Shipment 34](#_Toc85200794)

[8 ASSESSMENT OF SAFETY 35](#_Toc85200795)

[8.1 Specification of Safety Parameters 35](#_Toc85200796)

[8.1.1 Unanticipated Problems 35](#_Toc85200797)

[8.1.2 Adverse Events 35](#_Toc85200798)

[8.1.3 Serious Adverse Events 35](#_Toc85200799)

[8.2 Time Period and Frequency for Event Assessment and Follow-Up 36](#_Toc85200800)

[8.3 Characteristics of an Adverse Event 36](#_Toc85200801)

[8.3.1 Relationship to Study Intervention 36](#_Toc85200802)

[8.3.2 Expectedness of SAEs 36](#_Toc85200803)

[8.3.3 Severity of Event 37](#_Toc85200804)

[8.4 Reporting Procedures 37](#_Toc85200805)

[8.4.1 Unanticipated Problem Reporting to IRB 37](#_Toc85200806)

[8.4.2 Reporting of Pregnancy 37](#_Toc85200807)

[8.5 Halting Rules 38](#_Toc85200808)

[9 STUDY OVERSIGHT 39](#_Toc85200809)

[10 STATISTICAL CONSIDERATIONS 40](#_Toc85200810)

[10.1 Study Hypotheses 40](#_Toc85200811)

[10.2 Sample Size Considerations 40](#_Toc85200812)

[10.3 Final Analysis Plan 40](#_Toc85200813)

[10.3.1 Analysis of primary outcome (AIM 1) 41](#_Toc85200814)

[10.3.2 Analysis of secondary outcomes (AIM 2) 41](#_Toc85200815)

[11 SOURCE DOCUMENTS AND ACCESS TO SOURCE DATA/DOCUMENTS 43](#_Toc85200816)

[12 QUALITY CONTROL AND QUALITY ASSURANCE 44](#_Toc85200817)

[13 ETHICS/PROTECTION OF HUMAN SUBJECTS 45](#_Toc85200818)

[13.1 Ethical Standard 45](#_Toc85200819)

[13.2 Institutional Review Board 45](#_Toc85200820)

[13.3 Informed Consent Process 45](#_Toc85200821)

[13.4 Exclusion of Women, Minorities, and Children (Special Populations) 45](#_Toc85200822)

[13.5 Subject Confidentiality 46](#_Toc85200823)

[13.6 Future Use of Stored Specimens and Other Identifiable Data 46](#_Toc85200824)

[14 DATA HANDLING AND RECORD KEEPING 47](#_Toc85200825)

[14.1 Data Management Responsibilities 47](#_Toc85200826)

[14.2 Data Capture Methods 47](#_Toc85200827)

[14.3 Study Records Retention 47](#_Toc85200828)

[14.4 Protocol Deviations 47](#_Toc85200829)

[15 PUBLICATION/DATA SHARING POLICY 48](#_Toc85200830)

[16 LITERATURE REFERENCES 49](#_Toc85200831)

[SUPPLEMENTAL MATERIALS 53](#_Toc85200832)

[APPENDICES 54](#_Toc85200833)

[APPENDIX A: SCHEDULE OF EVENTS 55](#_Toc85200834)

[Appendix A1 – Schedule of Events for Participating Mothers 55](#_Toc85200835)

[Appendix A2 – Schedule of Events for Participating Children and Lesus 56](#_Toc85200836)

LIST OF ABBREVIATIONS

| ACT | Artemisinin Combination Therapy |
| --- | --- |
| ADL | Activities of Daily Living |
| AL | Artemether/Lumefantrine |
| AE | Adverse Event/Adverse Experience |
| BHC | Bugoye Level III Health Center |
| CDC | Centers for Disease Control and Protection |
| CFR | Code of Federal Regulations |
| CHW | Community Health Worker |
| CO | Clinical Officer |
| CONSORT | Consolidated Standards of Reporting Trials |
| CRF | Case Report Form |
| DBS | Dried Blood Spot |
| DSMB | Data and Safety Monitoring Board |
| EPA | Environmental Protection Agency |
| FDA | Food and Drug Administration |
| FWA | Federalwide Assurance |
| GCP | Good Clinical Practice |
| Hb | Hemoglobin |
| HIPAA | Health Insurance Portability and Accountability Act |
| ICF | Informed Consent Form |
| ICH | International Conference on Harmonisation |
| ICMJE | International Committee of Medical Journal Editors |
| IRB | Institutional Review Board |
| IRS | Indoor Residual Spraying |
| ITT | Intention-to-treat |
| ISM | Independent Safety Monitor |
| LLIN | Long-lasting Insecticidal Net |
| MOH | Ministry of Health |
| MUST | Mbarara University of Science and Technology |
| N | Number (typically refers to subjects) |
| NIH | National Institutes of Health |
| OPD | Outpatient Department |
| PCR | Polymerase Chain Reaction |
| PHI | Protected Health Information |
| PI | Principal Investigator |
| PPE | Personal Protective Equipment |
| QA | Quality Assurance |
| QC | Quality Control |
| RDT | Rapid Diagnostic Test |
| SAE | Serious Adverse Event/Serious Adverse Experience |
| SOP | Standard Operating Procedure |
| UNC | University of North Carolina at Chapel Hill |
| UNCST | Uganda National Council on Science and Technology |
| US | United States |
| WHO | World Health Organization |

PROTOCOL SYNOPSIS

| **Title:** | *Getting Malaria “off the backs” of women and children in western Uganda* |
| --- | --- |
| **Précis:** | The study is a double-blind, randomized placbo-controlled trial of permethrin-treated *lesus* to prevent *P. falciparum* malaria in children 6–24 months of age conducted at two sites in rural western Uganda. Participating mother-infant pairs will receive a new LLIN and two permethrin-treated or untreated *lesus* at enrollment. We will follow participants longitudinally for 6 months. Participants will present to one of the two study clinics when a fever develops, where they will be evaluated, tested, and treated, if positive, for malaria. Participants will also attend scheduled clinic visits every 2 weeks for routine surveillance of adverse effects and to test for asymptomatic infection. Re-treatment and sham re-treatment of *lesus* will occur each month. |
| **Objectives:** | The scientific objective is to demonstrate the protective effect of permethrin-treated *lesus* against *P. falciparum* malaria in infants and young children. |
|  | **Primary Outcome**: Incidence of clinical, RDT-confirmed *P. falciparum* malaria in children |
|  | **Secondary Outcome(s)**: Change in child’s hemoglobin and growth parameters |
| **Population:** | The general population of mothers and children (6–18 months of age at enrollment) residing in a malaria-endemic, rural area of western Uganda. The total sample size will be 400 mother-infant pairs with 200 pairs in each arm. |
| **Phase:** | N/A |
| **Number of Sites:** | 1. Bugoye Level III Health Center  2. Mukathi Level III Health Center |
| **Description of Intervention:** | Permethrin-treated cloth used to carry infants and young children on a mother’s back (aka “lesu”) |
| **Study Duration:** | 36 Months |
| **Subject Participation:** | 6 Months |
| **Time to Complete Enrollment:** | 12 Months |

**PROTOCOL SCHEMA**


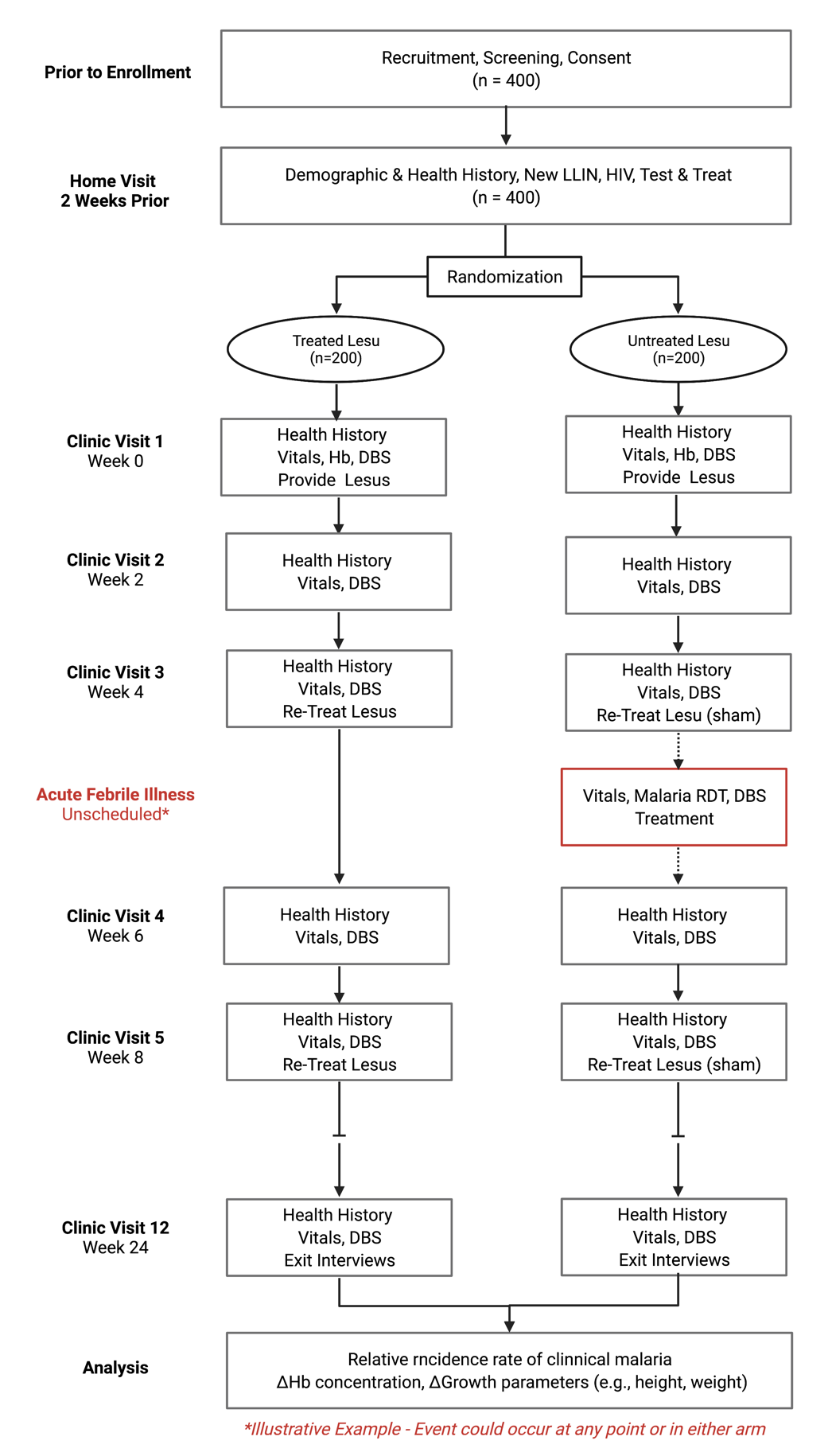


# 1. KEY ROLES AND CONTACT INFORMATION

| **Principal Investigator:** | Ross M. Boyce, MD, MSc  Assistant Professor, Division of Infectious Diseases  University of North Carolina at Chapel Hill  111 Mason Farm Road, CB 7036  Chapel Hill, NC 27599  [roboyce@med.unc.edu](mailto:roboyce@med.unc.edu) |
| --- | --- |
| **DDCF Program Official:** | Sindy Escobar Alvarez, PhD  Program Director for Medical Research  Doris Duke Charitable Foundation  650 5th Ave, 19th Floor  New York, NY 10019 |
| **Clinical Site Investigators:** | Edgar M. Mulogo, PhD, MSc, MPH  Associate Professor, Department of Community Health  Mbarara University of Science and Technology  PO Box 1410, Mbarara, Uganda  (+256) 772-433-508  [emulogo@must.ac.ug](mailto:emulogo@must.ac.ug) |
| **Institutions:** | Institute of Global Health and Infectious Diseases  University of North Carolina at Chapel Hill  130 Mason Farm Road, CB 7030  Chapel Hill, NC 27599  Contact: Diana Stanley  Administrative Director for Research and Compliance  (919) 843-7935  [diana_stanley@med.unc.edu](mailto:diana_stanley@med.unc.edu)  Department of Community Health  Mbarara University of Science and Technology  PO Box 1410, Mbarara, Uganda  (+256) 772-433-508  Contact: Margaret Mbabzi  MUST Grants Office  (+256) 782-558-202  [mmmbabazi@must.ac.ug](mailto:mmmbabazi@must.ac.ug)  Peoples Health and Economic Development (PHEALED)  PO Box 250, Kasese, Uganda  Contact: Moses Ntaro  Executive Director  (+256) 772-669-346  [phealed2017@gmail.com](mailto:phealed2017@gmail.com) |
| **Other Key Personnel:** | Raquel Reyes, MD, MPA  Associate Professor, Division of Hospital Medicine  University of North Carolina at Chapel Hill  Role: Co-Investigator (Clinical)  [raquel.reyes@med.unc.edu](mailto:raquel.reyes@med.unc.edu)  Bonnie Shook-Sa, DrPH  Assistant Professor, Department of Biostatistics  Gillings School of Global Public Health  University of North Carolina at Chapel Hill  Role: Co-Investigator (Lead Biostatistician)  [bshooksa@live.unc.edu](mailto:bshooksa@live.unc.edu)  Jonathan J. Juliano, MD, MSPH  Professor, Division of Infectious Diseases  University of North Carolina at Chapel Hill  Role: Co-Investigator (Laboratory)  [jonathan_juliano@med.unc.edu](mailto:jonathan_juliano@med.unc.edu)  Dana Giandomenico, MPH  VEER Program Manager  University of North Carolina at Chapel Hill  Role: Program Manager (US)  [danag@med.unc.edu](mailto:danag@med.unc.edu)  Emmanuel Baguma, BSc  MUST-UNC Bugoye Site Director  Mbarara University of Science and Technology  Role: Site Director (Uganda)  [bagumaemmanuel2016@gmail.com](mailto:bagumaemmanuel2016@gmail.com)  Dan Nyehangane, MSc  Laboratory Coordinator  Epicentre Mbarara Research Base  Role: Co-Investigator (Laboratory)  [dan.nyehangane@epicentre.msf.org](mailto:dan.nyehangane@epicentre.msf.org)  Stephanie Richardson, PhD  Professor, Environmental Health Sciences  East Carolina University  Role: Co-Investigator (Laboratory)  [richardss@ecu.edu](mailto:richardss@ecu.edu) |

# INTRODUCTION: BACKGROUND INFORMATION AND SCIENTIFIC RATIONALE

## Background Information

Over the past two decades, the burden of *Plasmodium falciparum* malaria has substantially declined, with mortality in SSA decreasing by more than 35% [1]. However, current interventions, including long-lasting insecticidal nets (LLIN) and indoor residual spraying (IRS), are insufficient to drive vectorial capacity below the critical thresholds needed to interrupt transmission [2, 3]. In addition, these household-based interventions can drive a dynamic set of ecological and evolutionary responses to selection pressure [4]. For example, LLINs and IRS will favor *Anopheles* mosquito species and feeding behaviors that avoid contact with the interventions, either by feeding on peri-domestic animals, outdoors, or in the early evening when residents are outside the home and household-based measures offer little protection [5-8].

As evidence of these challenges, the last three *World Malaria Reports* published by the World Health Organization (WHO) suggest that progress against malaria has stalled and may even be slipping backwards in high-burden countries, particularly those in SSA [9]. Uganda has one of the highest burdens of malaria, representing 5% of cases and 3% of deaths globally [9, 10]. Despite progress, malaria still accounts for approximately 20% of outpatient visits and inpatient admissions [11]. Uganda has observed widespread rise in the prevalence of mosquitoes that are resistant to the first-line insecticides used in LLIN and IRS programs [12, 13]. Furthermore, there is emerging evidence that local malaria vectors, primarily *Anopheles gambiae* and *A.* *funestus* are increasingly exhibiting feeding behaviors that may not bring them into contact with existing interventions [14]. Thus, further innovations in malaria control are urgently needed [15-18].


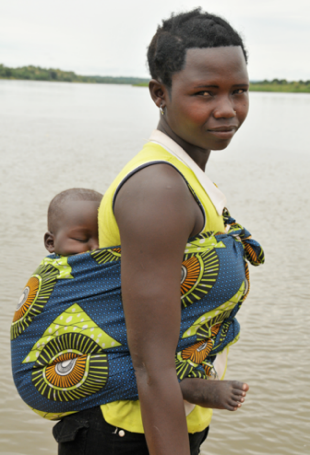
In pursuit of this goal, we sought to leverage the traditional practice of mothers carrying young children on their backs utilizing wraps made from locally purchased cloth (**Figure 1**). The wrap, called a *lesu* in Uganda, also serves as a blanket or swaddle for children when they are put down or put to bed. Thus, mother and child spend much of the day in contact with the cloth. We hypothesized that when treated with an insecticide or repellent, the *lesu* might provide an additional layer of protection against malaria. A similar intervention has been shown effective for preventing mosquito and tick bites among outdoor workers in the Southeastern United States [19, 20]. Our approach has many potential advantages including: (i) targeting the most vulnerable (i.e. young children), (ii) integrating with existing cultural norms, and (iii) complementing current prevention strategies by offering protection against outdoor- and/or day-time biting *Anopheles* mosquitoes.

**Figure 1:** Ugandan mother carrying her child in a *lesu*.

## Pilot Study

As the first step towards testing this hypothesis, we conducted a pilot randomized controlled trial (RCT) of permethrin-treated *lesus* (NCT04102592) to assess safety (i.e., skin reactions, nausea), acceptability, and feasibility before proposing a larger clinical trial. Leveraging the existing network of village-based community health workers, we enrolled 50 mother-infant pairs from four villages neighboring the Bugoye Level III Health Center, our primary clinical site in rural western Uganda. Study staff conducted baseline home visits to document demographic and household characteristics, including malaria knowledge and care-seeking behaviors using a modified questionnaire from the most recent *Uganda Demographic and Health Survey* [21]. Upon completion of the survey, all participants received a new LLIN with guidance that the net was intended for the child.


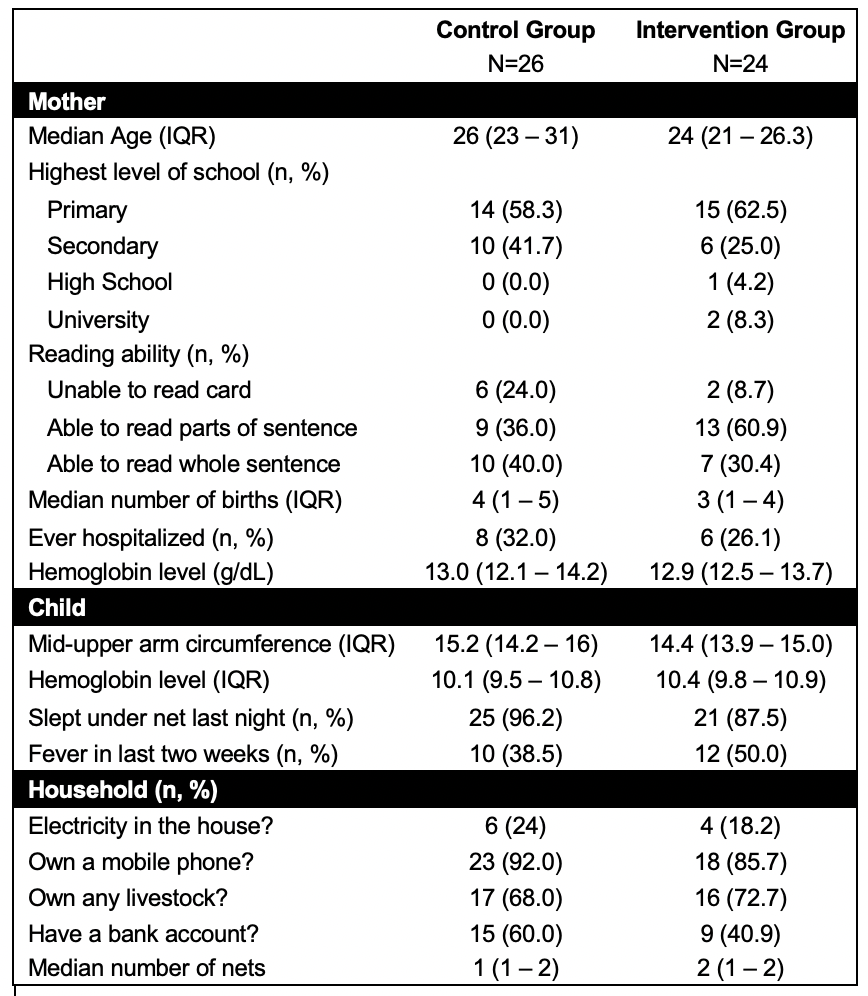


Participating mother-infant pairs were randomized to either permethrin-treated (intervention) or untreated (control) *lesus*. The *lesus* of participants in the intervention group were treated and subsequently retreated each month with 0.5% permethrin (Sawyer Products, Safety Harbor, FL). The *lesus* of participants in the control group underwent sham treatment and re-treatment in which the cloth was soaked in water for a period of time similar to that of the intervention group. Participants and clinical staff but not administrative staff were blinded to group assignments.

**Table 1:** Summary of demographic and clinical variables among pilot study participants stratified by intervention group.

Every two weeks, participants attended study visits where clinical staff assessed mothers and infants for adverse reactions and performed testing for malaria using a malaria RDT. Research staff administered a brief questionnaire of recent medical history (e.g., rash, fever), care-seeking, and frequency of *lesu* use and washing. We also measured the child’s mid-upper arm circumference (MUAC), a validated tool for assessing nutritional status [22] and Hb levels at the initial and 12-week visits. At study completion, participating mothers completed a semi-structured interview eliciting perceptions of safety and effectiveness. Study procedures were approved by the University of North Carolina Institutional Review Board (18-1819), the Mbarara University of Science and Technology Research Ethics Committee (05/08-18), and the Uganda National Council of Science and Technology (SS 4833).

A total of 48 of 50 mother-infant pairs completed study activities. Two participants only completed baseline activities and replacements were subsequently recruited bringing the total to 50 participants. Overall, caregivers had relatively low educational attainment, multiple children in the home, and limited resources, although these characteristics were generally well-balanced between the groups. Almost all mothers reported using the *lesu* to carry the child on their back, while about one-third (n=17, 34%) also reported using the *lesu* as a blanket to put the child down to sleep.

At the conclusion of the 12-week follow-up period, only one adverse event - a transient, mild rash - was reported in a child in the intervention group. This rash was not confirmed at the time of physical examination. No side effects were reported by mothers. Mothers in the intervention group did report washing *lesus* more frequently compared to those in the control group (4.7 vs 3.5 times per 2-week period). Rates of care-seeking (28 visits vs. 27 visits), malaria RDT positivity at any visit (8.4% vs. 10.7%), malaria treatment (6 events vs 5 events), and changes in Hb (0.78 g/dL vs. 0.87 g/dL) were not significantly different between the intervention and control group, respectively, although the study was not powered to detect differences. We did observe a greater change in MUAC between baseline and final visit (0.57 cm vs. -0.14 cm) among children in the intervention group. There was no difference in reported level of satisfaction with the *lesu* between intervention and control groups as evidenced by questions assessing willingness to recommend and willingness to pay extra for a treated *lesu*.

## Rationale

### Intervention

Permethrin is a synthetic insecticide with a well-established safety record, being utilized to treat LLINs, military uniforms, and other clothing [23-25]. The CDC recommends the treatment of clothing with permethrin to prevent mosquito bites in all populations, and specifically reiterated this guidance for pregnant women traveling to Zika-endemic areas to reduce the risk of infection [26]. Notably, permethrin also has a modest repellent effect, which may prevent biting even when mosquitoes are resistant to the killing effect [27].

The *lesus* of participants in the intervention group will be treated and subsequently retreated each month with 0.5% permethrin (Sawyer Products, Safety Harbor, FL) – the same concentration approved by the U.S. Environmental Protection Agency (EPA) and used in military and civilian applications. While the frequency of retreatment was greater than that used with early studies of insecticide-treated nets, our concern is that washing, especially if *lesus* were frequently soiled and washed using traditional methods, might cause premature washout of the permethrin. This has been reported in previous studies of permethrin-treated school uniforms when used to prevent dengue infection [28]. Instead, our intent is to (i) test for potential adverse effects associated with persistent contact at optimal concentrations and (ii) minimize any differences in the preliminary efficacy aims due to washout.

###
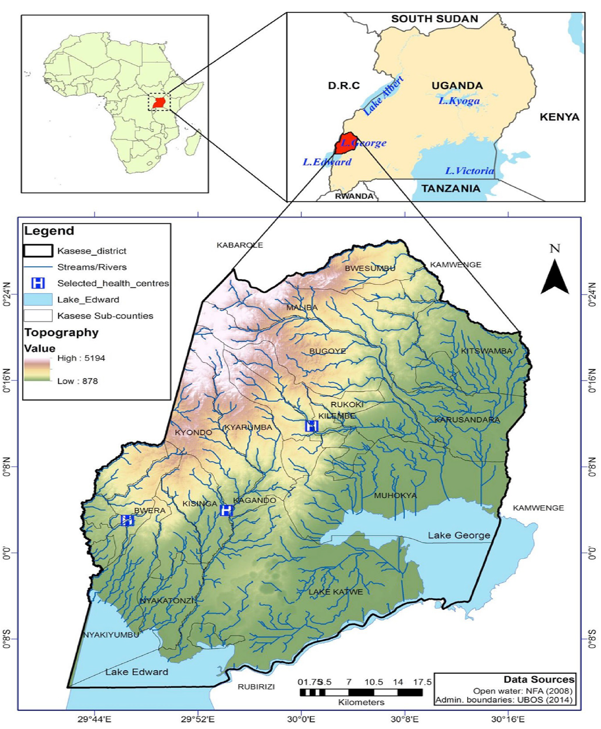
Study Site & Population

The study will take place in the Bugoye and Malaiba sub-counties located in the Kasese District of western Uganda (**Figure 2**). The two sub-counties have populations of 50,249 and 47,769 respectively, with approximately one-fifth of the population being children less than five years of age [29]. Subsistence farming (e.g. cassava, sweet potatoes, maize) represents the primary economic activity, although a high proportion of households also keep livestock, including chickens (88%), goats (74%), and cattle (14%) in the peri-domestic space [30]. The vast majority of residents do not have access to water or electricity in the house, and one-third of households live ≥5 km to the nearest public health facility [29]. This population is generally representative of rural populations throughout malaria-endemic East Africa.

**Figure 2:** Maps showing the location and topography of the proposed study areas. From Mirembe et al, *Plos One* 2017.

The climate in Bugoye permits year-round malaria transmission marked by semi-annual transmission peaks typically following the end of the rainy seasons in May and December [31]. The most recent malaria indicator surveys undertaken in the mid-western region (2014-15) and Tooro sub-national region (2018-19) which include the study area, reported *P. falciparum* parasitemia rates (PfPR) of 17.4% and 7.3%, respectively [32, 33]. However, in a recent cross-sectional survey of more than 2,100 households in the Bugoye Sub-County, we found that the PfPR among children 2 to 8 years of age was upwards of 30% in many of the low-elevation villages. We will recruit and enroll participants from these high transmission villages located along the river basins. The duration of study participation for each mother-child pair is 6 months, which is intended to capture one dry and one wet season.

## Potential Risks and Benefits

### Potential Risks

- **Skin Irritation** – Participants in the intervention group, especially young children, may develop dermatitis or allergic reactions resulting from direct contact with the permethrin. Only one such episode was reported in the pilot study, which was mild and resolved prior to clinical evaluation.
- **Phlebotomy** – Participants may experience discomfort when blood is drawn. There is also a very small risk of bruising and/or infection associated with the blood draws.
- **Loss of Privacy** – There is also the risk of loss of privacy and/or confidentiality concerning medical history and test results should any study documents be compromised or lost.
- **Change in Behavior** – Participating mothers may alter usual activities intended to prevent mosquito bites (e.g., LLIN use, repellents, long-sleeved clothing) assuming that they are protected by the *lesu*.

### Potential Benefits

- **New LLINs, *Lesus*, and Personal Protective Equipment (PPE)** – Participants will receive new LLINs to ensure that mother and child have access to coverage in the home, along with two *lesus* for carrying the child, and PPE (e.g., facemasks, sanitizer) to reduce the spread of COVID-19 during study activities. Participants will keep the LLINs and *lesus* upon completion of the study.
- **Reduced risk of malaria** – Participants in the intervention group may receive an additional level of protection against malaria and other vector-borne diseases as a result of utilizing the treated-*lesu*.
- **Prioritized Case Management** – Participants in both groups will have expedited, no-cost access to the study clinics for evaluation and treatment. The study will ensure there are no stockouts of diagnostics (e.g., RDTs) or anti-malarial treatments.
- **Knowledge Generation** – Participants will be contributing to the evaluation of a novel intervention that, if effective, may help reduce the incidence of malaria not only in the local community, but across SSA.

# OBJECTIVES

## Study Objectives

The overarching goal of this proposed project is to expand the malaria “toolbox” with a scalable, low-tech, low-cost intervention that leverages existing cultural practices and established insecticides to reduce the burden of *P. falciparum* malaria among young children in SSA. Our scientific objective is to demonstrate the protective effect of permethrin-treated *lesus* against *P. falciparum* malaria in infants and young children. To test this hypothesis, we propose to:

**AIM 1: Determine the effectiveness of permethrin-treated *lesus* to prevent *P. falciparum* malaria infection among infants and young children (6–18 months of age).** We will conduct a randomized controlled trial of 400 women-infant pairs at two sites in rural western Uganda leveraging methods similar to those employed in our pilot study. In brief, all participants will be followed longitudinally to capture incident febrile illness, even when occurring between scheduled visits. Our primary outcome is clinical malaria, defined as the presence of typical symptoms (e.g., fever, lethargy) and a positive malaria RDT during observation. Specifically, we hypothesize that the intervention will reduce malaria cases among infants and young children by at least 30% relative to children receiving untreated *lesus*.

**AIM 2: Explore the effectiveness of permethrin-treated versus untreated *lesus* to prevent *P. falciparum* malaria infection among mothers.** Using the same methods outlined in Aim 1, we will assess potential reductions in malaria incidence among mothers as an additional benefit of *lesu* use. If the permethrin-treated *lesu* is effective, it could provide preliminary evidence to support the use of insecticide-treated clothing as a complementary strategy to prevent malaria in pregnancy. We hypothesize that the intervention will have a modest impact, defined by at least a 10% relative reduction, in malaria parasitemia, either symptomatic or asymptomatic, among mothers.

## Study Outcome Measures

| **Study Measure** | **Method of Collection** |
| --- | --- |
| 1. Primary Outcome |  |
| 1.A. Incidence of clinical malaria in child | (1) Acute febrile illness AND positive RDT at scheduled or unscheduled study clinic visit  - or -  (2) Written documentation of fever AND positive RDT from non-study clinic visit |
| 2. Secondary Outcomes |  |
| 2.A. Change in child’s Hb level | Hb measured at baseline (Week 0), Week 12, and Week 24 visits using HemoCue® device. |
| 2.B. Change in child’s growth measures | Height, weight, measured at baseline (Week 0), Week 12, and Week 24 visits. |
| 2.C. Change in child’s nutritional status | MUAC measured at baseline (Week 0), Week 12, and Week 24 visits [22]. |
| 2.D. Asymptomatic parasitemia in children | Presence of malaria parasites on bi-weekly dried DBS as determined by qPCR. |
| 2.E. Hospitalization for malaria | Self-reported history of hospitalization on bi-weekly questionnaire AND confirmation by study staff. |
| 2.F. Clinical malaria in mother | Same as 1.A. |
| 2.G. Change in mother’s Hb level | Same as 2.A. |
| 2.H. Burden of care-seeking | Self-reported history of clinic visits, work days missed, health expenditures on bi-weekly questionnaires. |
| 3. Safety Measures |  |
| 3.A. Adverse reaction to treated-*lesu* | Self-reported history on bi-weekly questionnaire and confirmation by clinical staff. |
| 3.B. Severe adverse event (SAE) | Care-seeking, hospitalization, or death attributable to the intervention. |
| 4. Covariates of Interest |  |
| 4.A. Socioeconomic status | Questions from baseline survey on ownership of mobile phone, vehicle, etc. [34] |
| 4.B. Maternal education | Self-reported history on baseline questionnaire. |
| 4.C. Frequency of *lesu* use and washing | Self-reported history on bi-weekly questionnaire, use diaries, direct observation. |
| 4.D. Residual permethrin levels | Punches of *lesu* cloth taken at 2-, 4-, 6-month visits tested by gas chromatography [35] |
| 4.E. *Anopheles* mosquito density | CDC light trap collections in peri-domestic space (indoor/outdoor) with species identification |

# STUDY DESIGN

The proposed study is a double-blind, randomized controlled trial of permethrin-treated *lesus* to prevent *P. falciparum* malaria in children 6–18 months of age at study enrollment conducted at two sites in rural western Uganda. Participants will be randomized to one of two arms: permethrin-treated (intervention) or untreated (control) *lesus.* Participating mother-infant pairs will receive a new LLIN and two permethrin-treated or untreated *lesus* at enrollment. The total sample size will be 400 mother-infant pairs with 200 pairs in each arm. We will follow participants longitudinally for six months. Participants will be instructed and incentivized to present to one of the two study clinics when a fever develops, where they will be evaluated, tested, and treated, if positive, for malaria. Participants will also attend scheduled clinic visits every two weeks for routine surveillance of adverse effects and to test for asymptomatic infection. Re-treatment and sham re-treatment of *lesus* will occur each month.

# STUDY ENROLLMENT AND WITHDRAWAL

## Subject Inclusion Criteria

In order to be eligible to participate in this study, an individual must meet all of the following criteria:

- Provide signed and dated informed consent form (ICF)
- Willing to comply with all study procedures and be available for the duration of the study
- Female of any age with a child 6 to 18 months of age at the time of enrollment
- Resident of village located in Bugoye, Maliba, or Mubuku sub-counties. Recruitment will initially focus on residents of villages within 2 km of study clinics in order to facilitate attendance, but we will not restrict enrollment to residents of these villages.

## Subject Exclusion Criteria

An individual who meets any of the following criteria will be excluded from participation in this study:

- Mother or child taking any malaria chemoprevention regimen, including individuals living with HIV or exposed children who are taking cotrimoxazole
- Child with known sickle cell disease
- Known allergic reactions to components of the study product(s)
- Treatment with another investigational drug or other intervention
- Anything that would place the individual at increased risk or preclude the individual’s full compliance with or completion of the study.

## Strategies for Recruitment and Retention

### Sensitization Meetings

Prior to study implementation, we will conduct a series of sensitization meetings with leaders including clinical staff at local health facilities, village chairpersons, and community health workers (CHWs) to disseminate information about the study aims, methods, and anticipated risks/benefits of participation. There will be ample opportunity for attendees to ask questions and provide feedback. We will ask CHWs to distribute information about the study to eligible women in their respective coverage areas, including the date and time of informational meetings for interested women.

### Information Sessions

At the informational meeting conducted in the village, study staff fluent in the local language (e.g., Lukhonzo), will describe the study aims, methods and anticipated risks/benefits of participation in a group setting. Women who are interested in participating will move to a private area where they will have an opportunity to ask additional questions. Study staff will confirm eligibility criteria and record the individual’s address and preferred contact information to schedule a household visit. On the agreed upon date, a study staff member will travel to the household and ask the individual to provide written consent for participation, which will be immediately followed by the initial household survey.

### Participant Incentives

Participants will be eligible to receive the following incentives for participation:

| Event | Eligibility | Amount (UGX* / USD) |
| --- | --- | --- |
| Baseline Home Visit | Visit Completed | 10,000 / $3.00 |
| Bi-Weekly Study Visit (12) | Attends Visit (Transport Supplement) | 8,000 / $2.25 |
| Bi-Weekly Study Visit (12) | Communication Supplement | 2,000 / $0.60 Airtime |
| Bi-Weekly Study Visit (12) | Completed *Lesu* Use/Wash Diary | In-Kind (e.g., Soap, Sugar) |
| Final Visit / Exit Interview | Attended ≥80% of visits | 20,000 / $6.00 |
| **POTENTIAL TOTAL** | | 180,000 / $50.00 |

**Approximate UGX to USD conversion; may change based on exchange rate*

### Contacts

On enrollment, participants will be asked to provide the best contact information, including a mobile phone number, if available. Study staff will confirm if this phone is owned/used by the participant or belongs to someone else (e.g., neighbor, relative). In addition, we will ask for permission to send reminders for upcoming study visits via text or phone call. If a participant confirms attendance, then no further contact will be made. Participants will receive cellular airtime credit for this purpose (see Section 4.3.3). Study staff will make up to three text or phone contacts prior to a scheduled study visit.

If study staff are unable to confirm attendance after three attempts, staff will contact the CHW responsible for the area and request assistance contacting the participant, which may include up to three visits to the home. Similar procedures will be used to contact participants after a missed visit.

## Treatment Assignment Procedures

### Randomization Procedures

Participants will be randomized in a 1:1 ratio between intervention and control arms with randomization stratified by age group (age 6–11 months and 12–18 months) and by study site (Bugoye and Malaiba sub-counties). We will utilize stratified randomization to ensure an even distribution between arms at each clinical site and within each age group. Blocks will be variable in size to ensure allocation concealment. The randomization protocol will be developed and implemented by a University of North Carolina at Chapel Hill (UNC) biostatistician not involved in the direct enrollment of participants. Statistical software will be used to generate the list of random assignments with a 1:1 ratio. The randomization sequences will then be uploaded to the Research Electronic Data Capture (REDCap) database. Participants will be randomized upon enrollment using the REDCap Randomization Module on portable tablet devices equipped with cellular internet capability. Participants and health center staff but not research staff will be blinded to the allocation. Sham treatments and re-treatments of *lesus* will be employed to maintain blinding.

### Masking Procedures

Study participants and clinical providers will remain blinded to allocation of the intervention until database lock. Initial treatment and monthly re-treatment of lesus will be performed with either 0.5% permethrin or water (i.e., sham treatment) in order to maintain blinding. Study staff involved in re-treatment (permethrin or sham) of lesus will be unblinded.

## Subject Withdrawal

Participants will be followed until participant closeout, withdrawal of consent, or death. A participant may withdraw from the study at any time at his/her own request or may be withdrawn at any time at the discretion of the investigator for safety, behavioral, compliance, or administrative reasons. This is expected to be uncommon. If the participant withdraws consent for disclosure of future information, the sponsor may retain and continue to use any data collected before such a withdrawal of consent.

### Reasons for Withdrawal

Subjects are free to withdraw from participation in the study at any time upon request. An investigator may terminate a study subject’s participation in the study if:

- Any clinical AE, laboratory abnormality, or other medical condition or situation occurs such that continued participation in the study would not be in the best interest of the subject.
- The subject meets an exclusion criterion (either newly developed or not previously recognized) that precludes further study participation. This may include change of residence outside the study area.
- The subject is “lost to follow-up” defined as failure to attend three consecutive study visits and staff are unable to contact by phone or proxy.

### Handling of Subject Withdrawals or Subject Discontinuation of Study Intervention

Given the established safety profile of the intervention there is minimal risk of continued *lesu* use outside the parameters of the study. Therefore, subjects who withdraw or otherwise discontinue participation will not be followed further.

## Premature Termination or Suspension of Study

This study may be suspended or prematurely terminated if there is sufficient reasonable cause. Written notification, documenting the reason for study suspension or termination, will be provided by the suspending or terminating party to the sponsor and relevant regulatory authorities. If the study is prematurely terminated or suspended, the principal investigator (PI) will promptly inform the institutional review board (IRB) and will provide the reason(s) for the termination or suspension.

Circumstances that may warrant termination include, but are not limited to:

- Determination of unexpected, significant, or unacceptable risk to subjects.
- Insufficient adherence to protocol requirements.
- Data that are not sufficiently complete and/or evaluable.

# STUDY INTERVENTION

## Study Product Description

Permethrin is a synthetic derivative of the naturally occurring pyrethrins found in chrysanthemum flowers. Permethrin acts on nerve cell membranes of the mosquito to disrupt the sodium channel current that regulates the polarization. This results in delayed repolarization, paralysis, and ultimately death of the mosquito. Permethrin also has a modest repellent effect, which may prevent biting even when mosquitoes are resistant to the killing effect [27]. Permethrin has been widely used in LLINs to prevent malaria in endemic areas and is the only repellant currently registered to treat fabric in the United States [36].

Permethrin treated clothing has a well-established safety record. Studies conducted among military personnel wearing permethrin-treated clothing demonstrated that exposure from chronic daily wear correlates with duration of exposure and is higher than the background exposure among the general population, but calculated daily exposures were still lower than exposure from topical pharmaceutical application [24, 25].

In data taken from studies of the topical permethrin formulation used to treat pregnant women with scabies or lice, no increase in the risk of congenital abnormalities was noted among children exposed *in utero* [37, 38]. The FDA classifies permethrin cream as Class B during pregnancy and the WHO considers permethrin compatible with breastfeeding [39].

### Acquisition

All permethrin will be obtained directly from the manufacturer (Sawyer Products, Safety Harbor, FL). *Lesus,* of all the same material and design, will be obtained from local vendors.

### Formulation, Packaging, and Labeling

Permethrin will be acquired as 40% concentrate from the manufacturer available in 250 mL bottles packed in cases of 12 bottles. Labeling of individual bottles will be “INSECT/ARTHROPOD REPELLENT PROTECTIVE TREATMENT FOR MILITARY BATTLE DRESS UNIFORM*.”* A safety data sheet (MSDS 002778A) will accompany each case of bottles [40].

### Product Storage and Stability

This material is not regulated by the U.S. Department of Transportation as a hazardous material. General storage instructions are to not use or store near heat, sparks, open flame, or any other ignition sources. Staff will wear chemical-resistant gloves such as Barrier® laminate, neoprene rubber, nitrile rubber, or Viton® when handling, along with a long-sleeved shirt, long pants, socks, and shoes. The product will be stored in a cool, dry place, under lock and key. All containers will be kept closed. The storage area will be posted as a pesticide storage area with instruction to keep away from heat, sparks, open flame, and any other ignition sources as well as strong acids, bases, and oxidizers. All product will be utilized prior to the labeled expiration date.

## Dosage, Preparation, and Administration of Study Product

Prior to application, permethrin concentrate will be diluted to 0.5% concentration. This will be achieved by adding 12.5 mL of 40% concentrate and adding water until a total volume of 1 L is reached (i.e., mixing ratio is 1.25 w/w concentrate and 98.75 w/w water).

*Lesus* of participants randomized to the intervention group will be soaked in 0.5% permethrin in accordance with the manufacturer’s instructions. In brief, *lesus* will be rolled tightly and secured with rubber bands or ties. The rolled *lesus* will be placed in 1 gal plastic bags and approximately 100 mL of permethrin will be added. The bag will be gently massaged until the cloth is completely soaked. After 1 hour, the *lesu* will be removed, unrolled, and hung to dry in an area that is not exposed to direct sunlight.

Re-treatments occur every 4 weeks of study participation. *Lesus* of participants assigned to the control group will follow similar procedures, but re-treatment will take place with water only (i.e., sham treatment).

## Modification of Study Product Administration for a Subject

No dose adjustments or modifications to the frequency of re-treatment are planned.

## Accountability Procedures for the Study Product

Two *lesus* will be provided to each participant at the first clinic visit. Participants will be asked to bring study *lesus* to each visit for visual inspection.

## Assessment of Subject Compliance with Study Product Administration

Staff will instruct participants that the provided *lesus* should be used exclusively during the study period. Participants will be asked to maintain a written log of daily *lesu* use and washing. These logs will be returned to study staff at the bi-monthly study visits.

In addition, staff will take small punches of *lesus* at the 2-, 4-, and 6-month visits. Harvested fabric swatches will be placed in individual mylar bags and stored in an opaque container at 20^o^ C until transport to the Richards lab at East Carolina University. Swatches will be transferred to individual 60 mL amber glass vials containing 40 mL acetone and soaked for 6 hours to elute permethrin. A portion of the extract (1 pL) will be analyzed directly by capillary GC with flame ionization detector using an Agilent GC 6850 in accordance with previously published protocols [35, 41].

## Concomitant Medications/Treatments

Participants will be asked to document frequency of LLIN use each night on the daily log as noted in Section 5.5. Staff will also ask about any treatments of the home environment such as IRS. Staff will also ask participants about use of any medications with antimalarial effects including, but not limited to:

- Antimalarials:
  - Artemisinin combination therapies (e.g., artemether/lumefantrine)
  - Quinine
  - Sulfadoxine-pyrimethamine
  - Chloroquine
- Antibiotics with antimalarial properties
  - Doxycycline
  - Cotrimoxazole

## Administration of Intervention

Treatment of *lesus*, either with permethrin or sham, will take place during in-person visits. No treatments or re-treatments will take place outside this setting. Study staff responsible for treatment and re-treatment, who will be unblinded to the intervention, will confirm the group assignment against randomization table prior to treatment and re-treatment. During treatment or sham treatment, each ziploc bag will be labeled with the study ID to prevent confusion.

## Procedures for Training Interventionists and Monitoring Intervention Fidelity

Having worked on the pilot study, many study team members are experienced with many of the methods and procedures. Prior to implementation, however, we will conduct a week-long training event, which will review procedures for treatment of *lesus*. In addition to didactic instruction, we will conduct a practical exercise. All staff will be required to demonstrate proficiency with the process.

## Assessment of Subject Compliance with Study Intervention

In addition to daily use logs, we will ask participants for permission to perform intermittent, direct observation of daily use. Study staff will visit participants households and observe use throughout the day with particular emphasis on early morning and early evening hours when individuals are not yet under the cover of a LLIN, but *Anopheles* mosquitoes are likely active in the peri-domestic space. Participants will be notified when selected for observation and scheduled for a day that is convenient.

# STUDY SCHEDULE

Each individual’s participation in the study, defined from initial recruitment to the final study visit, is anticipated to take place over a period of approximately 8 months. A complete Schedule of Events is shown in **Appendix A**. Descriptions of each participant encounter are provided below.

## Screening

### Information Sessions

Initial recruitment, which will be conducted by local CHWs, will target all women with children under 18 months of age residing in villages located within 2 km of a study clinic. Potentially eligible women will be invited to attend an information meeting, where staff will describe the study aims, methods and anticipated risks/benefits of participation in a group setting. Women who are interested in participating will move to a private area where they will have an opportunity to ask additional questions. Study staff will the screen women based on responses to eligibility questions. We will not review medical records or perform confirmatory diagnostic testing (e.g., HIV) at this visit. If the patient meets criteria, staff will record the individual’s full name, household location, contact information, and schedule a household visit within the next 21 days.

### Final Screening (Day -21 to -7)

On the scheduled date, a study staff member will travel to the household, review the consent form, answer any questions, and ask the individual to provide written consent for participation. The consent form will include language stating that a negative rapid HIV test is required for before enrollment and that if the result is positive, the individual will not be eligible to participate (i.e., screen failure), but will be immediately linked to care. If negative, this will be immediately followed by the household survey.

## Household Survey

After enrollment is complete, study staff will:

- Record the household location using a handheld GPS device or smart phone.
- Document demographic and household characteristics via a questionnaire modified from the most recent Demographic and Health Survey [21].
- Perform a malaria RDT on both mother and child and if positive, receive weight-based treatment with AL, which is first-line therapy for uncomplicated malaria in Uganda [42].
- Issue a new LLIN (Permanet® 2.0, Vestergaard S.A., Switzerland) with guidance that it should be utilized to protect the child.
- At the conclusion of the survey, study staff will make an appointment date for the first clinic visit and provide the participant with an identification card, including the unique study ID number and telephone contacts.

## Baseline Clinic Visit (Day 0)

Participants will be scheduled for a baseline visit at either the Bugoye or Mukathi Level III Health Center. During the baseline visit, study staff will:

- Confirm participant identify and study number.
- Administer a questionnaire reviewing changes in medical history since the household visit.
- Measure and record vital signs, including:
  - Child’s height, weight and MUAC
  - Child’s axillary temperature
- Have 3–4 mL of blood drawn by venous phlebotomy from the child to:
  - Perform testing for Hb concentration
  - Create DBS for qPCR testing at a later date.
  - Store sera for future seroprevalence studies of vector-borne disease
- Have 6–8 mL of blood drawn by venous phlebotomy from the mother to:
  - Perform testing for Hb concentration
  - Create DBS for qPCR testing at a later date.
  - Store sera for future seroprevalence studies of vector-borne disease
- Issue the permethrin-treated or untreated *lesus* (2) according to randomization
- Provide an in-kind incentive (e.g., sugar, soap, cooking oil) and travel supplement, the combined value of which is estimated to be approximately $4–5 per visit.
- Provide a card to remind them of the date and time of their next visit.

## Bi-Monthly Visits (Days 14, 28, 42, 56, 70, 84, 98, 112, 126, 140, 154)

Participants will be scheduled in advance for a follow-up visit at the same health center every 2 weeks. Activities at each visit include:

- Confirm participant identify and study number.
- Administer questionnaire reviewing changes in medical history since the last visit.
- Record adverse events as reported by subject or observed by investigator.
- Measure and record vital signs, including:
  - Child’s height, weight and MUAC
  - Child’s axillary temperature
- Draw 50 µL capillary blood by finger-prick or heel stick from the child to create DBS for qPCR testing at a later date.
- Provide an in-kind incentive (e.g., sugar, soap, cooking oil) and travel supplement, the combined value of which is estimated to be approximately $4–5 per visit.
- Provide a card to remind them of the date and time of their next visit.

The following visits will have additional activities:

- **Visits on Day 28, 56, 70, 98, 126, 154**: Retreatment of *lesus*
- **Visits on Day 56 and 98**: Punch from *lesus* to measure permethrin content
- **Visit on Day 84**: Venous blood draws (mother/child) instead of capillary to:
  - Perform testing for Hb concentration
  - Create DBS for qPCR testing at a later date.
  - Store sera for future seroprevalence studies of vector-borne disease

## Final Study Visit (Day 168)

The final study visit will include:

- Confirm participant identify and study number.
- Administer questionnaire reviewing changes in medical history since the last visit.
- Record adverse events as reported by subject or observed by investigator.
- Measure and record vital signs, including:
  - Child’s height, weight and MUAC
  - Child’s axillary temperature
- Have 3–4 mL of blood drawn by venous phlebotomy from the child to:
  - Perform testing for hemoglobin concentration
  - Create dried blood spots (DBS) for qPCR testing at a later date.
  - Store sera for future seroprevalence studies of vector-borne disease
- Have 6–8 mL of blood drawn by venous phlebotomy from the mother to:
  - Perform testing for Hb concentration
  - Create DBS for qPCR testing at a later date.
  - Store sera for future seroprevalence studies of vector-borne disease
- Obtain punch from *lesus* to measure permethrin content.
- Complete semi-structured exit interview.
- Provide study completion incentive of approximately $20.

## Unscheduled Visits

Participants will be instructed to present to their respective study clinic for any acute febrile illness or other symptoms that may be consistent with malaria (e.g., poor feeding, headache, malaise). Upon registration, participants will present their study ID card to health center staff, who will subsequently notify study staff of the visit. Participants will undergo routine evaluation, testing, and treatment in accordance with local protocols. At the conclusion of the visit, a staff member will interview the patient to document symptoms and review clinic and laboratory registers to abstract relevant information. Of note, the study will maintain a modest supply of RDTs and artemether-lumefantrine (AL) to ensure there is no shortage of diagnostic tests or treatment.

# STUDY PROCEDURES /EVALUATIONS

## Study Procedures/Evaluations

### Demographic and Medical History

During the household visit, staff will record the full name, date-of-birth, sex, educational history, occupation, and other demographic characteristics of the participant and child. Relevant medical history collected will include obstetric (or birth) history, current diagnoses and medications, and any history of hospitalizations. At subsequent visits, staff will enquire about interval history (i.e., previous 2 weeks) including (i) the presence of any symptoms consistent with malaria (e.g., fever, headache, lethargy, poor-feeding, diarrhea), (ii) care-seeking in the public or private sector, (iii) treatments received, and (iv) potential adverse reactions to the intervention.

### Household

Study staff will record the location of the household using a handheld GPS device and administer a questionnaire modified from the most recent Demographic and Health Survey [21] covering topics including:

- The age, sex, and relationship to the participant of each household member
- Name of the CHW responsible for the household
- Household characteristics (e.g., rooms, materials, water sources, toilet, etc.)
- Indices of socioeconomic status including ownership of items such as a cell phone, motorcycle, etc.
- Malaria knowledge, care-seeking patterns, and prevention behaviors

### Physical examinations

Study staff will measure vital signs including height, weight, MUAC, and axillary temperature at each visit. A clinical officer will perform a general physical examination, including full inspection of the skin, of all participating children at the baseline visit. At subsequent visits, a clinical officer will be available to perform focused clinical assessments, but only in response to adverse events reported by participants or staff.

### Biological specimens

Trained study staff trained will perform all blood collection procedures in accordance with established methods and study-specific standard operating procedures. Blood collection at the baseline (Day 0), mid-point (Day 84), and final visits will be performed by venous phlebotomy with the goal of collecting 7–8 mL of whole blood from mothers and 3–4 mL from children. Aliquots of whole blood will be removed from the vacutainer by pipette for point-of-care testing and creation of DBS. Remnant blood will be processed for long-term storage (see Section 7.2.3). All other collections will be capillary blood specimens obtained by finger- or heel-stick with the goal of obtaining approximately 50 µL.

### Self-reported data

Participants will be asked to keep daily logs (“diaries”) documenting the frequency of:

- Mother and child sleeping under LLIN
- *Lesu* use throughout day, divided into morning, afternoon, and evening
- *Lesu* washing

### Observation logs

Study staff will perform direct observation of a random sample of participants (25%) to document how and when *lesus* are used throughout the day with particular emphasis on the morning and evening periods. Staff will also enquire how participants wash the *lesus* and confirm that LLINs are being used.

### Entomological surveillance

Staff will conduct sentinel surveillance in at least 20% of participant households. Randomization will be in a 1:1 ratio between intervention and control arms with households stratified by village of residence in order to generate high-resolution estimates of mosquito abundance over the study area. Surveillance will occur one night every 2 weeks. To monitor adult mosquitoes, we will employ unbaited (indoor) and CO_2_-baited (outdoor) CDC light traps. All captured mosquitoes will be counted, sexed, and identified to species.

## Laboratory Procedures/Evaluations

### Screening Laboratory Evaluations

- HIV: Testing for HIV will be performed at the household visit with a rapid diagnostic test (SD Bioline HIV-1/2 3.0 Abbott Laboratories, USA) [43]. Approximately 20 µL of whole blood will be collected via finger-prick and placed onto the RDT in accordance with the manufacturer’s instructions.

### Clinical Laboratory Evaluations

- Malaria: Testing for malaria will be performed at the household visit with an RDT (SD Bioline Malaria Ag P.f, Abbott Laboratories, USA or similar pending availability). Approximately 100 µL of whole blood will be collected via finger-prick or heel stick and placed onto the RDT in accordance with the manufacturer’s instructions. This test detects histidine-rich protein II antigen specific to *P. falciparum* malaria. Similar assays are currently employed for routine diagnosis in Uganda [44].
- Hemoglobin: Hb levels will measured at the study clinics using the HemoCue® Hb 201+ analyzer (Brea, California) [45, 46]. Approximately 10 µL of whole blood will be placed onto the microcuvette in accordance with the manufacturer’s instructions. Results are generally available within 1–2 minutes.
- Asymptomatic Parasitemia: DBS will be stored in a mylar bag with desiccant until transport to Epicentre Research Laboratory in Mbarara. *Plasmodium* species DNA will be extracted from DBS using a previously described protocols [47]. The concentration of extracted *P. falciparum* DNA in individual samples will be determined using qPCR for *P. falciparum* lactate dehydrogenase [48].

### Special Assays or Procedures

- Permethrin Concentration: Harvested fabric swatches will be placed in individual mylar bags and stored in an opaque container at 20^o^ C until transport to the Richards Lab at East Carolina University (see attached letter). Swatches will be transferred to individual 60 mL amber glass vials containing 40 mL acetone and soaked for 6 hours to elute permethrin. A portion of the extract (1 pL) will be analyzed directly by capillary GC with flame ionization detector using an Agilent GC 6850 in accordance with previously published protocols [35, 41].

### Specimen Preparation, Handling, and Storage

Information regarding preparation, handling, labeling, and storage of specimens is available in the *Manual of Procedures*.

### Specimen Shipment

Information regarding method and frequency of specimen shipment is available in the *Manual of Procedures*. Transportation of specimens will be recorded in chain-of-custody logs.

# ASSESSMENT OF SAFETY

## Specification of Safety Parameters

Given the established safety of permethrin across applications (e.g., topical, nets, clothing), we anticipate few, if any, adverse events. Therefore, monitoring of any events will be through routine reporting mechanisms. The following measures will be recorded in the study database and will require notification of the PI if exceeding the specified threshold.

| **Parameter** | **Threshold** |
| --- | --- |
| MUAC | MUAC ≤11 cm |
| Hemoglobin | Concentration ≤5 g/dL for children and ≤7 g/dL for adults |
| Skin lesion | Presence of any cutaneous skin lesion or rash |
| Hospitalization | Any reason |

### Unanticipated Problems

Unanticipated problems involving risks to subjects or others include, in general, any incident, experience, or outcome that meets **all** of the following criteria:

- unexpected in terms of nature, severity, or frequency given (a) the research procedures that are described in the protocol-related documents, such as the IRB-approved research protocol and ICF; and (b) the characteristics of the subject population being studied;
- related or possibly related to participation in the research (“possibly related” means there is a reasonable possibility that the incident, experience, or outcome may have been caused by the procedures involved in the research); and
- suggests that the research places subjects or others at a greater risk of harm (including physical, psychological, economic, or social harm) than was previously known or recognized.

### Adverse Events

An adverse event (AE) is any untoward or unfavorable medical occurrence in a human subject, including any abnormal sign (for example, abnormal physical exam or laboratory finding), symptom, or disease, temporally associated with the subject’s participation in the research, whether or not considered related to the subject’s participation in the research.

### Serious Adverse Events

A serious adverse event (SAE) is one that meets one or more of the following criteria:

- Results in death
- Is life-threatening (places the subject at immediate risk of death from the event as it occurred)
- Results in inpatient hospitalization or prolongation of existing hospitalization
- Results in a persistent or significant disability or incapacity
- Results in a congenital anomaly or birth defect
- An important medical event that may not result in death, be life threatening, or require hospitalization may be considered an SAE when, based upon appropriate medical judgment, the event may jeopardize the subject and may require medical or surgical intervention to prevent one of the outcomes listed in this definition.

## Time Period and Frequency for Event Assessment and Follow-Up

Unanticipated problems will be recorded in the data collection system throughout the study. The PI will record all reportable events with start dates occurring any time after informed consent is obtained until 7 (for non-serious AEs) or 30 days (for SAEs) after the last day of study participation. At each study visit, the investigator will inquire about the occurrence of AE/SAEs since the last visit. Events will be followed for outcome information until resolution or stabilization.

## Characteristics of an Adverse Event

### Relationship to Study Intervention

To assess the relationship of an event to the study intervention, the following guidelines are used:

- Related (Possible, Probable, Definite)
  - The event is known to occur with the study intervention.
  - There is a temporal relationship between the intervention and event onset.
  - The event abates when the intervention is discontinued.
  - The event reappears upon a re-challenge with the intervention.
- Not Related (Unlikely, Not Related)
  - There is no temporal relationship between intervention and event.
  - An alternate etiology has been established.

### Expectedness of SAEs

The study PI will be responsible for determining whether an SAE is expected or unexpected. An adverse event will be considered unexpected if the nature, severity, or frequency of the event is not consistent with the risk information previously described for the intervention.

### Severity of Event

The following scale will be used to grade adverse events:

- **Mild**: no intervention required; no impact on activities of daily living (ADL)
- **Moderate**: minimal, local, or non-invasive intervention indicated; moderate impact on ADL
- **Severe**: significant symptoms requiring invasive intervention; subject seeks medical attention, needs major assistance with ADL

## Reporting Procedures

### Unanticipated Problem Reporting to IRB

Incidents or events that meet the criteria for unanticipated problems require the creation and completion of an unanticipated problem report form. The following information will be included when reporting an adverse event, or any other incident, experience, or outcome as an unanticipated problem to the IRB:

- appropriate identifying information for the research protocol, such as the title, investigator’s name, and the IRB project number;
- a detailed description of the adverse event, incident, experience, or outcome;
- an explanation of the basis for determining that the adverse event, incident, experience, or outcome represents an unanticipated problem;
- a description of any changes to the protocol or other corrective actions that have been taken or are proposed in response to the unanticipated problem.

To satisfy the requirement for prompt reporting, unanticipated problems will be reported using the following timeline:

- Unanticipated problems that are serious adverse events will be reported to the IRB within 1 week of the investigator becoming aware of the event.
- Any other unanticipated problem will be reported to the IRB within 2 weeks of the investigator becoming aware of the problem.
- All unanticipated problems should be reported to appropriate institutional officials (as required by an institution’s written reporting procedures) within 1 month of the IRB’s receipt of the report of the problem from the investigator.

### Reporting of Pregnancy

The EPA has stated that, “Based on our review of scientific studies, there is no evidence of reproductive or developmental effects to mother or child following exposure to permethrin [36].” In addition, the CDC recommends the use of permethrin-treated clothing by pregnant women in regions at risk for mosquito-borne diseases [26]. Therefore, there will not be any monitoring for, reporting of, or modifications to study eligibility relating to pregnancy status.

## Halting Rules

The occurrence of any severe adverse event or at least five study-related moderate adverse events will prompt a temporary suspension of enrollment pending review by the Data Safety & Monitoring Board. The objective of the safety review is to decide whether the study should continue per protocol, proceed with caution, be further investigated, be discontinued, or be modified and then proceed. Suspension of enrollment is a potential outcome of a safety review.

# STUDY OVERSIGHT

In addition to the PI’s responsibility for oversight, study oversight will be under the direction of the Data Safety and Monitoring Board (DSMB) at the North Carolina Translational & Clinical Sciences Institute (TraCS), led by Dr. Ross Simpson. The DSMB is independent of the study and will be available in real time to review and recommend appropriate action regarding adverse events and other safety issues will review enrollment reports every 6 months and full data reports annually. Any moderate or severe adverse events related to study participation should be reported promptly and an ad hoc review be conducted, if deemed necessary. Reviewed data will be separated by study arm and provided by a statistician with access to the unblinded data.

# STATISTICAL CONSIDERATIONS

## Study Hypotheses

We hypothesize that the intervention will reduce malaria incidence among infants and young children relative to children receiving untreated *lesus*.

## Sample Size Considerations

The sample size was selected to provide sufficient power to evaluate the primary study hypothesis. We plan to recruit and enroll 400 mother-infant pairs. Additional participants will be enrolled in the study to replace those who drop out before 12 weeks. Those who drop out after 12 weeks will not be replaced. All participants who attend any clinic visits post randomization will be included in the primary, intention-to-treat (ITT) analysis.

Assuming a baseline risk of approximately 3.0 cases per 100 person-weeks as estimated from our pilot study, the trial will have power of at least 0.80 to detect a 30% relative reduction in the incidence rate of malaria between intervention and control groups over the proposed study period of 24 weeks with a type I error rate of 0.05 and assuming a two-sided test based on the asymptotic test statistic of the intervention parameter from a Poisson regression model with an offset for time at risk [49]. Power for other effect sizes is presented in the table below. This assumes an average of 23.1 weeks of follow-up per participant per arm, based on the observed drop-out in the pilot study. Calculations were conducted using nQuery software and are based on previously described methods [50].

No interim analyses are proposed, so no adjustments were made to these calculations to account for interim testing. This effect size was assessed as the minimum at which the intervention would be persuasive to take to scale, especially as it represents an incremental effect above and beyond LLIN use.

Anticipated Power to Detect Differences in Malaria Incidence Rate between Intervention and Control Arms

| **Rate Ratio** | **Anticipated Power** |
| --- | --- |
| 0.80 | 0.42 |
| 0.75 | 0.62 |
| 0.70 | 0.80 |
| 0.65 | 0.92 |

## Final Analysis Plan

Analyses will be conducted using an ITT approach, with woman-infant pairs analyzed according to the arm they were randomly assigned to (i.e., treated or untreated *lesu*) regardless of their subsequent use or non-use of the *lesus* and dropout. *Lesu* use will be evaluated as a secondary outcome, and if *lesu* use is lower than anticipated, exploratory analyses may use marginal structural models to estimate an “as-treated” effect of permethrin-treated *lesus* on the primary endpoint.

An α=0.05 significance level will be used throughout, with corresponding 95% confidence intervals. No adjustment for multiple testing will be made for the primary outcome. For secondary outcomes (excluding safety endpoints), we will control the false discovery rate using methods from Benjamini and Hochberg (1995).

Given the level of subject participation in the pilot study, we expect most mother-infant pairs to complete all study activities. Missing data due to dropout or missed clinic visits are anticipated to be uncommon (≤5% missing), so a complete case analysis is planned. If >10% of participants are missing data in either arm, a sensitivity analysis will be conducted using multiple imputation assuming data are missing at random (missing conditional upon measured baseline covariates and study outcomes). If data are suspected to be missing not at random, this will be described with the study results as a limitation.

### Analysis of primary outcome (AIM 1)

In the primary analysis, the rates of clinical *P. falciparum* malaria in children 6–24 months of age will be compared between treatment arms by estimating the incidence rate ratio and a corresponding 95% confidence interval (with the control arm as the referent group) of malaria using a Poisson regression model. The outcome, the number of observed incident cases of malaria, is a count variable. Individuals will be censored from the analysis for a period of 28 days after an incident malaria episode to account for the reduced risk of infection following treatment with AL. The Poisson model will include adjustments to control for stratification variables from randomization (study site and age group) and will include an offset to account for varying person-time-at-risk across subjects. The robust variance estimator will be used to account for potential overdispersion.

In exploratory analyses, we will further evaluate our primary outcome by fitting additional Poisson regression models to estimate associations between the incidence of malaria and measured covariates, including the frequency of *lesu* use and washing and residual permethrin concentration among participants in the intervention arm.

### Analysis of secondary outcomes (AIM 2)

- Change in child’s Hb level from baseline to weeks 12 and 24, Change in child’s growth from baseline to weeks 12 and 24, Change in mother’s Hb level from baseline to weeks 12 and 24: For each endpoint, Student’s t tests will be used to compare mean changes between study groups at 12 and 24 weeks, with corresponding 95% CIs. Wilcoxon rank sum tests will be conducted as a sensitivity analysis. Participants with missing data at a given timepoint will be excluded from the analysis if missing data is minimal (<10% in both arms). If missing data are more common than anticipated, multiple imputation will be conducted.
- Asymptomatic parasitemia in children, Clinical malaria in mothers: Each endpoint will be analyzed using the same methods as the primary analysis of the primary endpoint. As in the primary analysis, ITT analyses with complete data are expected, but sensitivity analyses will be conducted if dropout or missing data are more common than anticipated or if adherence is lower than expected.
- Hospitalization for malaria: the number of hospitalizations for malaria in the treatment and control arms will be compared using Fisher’s exact test. Confidence intervals for the proportion of hospitalizations and the difference in proportions between randomization arms will be computed using Clopper-Pearson exact binomial confidence intervals.
- Safety measures (adverse reaction to treated-*lesu,* SAE): Descriptive analyses will be used to summarize adverse reactions to treated-*lesu* and SAEs, by treatment arm. A 95% 1-sided Clopper-Pearson exact binomial upper confidence limit for the probability of an SAE will be computed within each arm, and if sample sizes allow, by child’s age group.

# SOURCE DOCUMENTS AND ACCESS TO SOURCE DATA/DOCUMENTS

Study staff will maintain appropriate medical and research records for this study, in compliance with ICH E6, Section 4.9 and regulatory and institutional requirements for the protection of confidentiality of subjects. Study staff will permit authorized representatives of regulatory agencies to examine (and when required by applicable law, to copy) research records for the purposes of quality assurance reviews, audits, and evaluation of the study safety, progress, and data validity.

# QUALITY CONTROL AND QUALITY ASSURANCE

Each site will have a copy of the standard operating procedures (SOPs) that describe:

- How participants will be consented and enrolled
- How procedures and laboratory testing will be conducted to ensure both safety and quality.
- The documents to be reviewed (e.g., consent forms case report forms, clinic notes), who is responsible, and the frequency for reviews.
- Who will be responsible for reviewing data and addressing quality assurance issues (correcting procedures that are not in compliance with protocol) and quality control issues (correcting errors in data entry).
- Staff training methods and how such training will be tracked.

At minimum, the PI and study biostatistical team will be responsible for reviewing a subset of data entry forms in the electronic database at least weekly. Additional levels of review will be conducted at each site to ensure data fidelity.

# ETHICS/PROTECTION OF HUMAN SUBJECTS

## Ethical Standard

The PI will ensure that this study is conducted in full conformity with the principles set forth in (i) The Belmont Report: Ethical Principles and Guidelines for the Protection of Human Subjects of Research, as drafted by the U.S. National Commission for the Protection of Human Subjects of Biomedical and Behavioral Research (April 18, 1979) and codified in 45 CFR Part 46 and (ii) The Ugandan National Drug Policy and Authority (Conduct of Clinical Trials) Regulations, 2014.

## Institutional Review Board

The protocol, ICF(s), recruitment materials, and all subject materials will be submitted to the Mbarara University of Science and Technology Research Ethics Committee (REC), the Uganda National Council of Science and Technology (UNCST), and the UNC IRB for review and approval. Approval of both the protocol and the consent form must be obtained before any subject is enrolled. Any amendment to the protocol will require review and approval by the IRB before the changes are implemented in the study.

## Informed Consent Process

Informed consent is a process that is initiated prior to the individual agreeing to participate in the study and continues throughout study participation. Extensive discussion of risks and possible benefits of study participation will be provided to subjects and their families, if applicable. A consent form describing in detail the study procedures and risks will be given to the subject. Consent forms will be IRB-approved and available in English or Lukhonzo. The subject is required to read and review the document or have the document read to him or her. The investigator or designee will explain the research study to the subject and answer any questions that may arise.

The subject will sign the ICF prior to any study-related assessments or procedures. Subjects will be given the opportunity to discuss the study with their surrogates or think about it prior to agreeing to participate. They may withdraw consent at any time throughout the course of the study. A copy of the signed ICF will be given to subjects for their records. The rights and welfare of the subjects will be protected by emphasizing to them that the quality of their clinical care will not be adversely affected if they decline to participate in this study. The consent process will be documented in the clinical or research record.

## Exclusion of Women, Minorities, and Children (Special Populations)

The study specifically examines the effect of the intervention on malaria in women (mothers) and young children.

## Subject Confidentiality

Subject confidentiality is strictly held in trust by the investigators, study staff, and the sponsor(s) and their agents. This confidentiality is extended to cover testing of biological samples and genetic tests in addition to any study information relating to subjects.

The study protocol, documentation, data, and all other information generated will be held in strict confidence. No information concerning the study, or the data will be released to any unauthorized third party without prior written approval of the sponsor.

The regulatory authorities or other representatives may inspect all study documents and records required to be maintained by the investigator, including but not limited to, medical records (office, clinic, or hospital) for the study subjects. The clinical study site will permit access to such records.

## Future Use of Stored Specimens and Other Identifiable Data

Remnant specimens including whole blood, sera, and DBS with associated coded, but identifiable data will be maintained at the MUST-UNC Research Office in Mbarara for a period of up to five years after the study is complete. Specimens will only be used for research. No commercial (i.e., for-profit) applications will be allowed. Participants will be informed and have the opportunity to opt out of long-term storage on the consent form. Any future research use will require approval of the relevant IRBs.

# DATA HANDLING AND RECORD KEEPING

The investigators are responsible for ensuring the accuracy, completeness, legibility, and timeliness of the data reported. All source documents should be completed in a neat, legible manner to ensure accurate interpretation of data. The investigators will maintain adequate case histories of study subjects, including accurate case report forms, and source documentation.

## Data Management Responsibilities

Data collection and accurate documentation are the responsibility of the study staff under the supervision of the investigator. All source documents and laboratory reports must be reviewed by the study team and data entry staff, who will ensure that they are accurate and complete. Unanticipated problems and adverse events must be reviewed by the investigator or designee.

## Data Capture Methods

All data will be entered into portable tablet devices equipped with wireless internet and uploaded each day to a secure, online database (e.g., UNC REDCap). Each week, new entries will be reviewed by a research assistant and any errors or incomplete entries will be forwarded to the field team for correction.

## Study Records Retention

Study records will be maintained for at least five years from the date that the final report is submitted to the sponsor. No records will be destroyed without the written consent of the IRB.

## Protocol Deviations

A protocol deviation is any noncompliance with the clinical study protocol, Good Clinical Practice, or Manual of Procedures requirements. The noncompliance may be on the part of the subject, the investigator, or study staff. As a result of deviations, corrective actions are to be developed by the study staff and implemented promptly.

These practices are consistent with investigator and sponsor obligations in ICH E6:

- Compliance with Protocol, Sections 4.5.1, 4.5.2, 4.5.3, and 4.5.4.
- Quality Assurance and Quality Control, Section 5.1.1
- Noncompliance, Sections 5.20.1 and 5.20.2.

All deviations from the protocol must be addressed in study subject source documents and promptly reported to the local IRB, according to their requirements.

# PUBLICATION/DATA SHARING POLICY

This study will comply with the [National Institutes of Health (NIH) Public Access Policy](http://publicaccess.nih.gov/policy.htm), which ensures that the public has access to the published results of NIH-funded research. It requires scientists to submit final peer-reviewed journal manuscripts that arise from NIH funds to the digital archive [PubMed Central](http://www.pubmedcentral.nih.gov/) upon acceptance for publication.

The International Committee of Medical Journal Editors (ICMJE) member journals have adopted a clinical trials registration policy as a condition for publication. The ICMJE defines a clinical trial as any research project that prospectively assigns human subjects to intervention or concurrent comparison or control groups to study the cause-and-effect relationship between a medical intervention and a health outcome. Medical interventions include drugs, surgical procedures, devices, behavioral treatments, process-of-care changes, and the like. Health outcomes include any biomedical or health-related measures obtained in patients or participants, including pharmacokinetic measures and adverse events. The ICMJE policy requires that all clinical trials be registered in a public trials registry such as [ClinicalTrials.gov](http://www.clinicaltrials.gov), which is sponsored by the National Library of Medicine. Other biomedical journals are considering adopting similar policies. For interventional clinical trials performed under NIDCR grants and cooperative agreements, it is the grantee’s responsibility to register the trial in an acceptable registry, so the research results may be considered for publication in ICMJE member journals. The ICMJE does not review specific studies to determine whether registration is necessary; instead, the committee recommends that researchers who have questions about the need to register err on the side of registration or consult the editorial office of the journal in which they wish to publish.

# LITERATURE REFERENCES

1. Gething PW, Casey DC, Weiss DJ, Bisanzio D, Bhatt S, Cameron E, et al. Mapping Plasmodium falciparum Mortality in Africa between 1990 and 2015. N Engl J Med. 2016. Epub 2016/10/11. doi: 10.1056/NEJMoa1606701. PubMed PMID: 27723434.

2. Griffin JT, Hollingsworth TD, Okell LC, Churcher TS, White M, Hinsley W, et al. Reducing Plasmodium falciparum malaria transmission in Africa: a model-based evaluation of intervention strategies. PLoS Med. 2010;7(8). Epub 2010/08/17. doi: 10.1371/journal.pmed.1000324. PubMed PMID: 20711482; PubMed Central PMCID: PMCPMC2919425.

3. Smith DL, McKenzie FE, Snow RW, Hay SI. Revisiting the basic reproductive number for malaria and its implications for malaria control. PLoS Biol. 2007;5(3):e42. Epub 2007/02/22. doi: 10.1371/journal.pbio.0050042. PubMed PMID: 17311470; PubMed Central PMCID: PMCPMC1802755.

4. Ferguson HM, Dornhaus A, Beeche A, Borgemeister C, Gottlieb M, Mulla MS, et al. Ecology: a prerequisite for malaria elimination and eradication. PLoS Med. 2010;7(8):e1000303. Epub 2010/08/07. doi: 10.1371/journal.pmed.1000303. PubMed PMID: 20689800; PubMed Central PMCID: PMCPMC2914634.

5. Geissbuhler Y, Chaki P, Emidi B, Govella NJ, Shirima R, Mayagaya V, et al. Interdependence of domestic malaria prevention measures and mosquito-human interactions in urban Dar es Salaam, Tanzania. Malar J. 2007;6:126. Epub 2007/09/21. doi: 10.1186/1475-2875-6-126. PubMed PMID: 17880679; PubMed Central PMCID: PMCPMC2039744.

6. Tirados I, Costantini C, Gibson G, Torr SJ. Blood-feeding behaviour of the malarial mosquito Anopheles arabiensis: implications for vector control. Med Vet Entomol. 2006;20(4):425-37. Epub 2007/01/04. doi: 10.1111/j.1365-2915.2006.652.x. PubMed PMID: 17199754.

7. Huho B, Briet O, Seyoum A, Sikaala C, Bayoh N, Gimnig J, et al. Consistently high estimates for the proportion of human exposure to malaria vector populations occurring indoors in rural Africa. Int J Epidemiol. 2013;42(1):235-47. Epub 2013/02/12. doi: 10.1093/ije/dys214. PubMed PMID: 23396849; PubMed Central PMCID: PMCPMC3600624.

8. Musiime AK, Smith DL, Kilama M, Rek J, Arinaitwe E, Nankabirwa JI, et al. Impact of vector control interventions on malaria transmission intensity, outdoor vector biting rates and Anopheles mosquito species composition in Tororo, Uganda. Malar J. 2019;18(1):445. Epub 2019/12/29. doi: 10.1186/s12936-019-3076-4. PubMed PMID: 31881898; PubMed Central PMCID: PMCPMC6935116.

9. WHO. World Malaria Report 2019. Geneva: World Health Organization, 2019.

10. WHO. World Malaria Report 2020. Geneva: World Health Organization, 2020.

11. U.S. President’s Malaria Initiative. Malaria Operational Plan FY 2020. Washington, DC: U.S. President’s Malaria Initiative, 2020.

12. Okia M, Hoel DF, Kirunda J, Rwakimari JB, Mpeka B, Ambayo D, et al. Insecticide resistance status of the malaria mosquitoes: Anopheles gambiae and Anopheles funestus in eastern and northern Uganda. Malar J. 2018;17(1):157. Epub 2018/04/08. doi: 10.1186/s12936-018-2293-6. PubMed PMID: 29625585; PubMed Central PMCID: PMCPMC5889576.

13. Lynd A, Gonahasa S, Staedke SG, Oruni A, Maiteki-Sebuguzi C, Dorsey G, et al. LLIN Evaluation in Uganda Project (LLINEUP): a cross-sectional survey of species diversity and insecticide resistance in 48 districts of Uganda. Parasit Vectors. 2019;12(1):94. Epub 2019/03/15. doi: 10.1186/s13071-019-3353-7. PubMed PMID: 30867018; PubMed Central PMCID: PMCPMC6417037.

14. Ojuka P, Boum Y, 2nd, Denoeud-Ndam L, Nabasumba C, Muller Y, Okia M, et al. Early biting and insecticide resistance in the malaria vector Anopheles might compromise the effectiveness of vector control intervention in Southwestern Uganda. Malar J. 2015;14:148. Epub 2015/04/17. doi: 10.1186/s12936-015-0653-z. PubMed PMID: 25879539; PubMed Central PMCID: PMCPMC4416237.

15. WHO. Control of residual malaria parasite transmission: Guidance Note - September 2014. Geneva: World Health Organization, 2014.

16. WHO. Global technical strategy for malaria 2016-2030. Geneva: World Health Organization, 2015.

17. Roll Back Malaria Partnership Secretariat. Action and investment to defeat malaria 2016–2030. For a malaria-free world. . Geneva: World Health Organization, 2015.

18. malERA Consultative Panel on Tools for Malaria Elimination. malERA: An updated research agenda for diagnostics, drugs, vaccines, and vector control in malaria elimination and eradication. PLoS Med. 2017;14(11):e1002455. Epub 2017/12/01. doi: 10.1371/journal.pmed.1002455. PubMed PMID: 29190291; PubMed Central PMCID: PMCPMC5708606.

19. Londono-Renteria B, Patel JC, Vaughn M, Funkhauser S, Ponnusamy L, Grippin C, et al. Long-Lasting Permethrin-Impregnated Clothing Protects Against Mosquito Bites in Outdoor Workers. Am J Trop Med Hyg. 2015;93(4):869-74. Epub 2015/07/22. doi: 10.4269/ajtmh.15-0130. PubMed PMID: 26195460; PubMed Central PMCID: PMCPMC4596613.

20. Vaughn MF, Funkhouser SW, Lin FC, Fine J, Juliano JJ, Apperson CS, et al. Long-lasting permethrin impregnated uniforms: A randomized-controlled trial for tick bite prevention. Am J Prev Med. 2014;46(5):473-80. Epub 2014/04/22. doi: 10.1016/j.amepre.2014.01.008. PubMed PMID: 24745637.

21. Uganda Bureau of Statistics (UBOS) and ICF. Uganda Demographic and Health Survey Kampala, Uganda and Rockville, Maryland: UBOS and ICF., 2018.

22. Berkley J, Mwangi I, Griffiths K, Ahmed I, Mithwani S, English M, et al. Assessment of severe malnutrition among hospitalized children in rural Kenya: comparison of weight for height and mid upper arm circumference. Jama. 2005;294(5):591-7. Epub 2005/08/04. doi: 10.1001/jama.294.5.591. PubMed PMID: 16077053.

23. Armed Forces Pest Management Board. Technical Guide No. 36: Personal Protective Measures against Insects and other Arthropods of Military Significance. Silver Spring, MD: Armed Forces Pest Management Board, 2015.

24. Kegel P, Letzel S, Rossbach B. Biomonitoring in wearers of permethrin impregnated battle dress uniforms in Afghanistan and Germany. Occup Environ Med. 2014;71(2):112-7. Epub 2013/12/18. doi: 10.1136/oemed-2012-101279. PubMed PMID: 24343973.

25. Proctor SP, Maule AL, Heaton KJ, Adam GE. Permethrin exposure from fabric-treated military uniforms under different wear-time scenarios. J Expo Sci Environ Epidemiol. 2014;24(6):572-8. Epub 2013/10/10. doi: 10.1038/jes.2013.65. PubMed PMID: 24104061.

26. Centers for Disease Control and Prevention. Zika virus: pregnancy Atlanta, GA: CDC; [cited 2018 July 2]. Available from: <https://www.cdc.gov/zika/pregnancy/>.

27. Bowman NM, Akialis K, Cave G, Barrera R, Apperson CS, Meshnick SR. Pyrethroid insecticides maintain repellent effect on knock-down resistant populations of Aedes aegypti mosquitoes. PLoS One. 2018;13(5):e0196410. Epub 2018/05/16. doi: 10.1371/journal.pone.0196410. PubMed PMID: 29763445; PubMed Central PMCID: PMCPMC5953453.

28. Kittayapong P, Olanratmanee P, Maskhao P, Byass P, Logan J, Tozan Y, et al. Mitigating Diseases Transmitted by Aedes Mosquitoes: A Cluster-Randomised Trial of Permethrin-Impregnated School Uniforms. PLoS Negl Trop Dis. 2017;11(1):e0005197. Epub 2017/01/20. doi: 10.1371/journal.pntd.0005197. PubMed PMID: 28103255; PubMed Central PMCID: PMCPMC5245776.

29. Uganda Bureau of Statistics (UBOS). National Population and Housing Census 2014: Provisional Results. Kampala, Uganda: 2014.

30. Olupot W, AJ M, Plumptre A. An analysis of socioeconomics of bushmeat hunting at major hunting sites in Uganda. Bronx, NY: Wildlife Conservation Society, 2009 Contract No.: WORKING PAPER NO. 38.

31. Yeka A, Gasasira A, Mpimbaza A, Achan J, Nankabirwa J, Nsobya S, et al. Malaria in Uganda: challenges to control on the long road to elimination: I. Epidemiology and current control efforts. Acta Trop. 2012;121(3):184-95. Epub 2011/03/23. doi: 10.1016/j.actatropica.2011.03.004. PubMed PMID: 21420377; PubMed Central PMCID: PMCPMC3156969.

32. Uganda Bureau of Statistics (UBOS) and ICF International. Uganda Malaria Indicator Survey 2014-15. Kampala, Uganda, and Rockville, Maryland, USA: 2015.

33. Uganda National Malaria Control Division, Uganda Bureau of Statistics, and ICF. Uganda Malaria Indicator Survey 2018-19. Kampala, Uganda, and Rockville, Maryland, USA: 2020.

34. Krefis AC, Schwarz NG, Nkrumah B, Acquah S, Loag W, Sarpong N, et al. Principal component analysis of socioeconomic factors and their association with malaria in children from the Ashanti Region, Ghana. Malar J. 2010;9:201. Epub 2010/07/16. doi: 10.1186/1475-2875-9-201. PubMed PMID: 20626839; PubMed Central PMCID: PMCPMC2914064.

35. Richards SL, Balanay JAG, Harris JW, Banks VM, Meshnick S. Residual Effectiveness of Permethrin-Treated Clothing for Prevention of Mosquito Bites Under Simulated Conditions. J Environ Health. 2017;79(8):8-15. Epub 2017/11/18. PubMed PMID: 29148650.

36. Environmental Protection Agency. Repellent Treated Clothing 2017 [cited 2017 August 9]. Available from: <https://www.epa.gov/insect-repellents/repellent-treated-clothing>.

37. Kennedy D, Hurst V, Konradsdottir E, Einarson A. Pregnancy outcome following exposure to permethrin and use of teratogen information. Am J Perinatol. 2005;22(2):87-90. Epub 2005/02/26. doi: 10.1055/s-2005-837736. PubMed PMID: 15731987.

38. Mytton OT, McGready R, Lee SJ, Roberts CH, Ashley EA, Carrara VI, et al. Safety of benzyl benzoate lotion and permethrin in pregnancy: a retrospective matched cohort study. BJOG. 2007;114(5):582-7. Epub 2007/04/19. doi: 10.1111/j.1471-0528.2007.01290.x. PubMed PMID: 17439567.

39. World Health Organization. Breastfeeding and maternal medication: recommendations for drugs in the eleventh WHO model list of essential drugs. Geneva: WHO, 2002.

40. Sawyer Products. Safety Data Sheet: SAWYER® PERMETHRIN 40% Manufacturing Concentrate 2778. Sawyer Products; 2019.

41. Gupta RK, Rutledge LC, Reifenrath WG, Gutierrez GA, Korte DW, Jr. Effects of weathering on fabrics treated with permethrin for protection against mosquitoes. J Am Mosq Control Assoc. 1989;5(2):176-9. Epub 1989/06/01. PubMed PMID: 2568392.

42. Uganda Ministry of Health. National Guidelines for the Management of Common Conditions. Kampala, Uganda2016.

43. Vijayakumar TS, David S, Selvaraj K, Viswanathan T, Kannangai R, Sridharan G. Performance of a rapid immunochromatographic screening test for detection of antibodies to human immunodeficiency virus type 1 (HIV-1) and HIV-2: experience at a tertiary care hospital in South India. J Clin Microbiol. 2005;43(8):4194-6. Epub 2005/08/06. doi: 10.1128/jcm.43.8.4194-4196.2005. PubMed PMID: 16081975; PubMed Central PMCID: PMCPMC1233946.

44. Boyce RM, Muiru A, Reyes R, Ntaro M, Mulogo E, Matte M, et al. Impact of rapid diagnostic tests for the diagnosis and treatment of malaria at a peripheral health facility in Western Uganda: an interrupted time series analysis. Malar J. 2015;14:203. Epub 2015/05/15. doi: 10.1186/s12936-015-0725-0. PubMed PMID: 25971788; PubMed Central PMCID: PMCPMC4435913.

45. Cohen AR, Seidl-Friedman J. HemoCue® system for hemoglobin measurement. Evaluation in anemic and nonanemic children. Am J Clin Pathol. 1988;90(3):302-5. Epub 1988/09/01. doi: 10.1093/ajcp/90.3.302. PubMed PMID: 3414603.

46. Parker M, Han Z, Abu-Haydar E, Matsiko E, Iyakaremye D, Tuyisenge L, et al. An evaluation of hemoglobin measurement tools and their accuracy and reliability when screening for child anemia in Rwanda: A randomized study. PLoS One. 2018;13(1):e0187663. Epub 2018/01/05. doi: 10.1371/journal.pone.0187663. PubMed PMID: 29300737; PubMed Central PMCID: PMCPMC5754049.

47. Gorret AM, Muhindo R, Baguma E, Ntaro M, Mulogo EM, Deutsch-Feldman M, et al. Comparison of capillary versus venous blood for the diagnosis of Plasmodium falciparum malaria using rapid diagnostic tests. J Infect Dis. 2021. Epub 2021/01/28. doi: 10.1093/infdis/jiab032. PubMed PMID: 33502531.

48. Taylor SM, Juliano JJ, Trottman PA, Griffin JB, Landis SH, Kitsa P, et al. High-throughput pooling and real-time PCR-based strategy for malaria detection. J Clin Microbiol. 2010;48(2):512-9. Epub 2009/11/27. doi: 10.1128/jcm.01800-09. PubMed PMID: 19940051; PubMed Central PMCID: PMCPMC2815636.

49. Huffman MD. An Improved Approximate 2-Sample Poisson Test. J R Stat Soc C-Appl. 1984;33(2):224-6. PubMed PMID: WOS:A1984TQ05800013.

50. Gu K, Ng HKT, Tang ML, Schucany WR. Testing the ratio of two Poisson rates. Biometrical J. 2008;50(2):283-98. doi: 10.1002/bimj.200710403. PubMed PMID: WOS:000255262700011.

SUPPLEMENTAL MATERIALS

These documents are relevant to the protocol, but they are not considered part of the protocol. They are stored and modified separately. As such, modifications to these documents do not require protocol amendments.

- Site Roster
- Manual of Procedures
- Biosafety Precautions
- Laboratory Handling
- Case report forms
- Quality Management Plan
- Data Management Plan
- Statistical Analysis Plan

APPENDICES

APPENDIX A: SCHEDULE OF EVENTS

Appendix A1 – Schedule of Events for Participating Mothers

| **PROCEDURE** | | **STUDY ENCOUNTER** | | | | | | | | |
| --- | --- | --- | --- | --- | --- | --- | --- | --- | --- | --- |
|  |  | **Home** | **Home** | **Study Clinic** | | | | | | **Home** |
|  |  | **Information Session** | **Home Visit** | **Baseline** | **Bi-Weekly** | **Monthly** | **Mid-Point** | **Final Visit** | **Unscheduled Acute Visits** | **Other Encounters** |
| **Mother** | | | | | | | | | | |
| Enrollment | Recruitment | X |  |  |  |  |  |  |  |  |
|  | Eligibility Screen | X | X |  |  |  |  |  |  |  |
|  | Signed ICF |  | X |  |  |  |  |  |  |  |
|  | HIV Test |  | X |  |  |  |  |  |  |  |
|  | Malaria Test & Treat |  | X |  |  |  |  |  |  |  |
|  | Randomization |  | X |  |  |  |  |  |  |  |
| Self-Reported Data | Demographic & Health |  | X |  |  |  |  |  |  |  |
|  | Malaria KAP Survey |  | X |  |  |  |  |  |  |  |
|  | Health Update |  |  | X | X | X | X | X | X |  |
|  | Lesu Use/Washing Diary |  |  |  | X | X | X | X |  |  |
|  | Exit Interview |  |  |  |  |  |  | X |  |  |
| Observed Data | Household GPS |  | X |  |  |  |  |  |  |  |
|  | House Construction |  | X |  |  |  |  |  |  |  |
|  | Lesu Use* |  |  |  |  |  |  |  |  | X |
|  | Mosquito Trapping |  |  |  |  |  |  |  |  | X |
| Lab Testing | Venous Blood (10-15ml) |  |  | X |  |  | X | X |  |  |
|  | Hemoglobin |  |  | X |  |  | X | X |  |  |
|  | Malaria (PCR) |  |  | X |  |  | X | X |  |  |
|  | Salivary Antigen |  |  | X |  |  | X | X |  |  |

Appendix A2 – Schedule of Events for Participating Children and Lesus

| **PROCEDURE** | | **STUDY ENCOUNTER** | | | | | | | | |
| --- | --- | --- | --- | --- | --- | --- | --- | --- | --- | --- |
|  |  | **Home** | **Home** | **Study Clinic** | | | | | | **Home** |
|  |  | **Information Session** | **Home Visit** | **Baseline** | **Bi-Weekly** | **Monthly** | **Mid-Point** | **Final** | **Acute / Fever (Unscheduled)** | **Other** |
| **Child** | | | | | | | | | | |
| Enrollment | Eligibility Screen | X | X |  |  |  |  |  |  |  |
|  | Signed ICF (by mother) |  | X |  |  |  |  |  |  |  |
|  | Malaria Test & Treat |  | X |  |  |  |  |  |  |  |
|  | LLIN Distribution |  | X |  |  |  |  |  |  |  |
| Clinical | Health Update |  |  | X | X | X | X | X | X |  |
|  | Physical Exam |  |  | X |  |  |  |  | X |  |
|  | Height/Weight |  |  | X |  |  | X | X |  |  |
|  | MUAC |  |  | X |  |  | X | X |  |  |
| Lab Testing | Venous Blood (3-4 ml) |  |  | X |  |  | X | X |  |  |
|  | Hemoglobin |  |  | X |  |  | X | X |  |  |
|  | Malaria (PCR) |  |  | X |  |  | X | X |  |  |
|  | Salivary Antigen |  |  | X |  |  | X | X |  |  |
|  | DBS |  |  | X |  |  | X | X |  |  |
|  | Capillary Blood (10-50 µL) |  |  |  | X | X |  |  | X |  |
|  | DBS |  |  |  | X | X |  |  | X |  |
|  | Malaria (PCR) |  |  |  | X | X |  |  | X |  |
|  | Malaria (RDT) |  |  |  |  |  |  |  | X |  |
| **Lesu** | | | | | | | | | | |
|  | Issued to Participants |  |  | X |  |  |  |  |  |  |
|  | Treatment |  |  | X |  |  |  |  |  |  |
|  | Re-Treatment |  |  |  |  | X |  |  |  |  |
|  | Punches |  |  |  |  |  | X | X |  |  |
